# Supplementary material for: Personalized CRISPR knock-in cytokine gene therapy to remodel the tumor microenvironment and enhance CAR T cell therapy in solid tumors
Source: Nat Commun. 2025 Dec 9;16:10987. doi: 10.1038/s41467-025-67328-w (PMC12689812; doi:10.1038/s41467-025-67328-w)
Supplement: Supplementary file 1 — Supplementary Information [file 41467_2025_67328_MOESM1_ESM.pdf]

Supplementary Information for:

Personalized CRISPR Knock-In Cytokine Gene Therapy to Remodel the Tumor Microenvironment and Enhance CAR T Cell Therapy in Solid Tumors

Nature Communications

(Final submission, November 2025)

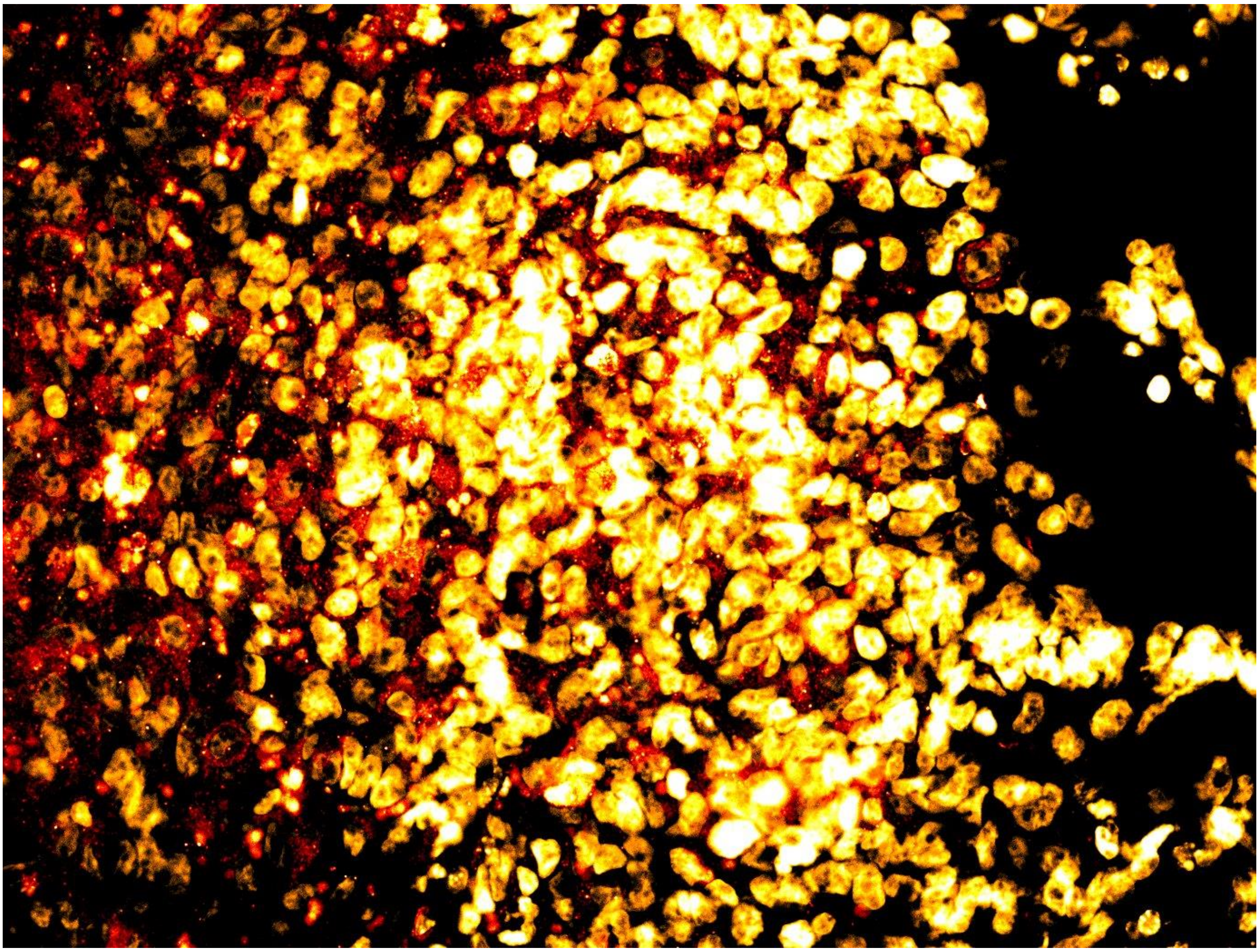

Supplementary Fig. 36. ***Ignite*****.**
Immunofluorescence micrograph of tumour-infiltrating CAR T cells within a CXCL10⁺ neuroblastoma xenograft. Nuclei and immune-cell infiltration are visualised by fluorescence microscopy. The image derives from the dataset shown in Fig. 6 and was submitted as a featured image suggestion to Nature Communications.

Table of Contents:

List of Abbreviations 4
Supplementary Notes 5
Supplementary Note 1 5
Supplementary Note 2 5
Supplementary Note 3 5
Supplementary Note 4 5
Supplementary Note 5 5
Supplementary Note 6 5
Supplementary Note 7 6
Supplementary Note 8 6
Supplementary Note 9 6
Supplementary Note 10 6
Supplementary Note 11 6
Supplementary Note 12 6
Supplementary Note 13 6
Supplementary Note 14 7
Supplementary Note 15 7
Supplementary Methods 8
Genomic DNA Sample Isolation 8
Standard PCR 8
Cell Lines and Cell Culture 8
Evaluation of Cell Proliferation and Viability Following CRISPR/Cas9-Mediated Gene Integration 8
Cytokine Quantification via ELISA 8
Flow Cytometry and Fluorescence-Activated Cell Sorting (FACS) 9
Boyden chamber and Clearview trans well migration assays 9
Cytotoxicity Assays 9
Macrophage Polarization Assay 10
Generation of L1CAM knock-out SK-N-BE2c 10
Immunohistochemistry (IHC) 10
Statistical estimation of tumor-specific CRISPR off-target risk 11
Supplementary Figures 12
Supplementary Fig. 1. CancerPAM multi-omics-based automated pipeline. 12
Supplementary Fig. 2. Dataset comparison between Terminate NB and DepMap. 13
Supplementary Fig. 3. Cell line data comparison between Terminate NB and DepMap data sets. 14
Supplementary Fig. 4. Chromosomal novel PAM distribution. 15
Supplementary Fig. 5. Novel PAM feature characteristics distribution. 16
Supplementary Fig. 6. CancerPAM ranking validation. 17
Supplementary Fig. 7. Feature correlation with PAM count. 18
Supplementary Fig. 8. Influence of MYCN amplification status on novel PAM counts and features. 19
Supplementary Fig. 9. In vitro CRISPR cutting and transgene vector production and testing. 20
Supplementary Fig. 10. In vitro CRISPR knock-in. 21
Supplementary Fig. 11. Site-specific knock-in confirmation via Sanger sequencing and PAM frequency analysis. 22
Supplementary Fig. 12. Site-specific knock-in analysis via dPCR and IFNG toxicity testing. 23

Supplementary Fig. 13. Unspecific knock-in. 24
Supplementary Fig. 14. dPCR analysis after FACS enrichment 25
Supplementary Fig. 15. Characterization of enriched transgenic tumor cell lines. 26
Supplementary Fig. 16. CAR T cell activation and exhaustion in tumor cell line-CAR T cell co-culture experiments. 27
Supplementary Fig. 17. Cytokine secretion in tumor cell line-CAR T cell co-culture experiments 28
Supplementary Fig. 18. Cytokine-mediated T cell migration in vitro 29
Supplementary Fig. 19. 3D tumor infiltration assay. 30
Supplementary Fig. 20. In vivo CAR T cell infiltration model development 31
Supplementary Fig. 21. In vivo CAR T cell infiltration experiment: pre-transplant and pre-treatment analysis. 32
Supplementary Fig. 22. Bioluminescence imaging of CAR T cell treated SK-N-AS xenograft neuroblastoma models. 33
Supplementary Fig. 23. Bioluminescence imaging of CAR T cell treated SK-N-BE2c xenograft neuroblastoma models. 34
Supplementary Fig. 24. In vivo CAR T cell infiltration in CXCL10-secreting xenograft tumors 35
Supplementary Fig. 25. Immunofluorescence staining of tumor sections 36
Supplementary Fig. 26. Flow cytometric analysis of IFNG-mediated regulation of surface markers in neuroblastoma cell lines. 37
Supplementary Fig. 27. Flow cytometric analysis of IFNG-mediated regulation of surface markers in neuroblastoma cell lines 38
Supplementary Fig. 28. Tumor cell line analysis prior to HuNOG transplantation 39
Supplementary Fig. 29. Phenotypic characterization of CAR T cells prior to infusion in HuNOG tumor-bearing mice 40
Supplementary Fig. 30. In vivo efficacy of CAR T cells against (CXCL10-expressing) SK-N-BE2c tumors in HuNOG mice. 41
Supplementary Fig. 31. Detection of CAR T cells in vivo by EGFR-reporter staining 42
Supplementary Fig. 32 Quantification of human immune and CAR T cell subsets in HuNOG mice. 43
Supplementary Fig. 33. CAR T cell bioluminescence imaging (BLI) in HuNOG mice and functional validation 44
Supplementary Fig. 34. Cytokine expression in neuroblastoma cell lines 45
Supplementary Fig. 35. Cytokine expression in patient tumors 46
Supplementary Tables 47
Supplementary Table 1. Exemplary patient data for top and bottom rank novel PAM sites identified via CancerPAM 47
Supplementary Table 2. Neuroblastoma cell line target loci 47
Supplementary Table 3. HDRT & gRNA sequences 48
Supplementary Table 4. Primer and probe sequences 50
Supplementary Table 5. Equipment and Consumables used 51
Supplementary Table 6. Antibodies used for flow cytometry and IF Staining 55
Supplementary Table 7. Software used 56
Supplementary Table 8. Potential effector cytokines for tumor microenvironment remodeling in neuroblastoma 56

List of Abbreviations

AAV - Adeno-associated virus
AUC - Area under the curve
BLI - Bioluminescence imaging
BSA - Bovine serum albumin
CAR - Chimeric antigen receptor
CFD - Cutting frequency determination
cDNA - Complementary DNA
CI - Confidence interval
CMV - Cytomegalovirus (used as a promoter in gene expression)
CRISPR - Clustered regularly interspaced short palindromic repeats
CXCL10 / CXCL11 - C-X-C motif chemokine ligand 10 / 11
DepMap - Cancer Dependency Map
dPCR - Digital polymerase chain reaction
dsDNA - Double-stranded DNA
EC DNA / ecDNA - Extrachromosomal DNA
EF1α - Elongation factor 1 alpha (used as a promoter in gene expression)
ELISA - Enzyme-linked immunosorbent assay
EGFRt - Truncated epidermal growth factor receptor
FACS - Fluorescence-activated cell sorting
FBS - Fetal bovine serum
FMO - Fluorescence minus one
GFP - Green fluorescent protein
gRNA - Guide RNA
Gy - Gray (unit of irradiation dose)
HDR - Homology-directed repair
HDRT - Homology-directed repair template (DNA donor)
HSC - Hematopoietic stem cell
HuNOG - Humanized NOG mouse
HUVEC - Human umbilical vein endothelial cells
HR - Hazard ratio
IF - Immunoflourescence
IFNG - Interferon-γ
IHC - Immunohistochemistry
IL-2 / -7 / -15 - Interleukin-2 / -7 / -15
L1CAM - L1 cell adhesion molecule
LNP lipid nanoparticle
LTR - Long terminal repeat
MACS - Magnetic-activated cell sorting
MFI - Mean fluorescence intensity
MIT - Massachusetts Institute of Technology (used in CRISPR specificity scoring)
NGG - Protospacer adjacent motif recognized by SpCas9
NK - Natural killer (cell)
NSG - NOD-scid IL2Rγnull (immunodeficient mouse model)
PAM - Protospacer adjacent motif
PBS - Phosphate-buffered saline
PBMC - Peripheral blood mononuclear cell
PCR - Polymerase chain reaction
PE - Phycoerythrin
Pen/Strep - Penicillin/Streptomycin
qPCR - Quantitative polymerase chain reaction
Q8 - Custom reporter epitope tag containing CD34 epitope and CD8 transmembrane domain
REP - Rapid expansion protocol
RNP - Ribonucleoprotein
RPMI - Roswell Park Memorial Institute (medium)
SD - Standard deviation
sgRNA - Single-guide RNA
SNV - Single nucleotide variant
SORT - Selective organ targeting
sPA - Synthetic poly(A) sequence
TAE - Tris-acetate-EDTA (buffer for electrophoresis)
TME - Tumor microenvironment
TRAC - T cell receptor alpha constant
UV - Ultraviolet
VEGF - Vascular endothelial growth factor
WES - Whole-exome sequencing
WGS - Whole-genome sequencing

Supplementary Notes

Supplementary Note 1
The increased frequency of novel PAM sites in neuroblastoma cell lines was associated with a higher prevalence of mutations toward cytosine (C) or guanine (G), as observed in these datasets **(Supplementary Fig. 2c).**

Supplementary Note 2
The FCGBP gene harbored five or more novel PAMs across all terminate NB cell lines, yet it did not exhibit a similar prominence in patient samples. Notably, there was no overlap between the top 10 genes with the highest novel PAM counts in cell lines and those in patient-derived data **(Fig. 1E)**. In patient exome data, DLG2 (Chr. 11) emerged as the gene with the highest prevalence of at least one novel PAM site, identified in 13 out of 54 patients **(Supplementary Fig. 1f)**.

Supplementary Note 3
To further investigate discrepancies between cell lines and patient data, we applied CancerPAM to publicly available whole-exome sequencing (WES) datasets from 48 neuroblastoma cell lines (including eight from our dataset) available on the DepMap portal. In DepMap 23Q4, a median of 25 novel PAMs were identified from 155 SNVs (19% [95% CI: 17-22%]), a value more consistent with patient-derived data compared to terminate NB cell line data **(Supplementary Fig. 2a)**. However, cross-comparison of different DepMap versions (20Q4, 22Q2 and 23Q4) for the same 48 cell lines revealed inconsistencies. DepMap 23Q4 reported fewer SNVs and novel PAMs, with only partial overlap between versions **(Supplementary Fig. 2b,c)**. Additionally, DepMap 23Q4 exhibited a significantly lower percentage of SNVs relative to the terminate NB dataset (Supplementary Fig. 2d). Surprisingly, the concordance of SNVs between DepMap datasets and terminate NB was low, with only 46% of SNVs from DepMap 23Q4 and 45% from DepMap 20Q4/22Q2 overlapping with terminate NB, despite terminate NB reporting nearly 10-fold more SNVs than DepMap 23Q4 (median 1,470 vs. 155 SNVs) **(Supplementary Fig. 2e,g, S3)**. As expected, a gene-level analysis of chromosomes 1 and 19 revealed a higher proportion of genes with at least two novel PAM sites in terminate NB, reinforcing the increased mutation burden in these datasets. Only three genes on Chr. 1 and none on Chr. 19 harbored at least two novel PAMs shared across both datasets **(Supplementary Fig. 4).**

Supplementary Note 4
When analyzing the correlation of other features besides CRISPR specificity scores with the total PAM count per patient, a similar, albeit weaker, effect was observed for CRISPR efficiency scores, while no significant correlation was detected for copy number or gene expression. However, the copy number of top-ranked PAM sites was positively correlated with the total number of PAMs detected per patient, suggesting a potential structural genomic influence on PAM site availability **(Supplementary Fig. 7).**

Supplementary Note 5
To explore possible confounding factors, we examined the correlation between PAM feature characteristics and MYCN amplification status. Analysis of DepMap 23Q4 data indicated a slightly higher proportion of novel PAM sites located in non-essential genes in MYCN non-amplified cell lines. However, the observed effect size was marginal (median difference of 1.1%), and the findings could not be reproduced in the eight terminate NB cell lines with available dependency data, indicating that MYCN amplification status is unlikely to be a major determinant of PAM site distribution **(Supplementary Fig. 8).**

Supplementary Note 6
Preliminary CRISPR cutting efficiency experiments demonstrated relevant cutting activity (>5%) at all loci, except for *SCAF11*, *SH3BP1* and *SNX18*. The measured relative cutting frequency exhibited only a weak positive correlation with the Doench score and showed no correlation with the Moreno score. However, the strongest correlation - albeit not statistically significant - was observed for higher copy number regions **(Supplementary Fig. 9a, b).**

Supplementary Note 7
Optimization of HDRT constructs for knock-in revealed that a custom-designed EF1a-derived shortened promoter in combination with a Q8 (CD8 transmembrane + CD34 Qbend epitope) reporter exhibited the highest knock-in efficiency. In contrast, HDRTs incorporating a larger hCMV promoter or fluorophores such as RFP and GFP showed reduced efficiency, likely due to their increased genetic payload. We also tested a multicistronic vector encoding two cytokines, which demonstrated stable knock-in at the AAVS1 locus but with significantly lower efficiency.

Supplementary Note 8
We also evaluated a combinatorial knock-in strategy using two HDRTs (differing homology arms) and two gRNAs (targeting distinct tumor-specific loci) simultaneously, without increasing total DNA or gRNA dosage. This approach led to increased reporter expression after 28 days **(Supplementary Fig. 10b).** However, despite its efficacy, this strategy was not pursued in subsequent experiments due to the potential risk of chromosomal translocation. Lastly, we observed significantly lower knock-in rates for IFNG, particularly in SK-N-BE2c, suggesting locus- or transgene-specific effects on knock-in efficiency **(Fig. 3d, Supplementary Fig. 10c).**

Supplementary Note 9
Additional Sanger sequencing of Out/In PCR products provided definitive proof of on-target knock-ins **(Supplementary Fig. 11a).** Further analysis using Out/Out preferential amplification followed by Sanger sequencing and PAM site frequency determination confirmed the loss of the novel PAM site post-knock-in on a quantitative level **(Supplementary Fig. 11c)**. Analysis of weak-positive dPCR signals indicated reduced performance and sensitivity of the Out/In dPCR assay used for investigating AAVS1 knock-ins, which explains the discrepancy between higher knock-in rates observed in flow cytometry compared to dPCR for AAVS1 knock-ins **(Supplementary Fig. 12c).**

Supplementary Note 10
Unspecific knock-in, was assumedly not entirely due to CRISPR/Cas9-related effects, as transfection of high doses of Q8-HDRT alone also led to an increase in Q8+ positivity after 21 days - although this was not statistically significant- when compared to Q8+ positivity of control cells transfected with a GFP-HDRT. After subtracting the mean Q8+ count, only *IGSF9B* and *RPLP0* remained as gRNAs significantly associated with CRISPR-mediated unspecific knock-in (**Fig. 4e, Supplementary Fig. 13c**). The observed trend for inverse correlation between unspecific knock-in rates and CancerPAM ranking scores (**Fig.4g,h**) persisted after subtracting mean DNA-only transfection Q8+ cell counts **(Supplementary Fig. 13d**).

Supplementary Note 11
We did not observe any growth advantage or disadvantage in the enriched transgenic tumor cell lines, regardless of cell line, target locus, or cytokine combination **(Fig. 5C, Supplementary Fig. 14a,b).**

Supplementary Note 12
During in vitro CAR T cell SK-N-AS tumor co-culture, we did not observe any differences in the expression of activation markers (CD25, CD137) or exhaustion markers (LAG3, TIM3, PD1), as measured via flow cytometry, among the different cytokine-expressing tumor cell lines **(Supplementary Fig. 16b,c)**. Interestingly, co-culture with tumor cells resulted in elevated CXCL10 and CXCL11 levels in all CAR T cell plus unmodified or transgenic tumors, except when co-cultured with IFNG-expressing tumors. Regarding IFNG release, we observed that CAR T cell-mediated IFNG secretion was only a fraction of the levels produced by IFNG-transgenic tumors. CAR T cell IL-2 secretion remained unaffected by additional cytokine secretion from tumor cells **(Supplementary Fig. 17).**

Supplementary Note 13
After confirming similar engraftment across all treatment groups, comparison of day 1 BLI data post-CAR T cell injection in the lung showed no significant differences between tumor groups **(Supplementary Figs. 24a,b).**

Supplementary Note 14

Serum cytokine level analysis via ELISA confirmed CXCL10 expression in mice harboring transgene-positive tumors, both pre- and post-treatment **(Supplementary Fig. 21a, S24f).** Additionally, post-mortem immunofluorescence analysis of tumor sections confirmed persistent L1CAM positivity and low-abundance CD3+ T cells within tumors **(Supplementary Fig. 25).**

Supplementary Note 15

In contrast to the xenograft-only NOG model, longitudinal BLI in humanized mice did not yield quantifiable tumor signals following CAR T-cell infusion **(Supplementary Fig. 33b,c).** Validation experiments using one HuNOG mouse (>120 days post-engraftment) and two NOG control mice without tumors, imaged one hour after CAR T-cell injection, confirmed functional luciferase activity and normal pulmonary CAR T-cell distribution, consistent with in vitro luciferase activity testing **(Supplementary Fig. 33a,d)**, thereby ruling out technical failure. Flow cytometry verified CAR T-cell persistence in blood and tumors at endpoint **(Fig. 7h,i)**, including in animals treated with autologous CAR T cells (6 out of 19), excluding early immunologic rejection as the cause of absent BLI signal. These six of nineteen mice had received autologous CAR T-cell products generated from T cells isolated from blood and spleen of animals engrafted with the same human $CD34^{+}$ stem cell pool. We therefore hypothesize that the high density of engrafted human immune cells within the HuNOG microenvironment may have competitively limited CAR T-cell expansion and photon emission below the detection threshold.

Supplementary Methods

Genomic DNA Sample Isolation

Genomic DNA was extracted from cultured cells using the QIAamp DNA Mini Kit (QIAGEN) following the manufacturer's guidelines with minor optimizations. Briefly, up to 5 × 10^6 cells were lysed using Proteinase K and buffer AL at 56°C, followed by ethanol precipitation. The lysate was passed through a QIAamp Mini Spin Column via centrifugation to bind DNA, followed by sequential washes with AW1 and AW2 buffers to remove contaminants. Purified DNA was eluted in Buffer AE and stored at -20°C until further use. DNA concentration and purity were assessed using a NanoDrop 2000 spectrophotometer (Thermo Fisher Scientific) at 260 nm absorbance, ensuring concentrations ≥10 ng/µL for downstream applications.

Standard PCR

PCR was performed using KAPA polymerase (Roche) to amplify DNA sequences for homology-directed repair templates (HDRTs), target sequencing and qualitative knock-in analysis. Primers were custom-designed using SnapGene (GSL Biotech LLC) and Primer3Plus (https://www.primer3plus.com/) and Primer-BLAST (NCBI). PCR reactions were carried out using a C1000 Touch Thermal Cycler (Bio-Rad) under optimized cycling conditions. For sequence analysis, amplified fragments purified using the QIAquick PCR Purification Kit (QIAGEN) and sequenced by LGC Genomics. PCR product specificity and efficiency were confirmed by agarose (1-2%) gel-electrophoresis using TAE buffer-based gels, with GelRed (Sigma-Aldrich) staining for visualization. A detailed list of primers and oligonucleotides used in the study is provided in **Supplementary Table 4**.

Cell Lines and Cell Culture

Neuroblastoma cell lines SK-N-BE(2)c and SK-N-AS cells were obtained from Prof. Michael Claus V Jensen at Seattle Children's Hospital. HEK293T cells (ATCC CRL-3216) and neuroblastoma cell lines were cultured in DMEM high glucose medium (Gibco) with 10% heat-inactivated fetal calf serum (FCS) and 1% Penicillin/Streptomycin (Pen/Strep, Gibco). Human Umbilical Vein Endothelial Cells (HUVECs, ATCC CRL-1730) were cultured in Endothelial Cell Growth Medium 2 (PromoCell, Cat# C-22111/39211), supplemented with EGF, IGF, Vascular Endothelial Growth Factor (VEGF), FGF2, 2% Fetal Bovine Serum (FBS) and 1% Pen/Strep. All cell lines were maintained at 37°C with 5% CO2. Lymphocytes were isolated from fresh primary adult blood cells from anonymous healthy human donors using EasyStep isolation kits for $CD3^+$, $CD4^+$ or $CD8^+$ T cells (StemCell Technologies). Isolated T cells were cultured at an initial density of 10^6 cells per ml in X-VIVO 15 medium (Lonza) supplemented with human serum (5%, Gemini), penicillin-streptomycin (1%, Gibco), interleukin (IL-7; 5 ng/ml, Miltenyi) and IL-15 (5 ng/ml, Miltenyi). After isolation, cells were stimulated for 2 days with anti-human CD3/CD28 magnetic Dynabeads (Thermo Fisher Scientific) using a 1:1 bead-to-cell ratio.

Evaluation of Cell Proliferation and Viability Following CRISPR/Cas9-Mediated Gene Integration

Genome-edited SK-N-BE2c and SK-N-AS neuroblastoma cell lines, along with their wild-type counterparts, were used for proliferation and viability assessments. Cell proliferation was monitored over seven days using the IncuCyte® Live-Cell Analysis System (Sartorius). Cells were seeded in 96-well flat-bottom plates at 1 × 10^4 cells per well in biological duplicates and technical triplicates. Phase-contrast imaging was performed at regular intervals to generate quantitative growth curves. Viability was assessed using IncuCyte® Cytotox Green reagent (Sartorius, catalog #4633) at a final concentration of 250 nM, added during seeding. Green fluorescence was monitored using IncuCyte® software to quantify non-viable cells with compromised membrane integrity.

Cytokine Quantification via ELISA

Cytokine levels were quantified using enzyme-linked immunosorbent assays (ELISA) following the manufacturer's protocols. CXCL10 and CXCL11 concentrations were determined using the Human CXCL10/IP-10 and CXCL11/I-TAC DuoSet® ELISA kits (R&D Systems, Cat# DY266 and DY392), while IL-2 and IFNG levels were measured using the BD OptEIA™ ELISA sets (BD Biosciences). 96-well microplates (NUNC) were coated overnight at 4°C with the respective cytokine-specific capture antibodies. After washing, plates were blocked with 10% fetal calf serum (FCS) for IL-2 and

IFNG ELISAs or 1% bovine serum albumin (BSA) for CXCL10 and CXCL11 ELISAs. Frozen samples were thawed and analyzed at multiple dilution factors to ensure accurate quantification: 1:10, 1:100, or undiluted. Detection was performed using biotinylated secondary antibodies, streptavidin-HRP and TMB substrate, with signal development for 20 min at room temperature in the dark. The reaction was stopped with 2N H_2SO_4 (50 µL/well). Absorbance was measured at 450 nm (reference: 570 nm) using an Epoch™ microplate spectrophotometer (Gene5 software). Standard curves were generated using four-parameter linear regression in Microsoft Excel.

Flow Cytometry and Fluorescence-Activated Cell Sorting (FACS)

Flow cytometry was used to analyze transgene expression, cell surface markers and viability, while fluorescence-activated cell sorting (FACS) was performed to enrich successfully modified cells. All flow cytometric analyses were conducted on BD FAC LSRFortessa X-20 (Lasers for Detection: 405 nm (violet), 488 nm (blue), 561 nm (yellow-green), 640 nm (red)), with sorting performed using a BD FACSAria III. Data acquisition and analysis were performed using FlowJo v10. A comprehensive list of all antibodies, fluorochromes is provided in **Supplementary Table 6** and detailed gating strategies are illustrated in **Supplementary Figures S10, S19 and S21**. Cells were collected, washed twice with PBS and resuspended in 100 µL staining solution containing the respective fluorochrome-conjugated antibodies. For CD34-epitope detection and sorting, cells were labeled with PE-conjugated anti-CD34 monoclonal antibody (clone QBEND/10, Invitrogen, dilution 1:10) and incubated at 4°C for 30 min in the dark. For samples requiring CAR expression analysis, cells were pre-stained with anti-mouse F(ab′)2 and blocked with mouse serum (MilliporeSigma) before antibody labeling. FACS was used to enrich CD34-positive genome-edited cells, ensuring a homogeneous population. Cells were harvested, stained for CD34 expression and sorted using BD FACSAria III. The sorted fraction was collected in FBS-coated tubes, resuspended in culture medium and expanded for further analyses. Fluorescence signals were compensated using single-stained CD8+ T cells.

Boyden chamber and Clearview trans well migration assays

The migration potential of L1CAM-specific CAR T cells was assessed using a 24-well Boyden chamber transwell system with 8 µm pore size polycarbonate membranes. Inserts were pre-coated with 0.5% bovine serum albumin (BSA) in PBS for 1 hour at room temperature. CAR T cells were resuspended at 5×10^6 cells/mL at day 12-15 after expansion initiation seeded into the upper transwell chamber (100 µL per well). The lower chamber contained 500 µL of conditioned medium from cytokine-expressing neuroblastoma cells or control medium. Migration was monitored for 4 hours using the IncuCyte S3 live-cell imaging system and data were analyzed using the IncuCyte S3 software. Data were normalized to initial seeding density and analyzed using GraphPad Prism with migration parameters compared via Gompertz-Laird model fitting and statistical tests. The ClearView Transwell Migration Assay was performed using the IncuCyte ClearView Cell Migration Plate in a 96-well format with a pore size of 8 µm. The membrane surface was coated with 1% BSA solution before being incubated with Protein G (20 µg/mL, Thermo Fisher, #101200) for 1 hour at 37°C, followed by an additional 40 µL wash with Protein G solution. The membrane was then coated with ICAM-1 (5 µg/mL, Sino Biological, #10346-H03H) for 2 hours at 37°C before both sides were blocked with PBS + 1% BSA for 30 min at room temperature. CAR T cells (50,000 per well) were seeded at day 12-15 after expansion initiation in 60 µL RPMI + 0.5% FCS and allowed to attach for 45-60 min before the addition of 200 µL chemoattractant solution prepared in RPMI + 0.5% FCS to the lower chamber.

Cytotoxicity Assays

To assess the cytotoxic activity of L1CAM targeting CAR T cells against tumor cells, real-time imaging was performed using the IncuCyte® Live-Cell Analysis System (Sartorius). SK-N-AS transgenic or control tumor cells were seeded in 48-well plates at a density of 0.05×10^6 cells per well and cultured in RPMI + 10% FBS + 1% P/S for 16 hours prior to co-culture. To enable live-cell imaging, tumor cells were fluorescently labeled using Vybrant DiO Cell-Labeling Solution (Thermo Fisher) at 1:500 dilution for membrane staining and IncuCyte Nuclight Rapid Red Dye (Sartorius) at 1:4000 dilution for nuclear staining. Following a 30-minute incubation at room temperature, tumor cells were washed, resuspended in fresh culture medium and transferred to the IncuCyte chamber. After tumor cell adherence and spreading, CAR T cells were introduced at an effector-to-target (E:T) ratio of 1:5 in a final volume of 400 µL per well.

CAR T cells were cultured in RPMI + 10% FBS + 1% P/S + 1% GlutaMAX. Live-cell imaging was conducted every 2 hours over a 96-hour period using a 20× objective, capturing phase-contrast, green and red fluorescence images to monitor tumor cell viability. The number of viable tumor cells was determined based on nuclear staining intensity and cytotoxicity was quantified as the percentage of tumor cell loss over time relative to untreated controls. For experiment reproducibility, biological duplicates and technical triplicates were performed per condition. To account for donor variability, CAR T cells from at least two independent donors were tested, with repeated experiments performed on a separate day using the same donors.

Macrophage Polarization Assay

Human peripheral blood monocytes were isolated from healthy donor buffy coats by density gradient centrifugation and magnetic $CD14^+$ selection. For tumor cell-dependent polarization, 5×10^5 neuroblastoma cells were seeded in the lower chamber of 12-well transwell plates (0.4 µm pore size; Corning) in RPMI 1640 supplemented with 10% FCS, 1% GlutaMAX, and penicillin/streptomycin. Monocytes (5×105 per insert) were plated in the upper chamber and co-cultured for 6 days in the presence of M-CSF (50 ng/mL). Where indicated, tumor cells were engineered to express IFNG, or recombinant IFNG was added to selected wells. As controls, monocytes were polarized with M-CSF (50ng/mL), IL4 (20ng/mL) and IL10 (20ng/mL) for M2-like, GM-CSF (50ng/mL) and recombinant IFNG (100ng/mL) for M1-like, M-CSF (50ng/mL) plus IFNG (75ng/mL), or left untreated. Media were partially exchanged every 48-72 h with fresh RPMI containing the respective cytokines. On day 6, adherent macrophages were harvested and stained for flow cytometric analysis. Antibodies included CD14 (PerCP/Cy5.5), CD64 (PE), CD86 (FITC), HLA-DR (BV711), CD163 (APC), CD206 (BV421), and CSF-1R (PE/Cy7), with Fc receptor blocking (Human TruStain FcX, BioLegend) performed prior to staining. Data were acquired via flow cytometry (BD FACSCanto II) and analyzed using FlowJo software.

Generation of L1CAM knock-out SK-N-BE2c

SK-N-BE2c cells were transfected with a plasmid encoding Cas9 and a L1CAM-targeting guide RNA and a puromycin resistance gene. Specifically, 1×10^6 cells were seeded in a 6-well plate containing 3 mL of culture medium. The following day, the medium was replaced and cells were transfected using Effectene Transfection Reagent (QIAGEN) according to the manufacturer's protocol, utilizing 1 µg of plasmid DNA per transfection. On day two post-transfection, the medium was refreshed and on day three, selection commenced with 0.5 µg/mL puromycin. A control plasmid lacking the puromycin resistance gene was used as a negative control. By day ten, knockout efficiency was assessed via flow cytometry. Subsequently, to create a cell line with complete L1CAM knockout, single-cell clones were isolated and expanded.

Immunohistochemistry (IHC)

Immunohistochemistry was performed on paraffin-embedded tumor tissues. Tumors were fixed, dehydrated and embedded in paraffin before sectioning into 6 µm slices using a microtome. Sections were mounted on positively charged glass slides and subjected to deparaffinization in xylene (twice, 5 min each), followed by rehydration in a graded ethanol series (100%, 95%, 70% and 50% ethanol, 5 min each) and rinsing in distilled water. Antigen retrieval was performed in citrate buffer (10 mM tri-sodium citrate, 0.05% Tween-20, pH 6.0, Abcam ab93678) using a pressure cooker (2 min at 125°C, cooling to 90°C, then under running water for 10 min). After an additional 5 min incubation in distilled water, sections were washed in PBS (5 min). To prevent non-specific binding, blocking was performed using 3% normal serum (corresponding to the species of the secondary antibody) in TBS-T for 1 hour in a humidity chamber. For immunofluorescence staining, primary antibodies were applied in 3% serum in TBS-T and incubated overnight at 4°C. The following primary antibodies were used: rabbit anti-human/mouse CD3 (Abcam, ab16669, clone SP7, 1:100), mouse anti-human L1CAM (Invitrogen, MA1-46044, clone UJ127.11, 1:500). The next day, slides were washed five times in TBS-T before incubation with fluorescently labeled secondary antibodies in TBS-T and 3% serum for 1 hour at room temperature. The following secondary antibodies were used: donkey anti-rabbit Alexa 594 (Invitrogen, Lot# 15146896, 1:300), donkey anti-mouse Alexa 488 (Invitrogen, Lot# 15960296, 1:300), goat anti-rabbit Alexa 594 (Invitrogen, Lot# 10266352, 1:500), goat anti-mouse Alexa 488 (Invitrogen, Lot# 10256302, 1:500). Following secondary antibody incubation, slides were washed 3 times in TBS-T and counterstained with Hoechst (1:2000 in TBS-T, 2-5 min). After a final wash in distilled water (5 min), samples were mounted using Eukitt quick-hardening mounting medium (Sigma-

Aldrich, Lot# BCBP7505V). Immunofluorescence images were acquired using a fluorescence microscope (Olympus BX43) and analyzed with Olympus cellSens software.

Statistical estimation of tumor-specific CRISPR off-target risk

To estimate the probability of tumor-specific off-target events, we developed a Python-based tool that calculates the cumulative statistical risk of two classes of events: (1) rescue of gRNA off-target sites through somatic SNVs and (2) creation of novel PAM sites. These include cases where a gRNA designed for a novel PAM has a highly similar sequence elsewhere in the genome (≤4 mismatches), and a tumor-specific SNV either converts the mismatched site into a perfect or near-perfect match or generates an adjacent NGG PAM where none existed before. For each gRNA designed by CancerPAM, all potential genomic matches with up to four mismatches in the human reference genome (hg38) were considered. The probability that a tumor-specific SNV could convert such a mismatch into a perfect match was estimated as a function of the total number of somatic variants present in the tumor and the sequence similarity profile of the gRNA. We assumed a 1/3 probability that a random SNV corrects a mismatch and a 0.133 probability that an SNV in the PAM region generates an NGG site, with genome size set to 3.1×10^9 bp. This approach yields a probability value for the occurrence of tumor-specific off-targets without requiring variant-level overlay, enabling rapid bulk analysis. The analysis was applied to representative gRNA sequences identified by CancerPAM from neuroblastoma patient samples and correlated with CancerPAM percentile ranks of the respective novel PAM sites (Supplementary Figures).

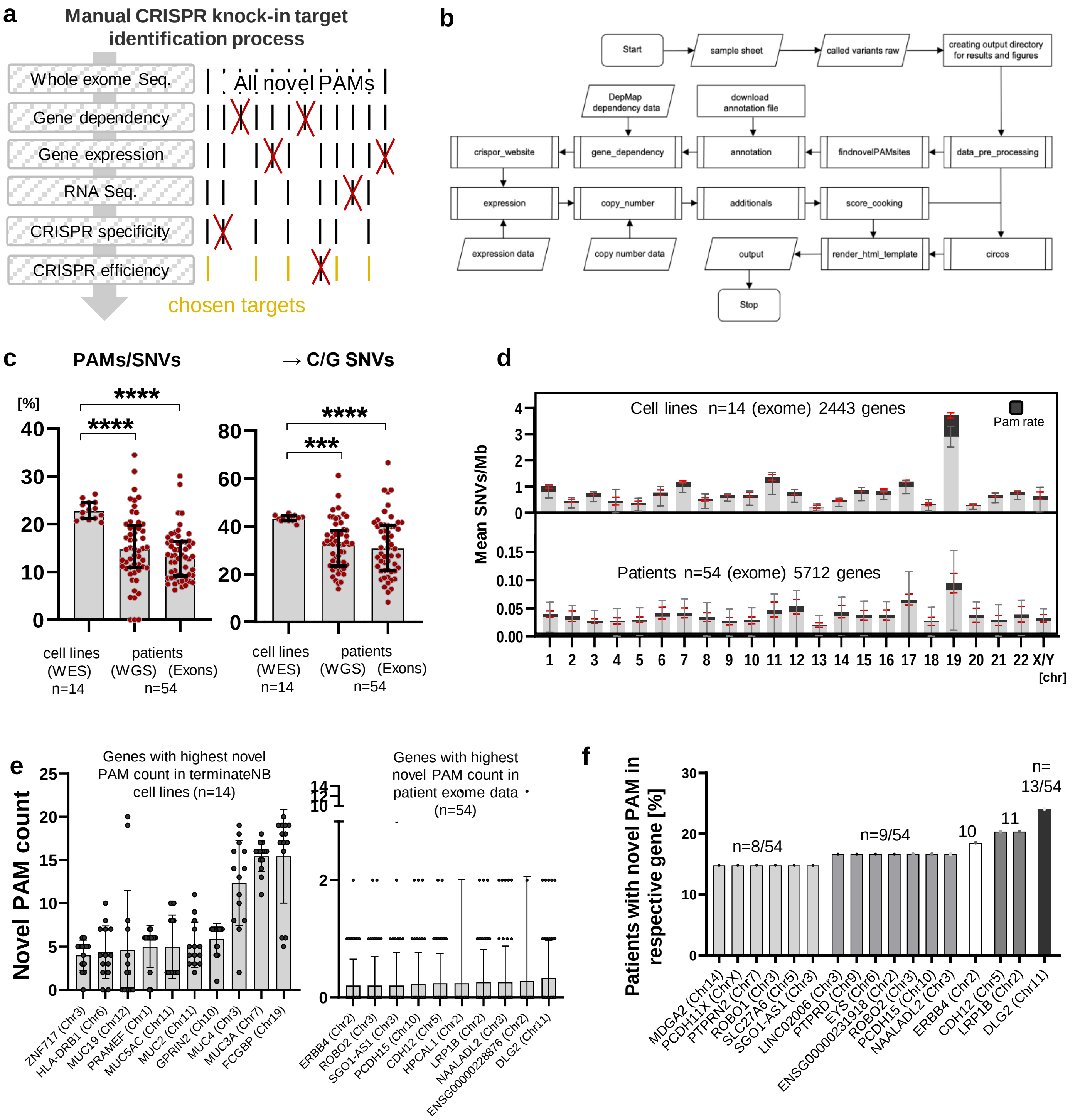

Supplementary Fig. 1. CancerPAM multi-omics-based automated pipeline. (a) Manual multi-step CRISPR knock-in target identification process preceding the CancerPAM pipeline, where novel PAM sites were excluded as potential targets if they fell below defined threshold values at each step. **(b)** Visualization of the CancerPAM pipeline data flow. **(c)** Comparison of the proportion of novel PAMs within all single nucleotide variants (SNVs) and mutations to C or G (→ C/G) in cell line versus patient data. **(d)** Chromosomal distribution of exonic mutations in neuroblastoma cell lines and patient samples(F-H) Proportion of patients with novel PAM counts in the same gene, focusing on the 16 genes most frequently recurring across patients. **(e)** Quantification of novel PAM counts in the top 10 genes with the highest novel PAM counts in cell line or patient data. **(f)** Proportion of patients with novel PAM counts in the same gene, focusing on the 16 genes most frequently recurring across patients. *n* represents individual patients. **Data presentation**: (c, d, e) Mean ± SD. Data points und *n* represent individual cell lines or patients. **Statistical analysis**: (c) Kruskal-Wallis with Dunn's post hoc test. *p* values: *<0.05, **<0.01, ***<0.001, ****<0.0001; *n.s.*: not significant. Source data and exact p values are provided as a Source Data file.

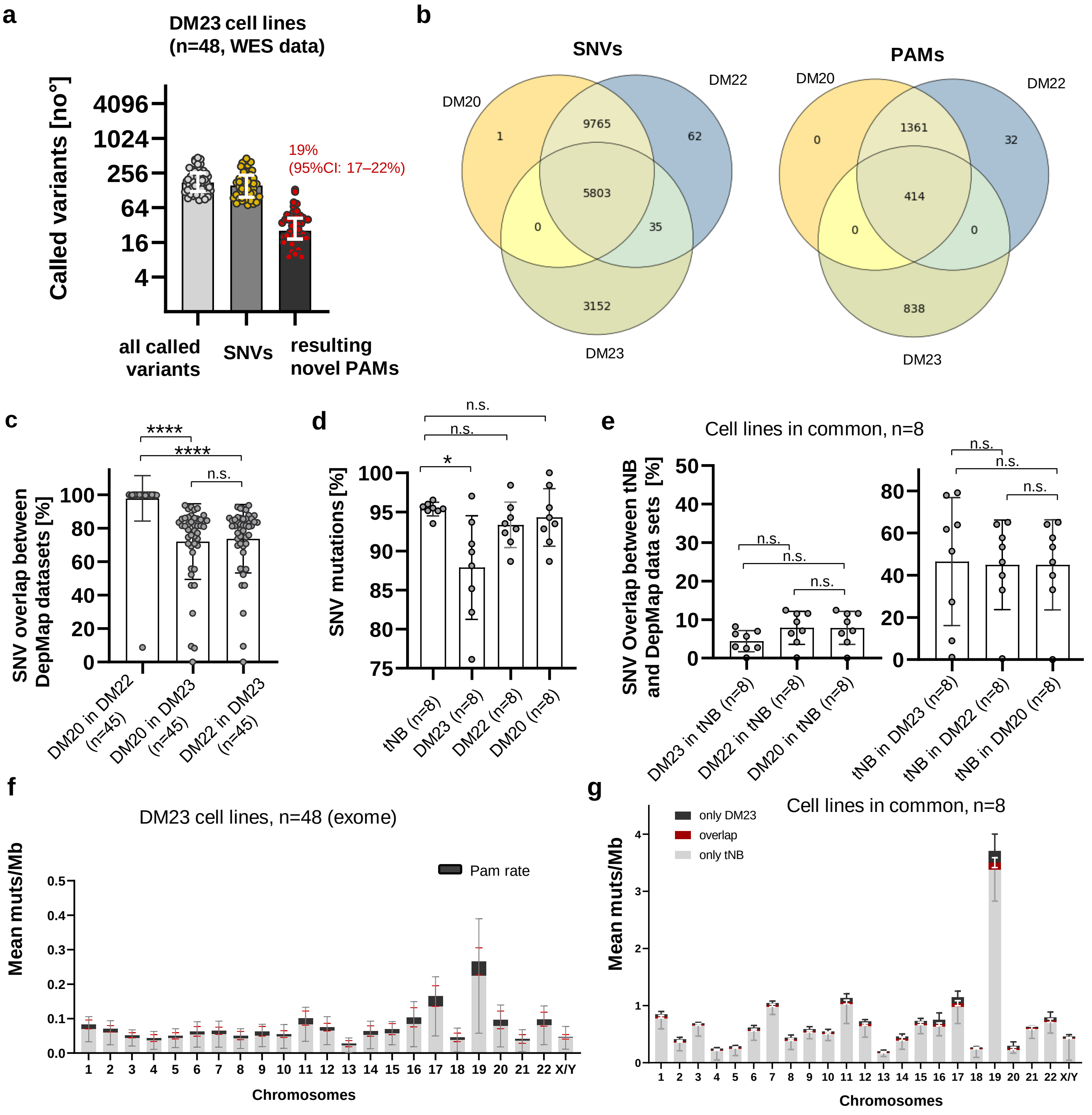

Supplementary Fig. 2. Dataset comparison between Terminate NB and DepMap. (a) Quantification of identified variants and tumor-specific PAM sites in neuroblastoma cell lines from the DepMap 23Q4 (DM23) dataset (WES data) identified using the CancerPAM pipeline. **(b)** Overlap of SNVs and novel PAMs identified in neuroblastoma cell lines across the three DepMap datasets: DepMap 20Q4 (DM20), DepMap 22Q2 (DM22), and DepMap 23Q4 (DM23). **(c)** Overlap of identified SNVs between DepMap datasets, where bars represent the percentage of SNVs in the first dataset that were also found in the second dataset. **(d)** Comparison of the percentage of identified SNVs between terminate NB (tNB) and DepMap datasets. **(e)** Overlap of identified SNVs between terminate NB and DepMap datasets for all cell lines present in all datasets, with bars showing the percentage of SNVs in the first dataset also identified in the second dataset. **(f)** Chromosomal distribution of exonic mutations in neuroblastoma cell lines from the DM23 dataset. **(g)** Overlap of identified SNVs and novel PAMs between terminate NB and the DM23 dataset per chromosome. **Data presentation**: (a,c-g) Mean ± SD. Data points und *n* represent individual cell lines. **Statistical analysis**: (c-e) Kruskal-Wallis with Dunn's post hoc test; *p* values: *<0.05, **<0.01, ***<0.001, ****<0.0001; *n.s.*: not significant. Source data and exact p values are provided as a Source Data file.

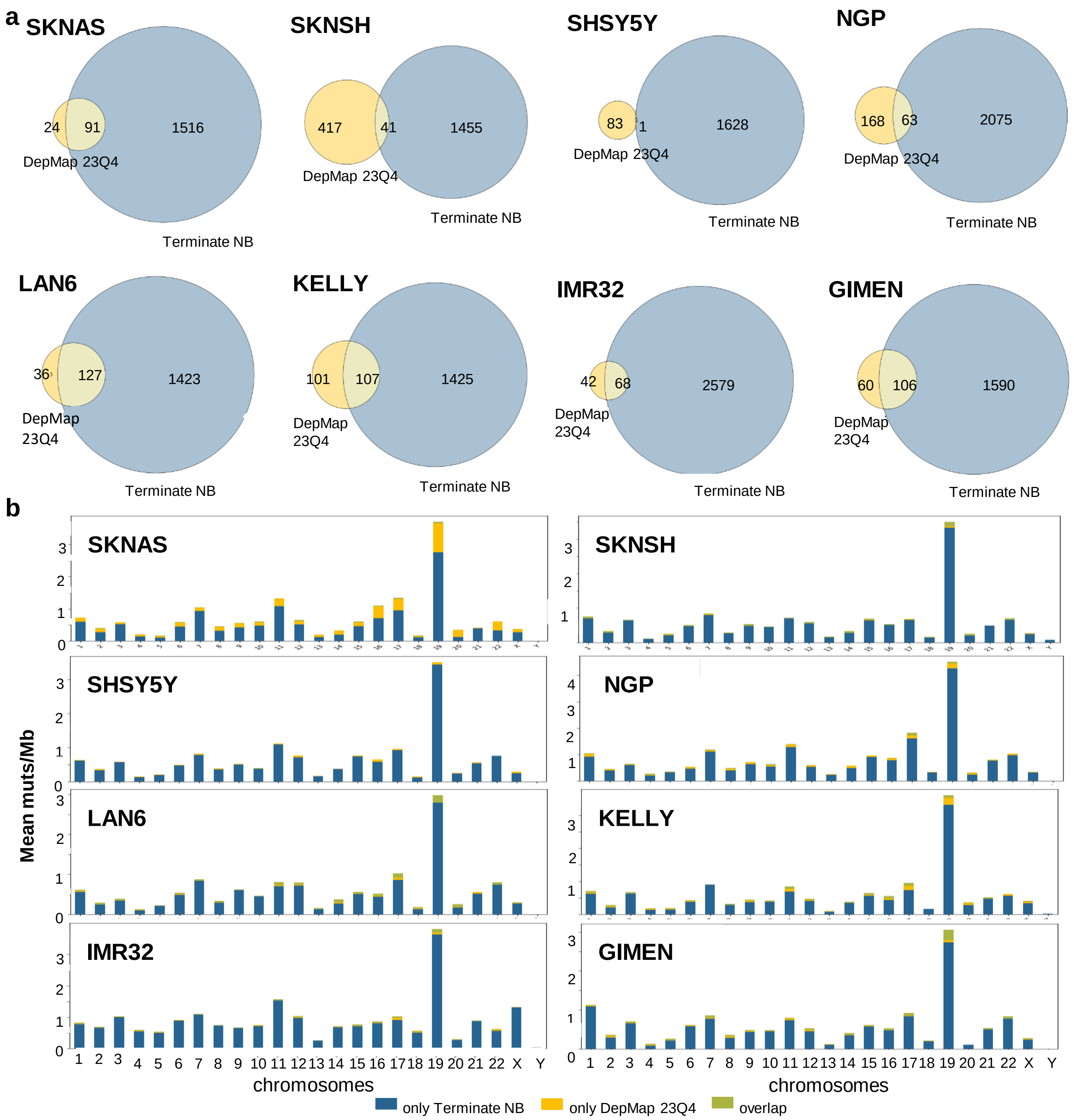

Supplementary Fig. 3. Cell line data comparison between Terminate NB and DepMap data sets. (a) Overlap of SNVs identified in neuroblastoma cell lines across the terminate NB and DepMap 23Q4 data sets for all cell lines included in both data sets (*n*=8). **(b)** Overlap of identified SNVs and novel PAMs between terminate NB and the DepMap 23Q4 dataset per chromosome for all cell lines included in both data sets (*n*=8). *n* represents individual cell lines. Source data are provided as a Source Data file.

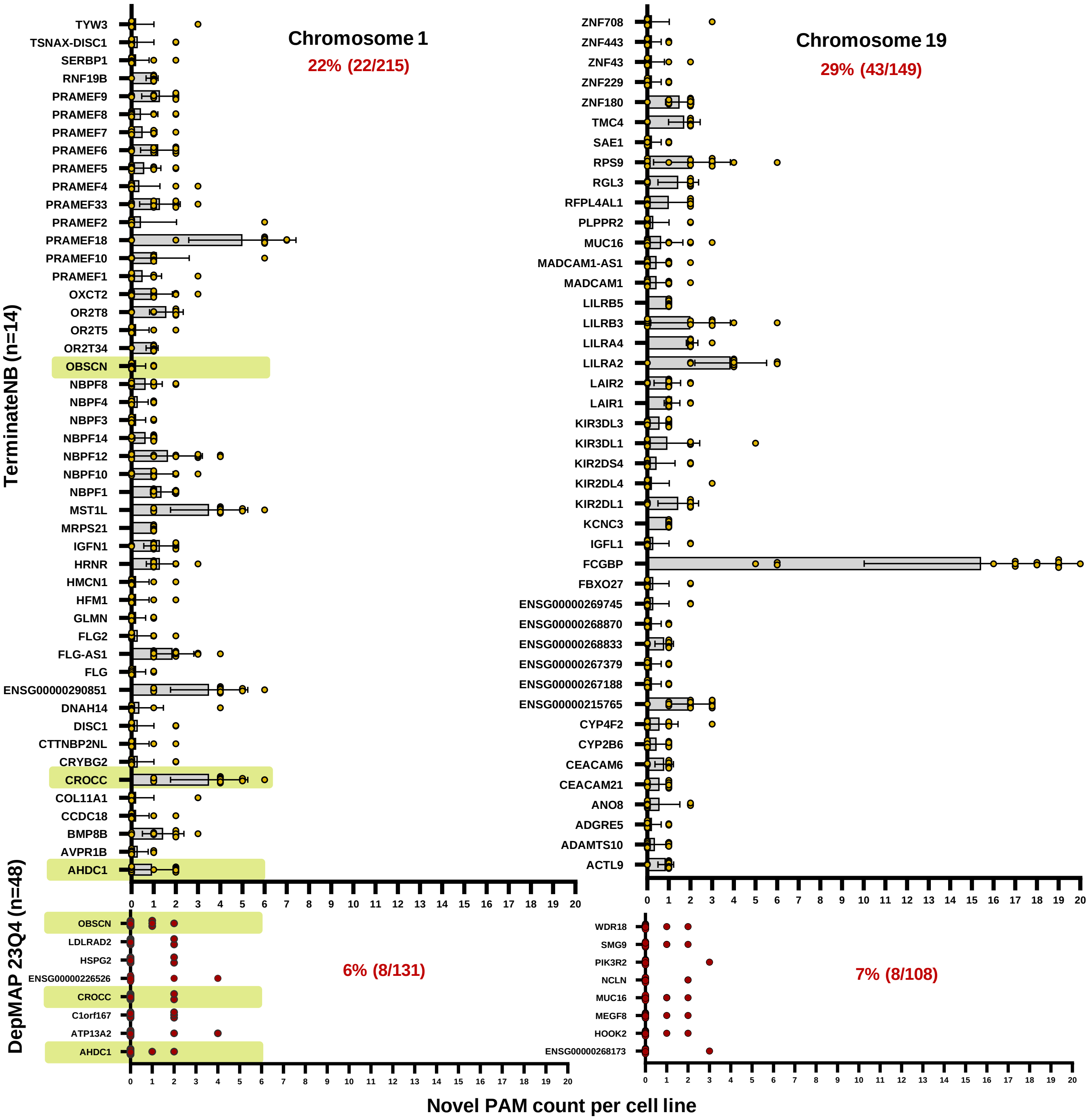

Supplementary Fig. 4. Chromosomal novel PAM distribution. Visualization of all genes with at least two novel PAMs on Chromosomes 1 and 19 found in at least one cell line from the terminate NB cell line dataset (*n*=14 cell lines) and the DepMap 23Q4 dataset (*n*=48 cell lines). In the terminate NB dataset, 22% (22/215) and 29% (43/149) of the genes on Chromosomes 1 and 19, respectively, with novel PAMs contained at least two novel PAMs in at least one analyzed cell line. For the DepMap 23Q4 dataset, this proportion was 6% (8/131) and 7% (8/108) for Chromosomes 1 and 19, respectively. Green bars indicate genes with at least two novel PAMs in at least one cell line that were shared between both datasets. **Data presentation**: Mean ± SD. Data points und *n* represent individual cell lines. Source are provided as a Source Data file.

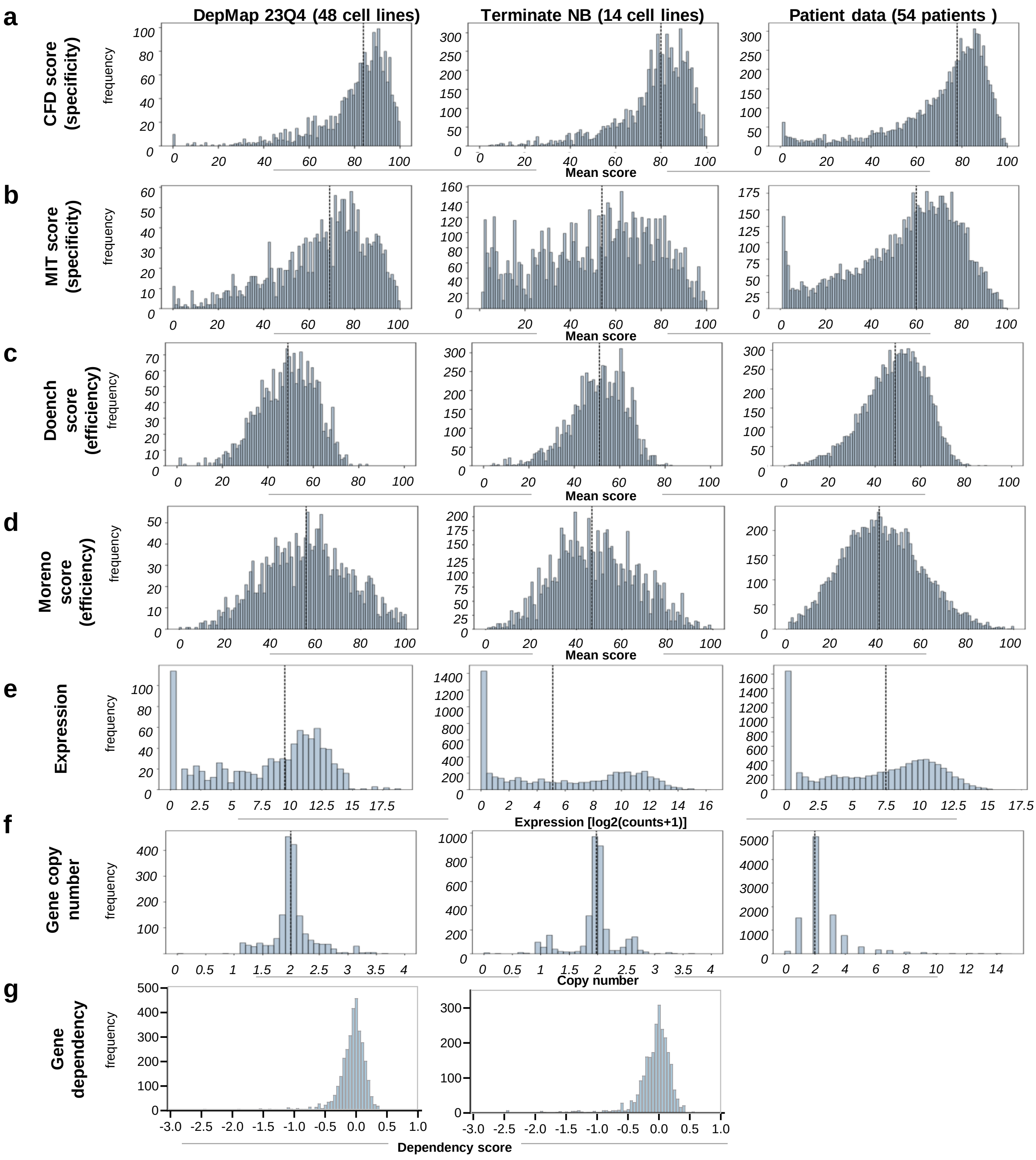

Supplementary Fig. 5. Novel PAM feature characteristics distribution. (a-g) Frequency distribution of values for various features annotated to the identified novel PAM sites in the DepMap 23Q4, terminate NB, and patient datasets. The DepMap 23Q4 dataset includes data from 48 cell lines, terminate NB includes 14 cell lines, and the patient dataset consists of 54 patients. Analyzed features include the CRISPR specificity scores CFD (**a**) and MIT (**b**), the CRISPR efficiency scores Doench (**c**) and Moreno (**d**), as well as gene expression levels **(e)**, copy number **(f)** and Gene dependency scores **(g)** associated with novel PAM sites. Source data are provided as a Source Data file.

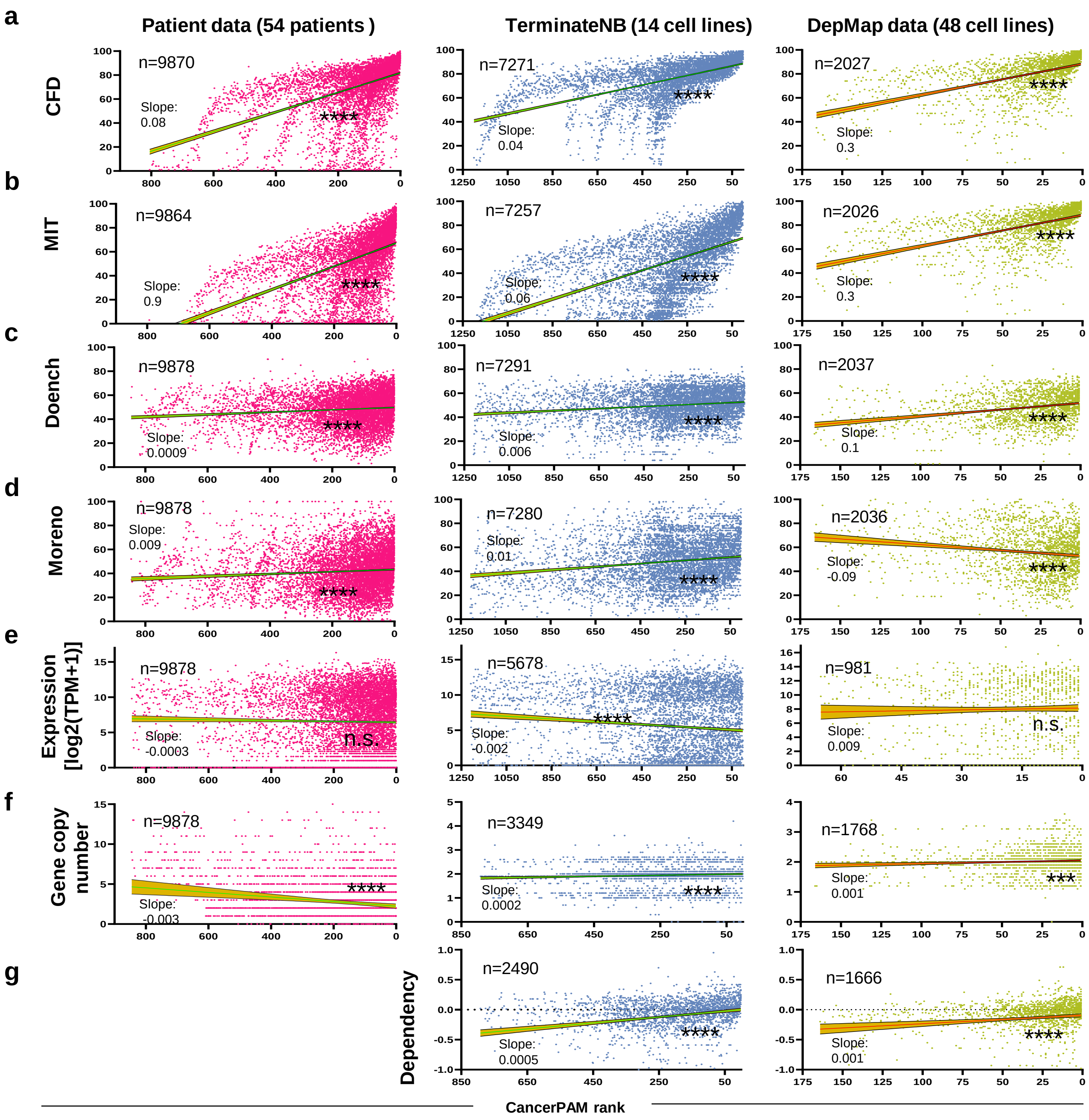

Supplementary Fig. 6. CancerPAM ranking validation. (a-g) Correlation analysis of novel PAM feature values against the CancerPAM rank of the identified novel PAM sites. Analyzed features include the CRISPR specificity scores CFD (**a**) and MIT (**b**), the CRISPR efficiency scores Doench (**c**) and Moreno (**d**), gene expression levels (**e**), copy number (**f**), and DepMap gene dependency (**g**, not available for patient data) from genes associated with the novel PAM sites. **Data presentation**: Dots and *n* represent individual PAM sites. **Statistical analysis**: Linear regression for curve fitting and slope calculation with error bands representing the 95 % confidence interval, followed by two-sided Spearman correlation analysis. *p* values: *<0.05, **<0.01, ***<0.001, ***<0.0001; *n.s.*: not significant. Source data and exact p values are provided as a Source Data file.

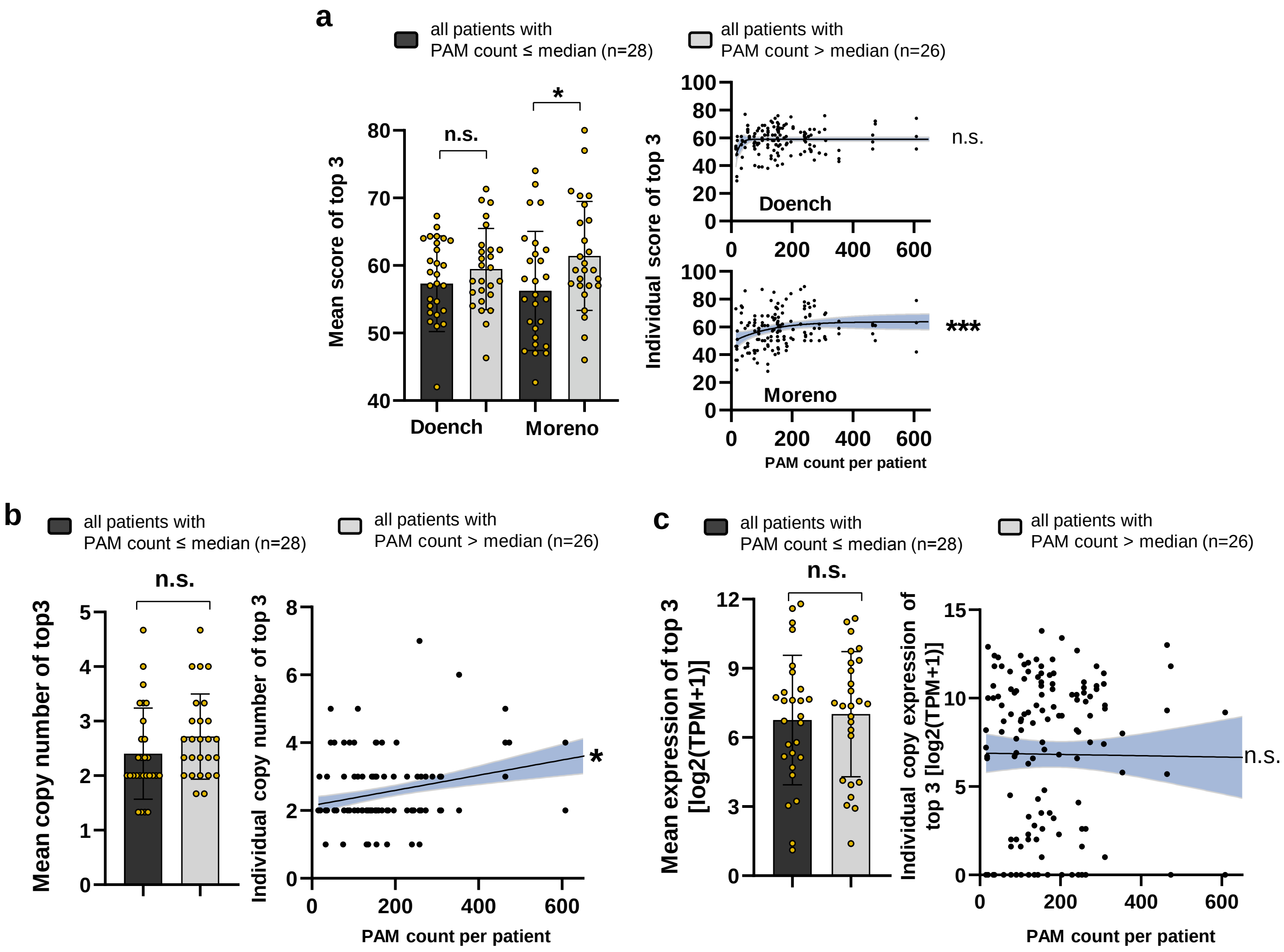

Supplementary Fig. 7. Feature correlation with PAM count. (a-c) Bar graphs comparing patients with a high (>median; *n* = 26) versus low (≤median; *n* = 28) PAM count in terms of the top three ranked PAM mean Doench and Moreno efficiency scores (a), copy number (b), or expression levels (c) X/Y scatter plots depict the correlation of individual scores for the top three ranked novel PAM sites across patients (*n* = 162) against total tumor-specific PAM counts per patient. **Data presentation:** (a-c) Mean ± SD. Data points and *n* represent individual patients. **Statistical tests:** (a-c) Bar graphs: Kruskal-Wallis with Dunn's post hoc test; X/Y scatter plots: logistic and linear regression for curve fitting with error bands representing the 95 % confidence interval, followed by two-sided Spearman correlation analysis. *p* values: *<0.05, **<0.01, ***<0.001, ****<0.0001; *n.s.:* not significant. Source data and exact p values are provided as a Source Data file.

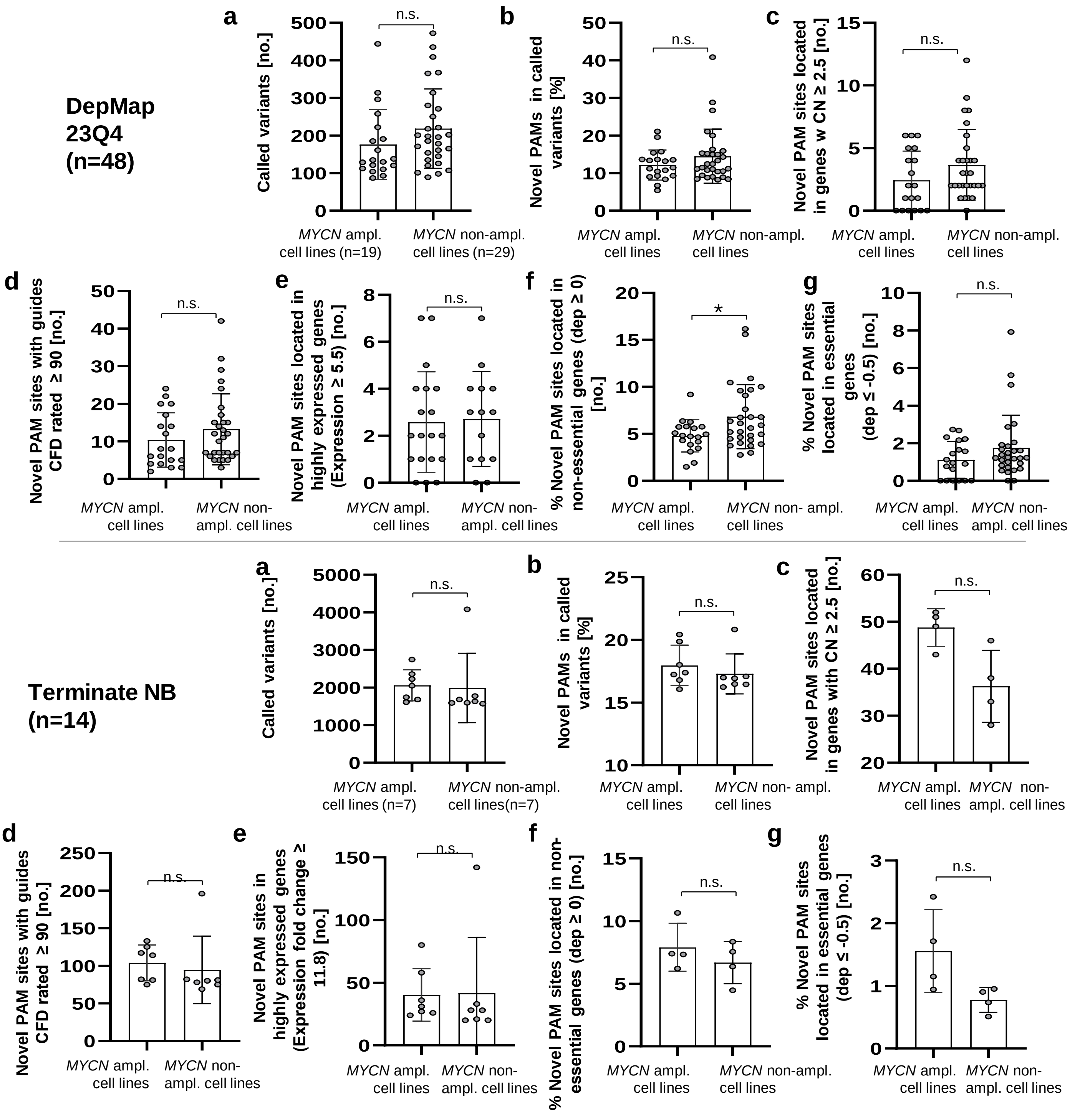

Supplementary Fig. 8. Influence of MYCN amplification status on novel PAM counts and features. (a-g) Group comparisons of novel PAM counts and feature values between MYCN-amplified (DepMap n=19, Terminate NB n=7)) and non-amplified cell lines (DepMap n=29, Terminate NB n=7) for the Terminate NB and DepMap 23Q4 datasets. Comparisons include the number of called variants per cell line. **(a)**, the percentage of novel PAMs among all called variants **(b)**, the number of novel PAMs located in genes with a copy number (CN) ≥ 2.5 **(c)**, the number of novel PAM sites with guides showing a CFD score ≥ 90 **(d)**, the number of novel PAM sites located in highly expressed genes with log2(counts+1) ≥ 5.5 **(e)**, the number of novel PAM sites located in non-essential genes with gene dependency (dep) values ≥ 0 **(f)**, and the number of novel PAM sites located in essential genes with gene dependency (dep) values ≤ -0.5 **(g)**. **Data presentation**: (a-g) Mean ± SD. Data points and *n* represent individual cell lines. **Statistical analysis:** (a-g): two-sided Mann-Whitney test. *p* values: *<0.05, **<0.01, ***<0.001, ***<0.0001; *n.s.*: not significant. Source data and exact p values are provided as a Source Data file.

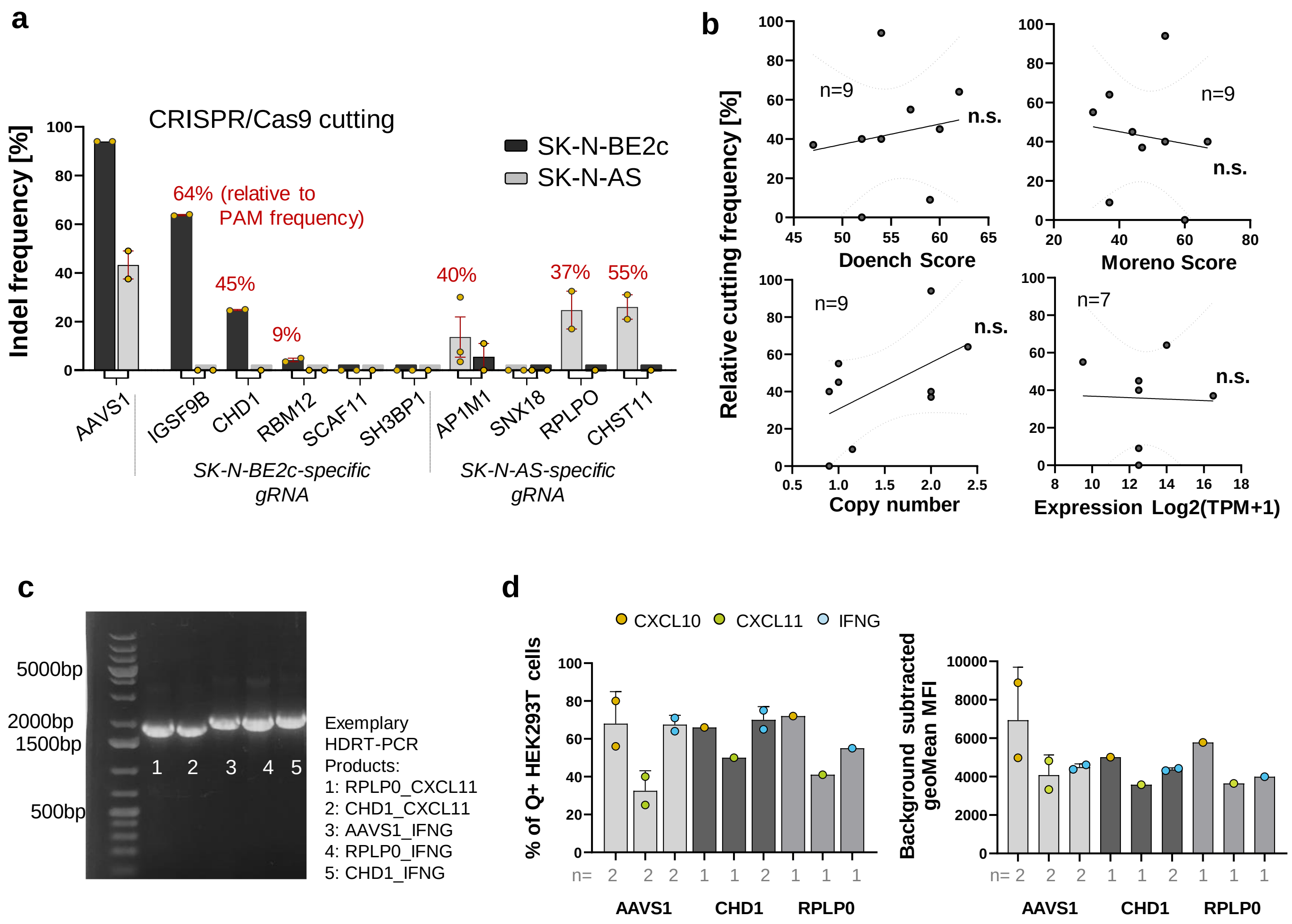

Supplementary Fig. 9. In vitro CRISPR cutting and transgene vector production and testing. (a) CRISPR/Cas9 cutting efficiency at the chosen target sites and the AAVS1 control locus, represented by indel frequency determined via Sanger sequencing and Synthego ICE analysis after Cas9/gRNA electroporation (n=2 biological replicates per locus and cell line). **(b)** Correlation between relative cutting frequency (INDEL% / target site frequency) and the CRISPR efficiency scores (Doench and Moreno), as well as target gene copy number and expression. *N* represents the selected locus-cell line combinations **(c)** Exemplary DNA gel electrophoresis result after homology directed repair template (HDRT) production using PCR and bead-based clean up. Uncropped source images are unavailable. Representative of n=2 biological replicates for each construct used, with similar results obtained in all replicates. **(d)** Transgene expression from the produced HDRTs as represented by the percentage of positive cells and mean fluorescence intensity (MFI) in a preliminary vector expression test using HEK293T cells and the Effectene transfection reagent. Dots and *n* represent biological replicates of transfection experiments. **Data presentation:** (a, d) Mean ± SD. **Statistical analysis:** (b) Linear regression for curve fitting with error bands representing the 95 % confidence interval, followed by two-sided Spearman correlation. *n.s.,* not significant. Source data and exact p values are provided as a Source Data file.

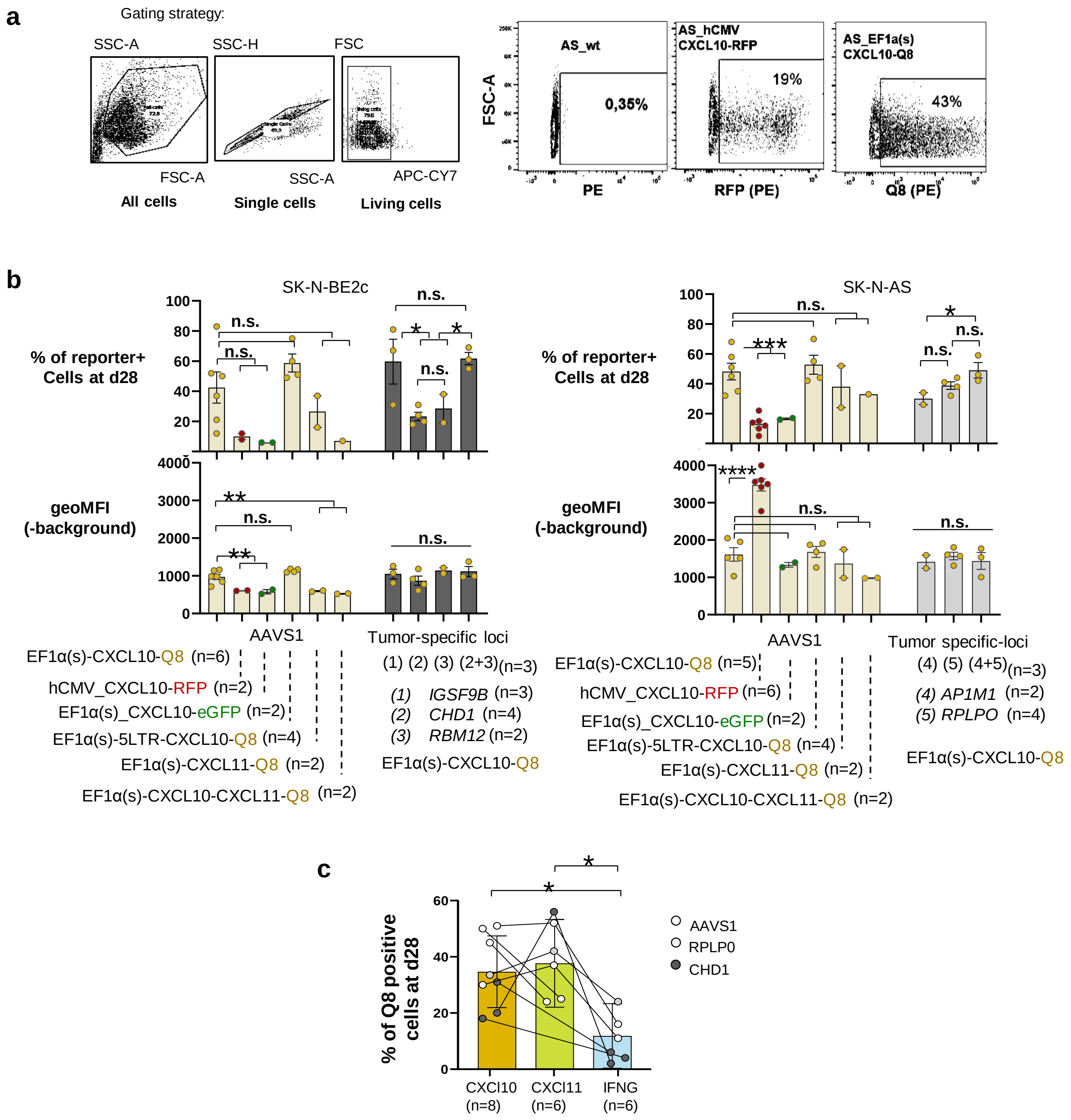

Supplementary Fig. 10. In vitro CRISPR knock-in. (a) Exemplary flow cytometry analysis and gating strategies of tumor cell lines 28 days after RNP + dsDNA template electroporation. Q8 reporter positivity was measured using PE-labeled CD34 Monoclonal Antibody QBEND/10 (Thermo Fisher). Representative of biological replicates of knock-in experiments shown in Figure 3d and Supplementary Fig. 10b with similar results obtained in all replicates. **(b)** Knock-in rates (%) and reporter expression strengths, represented by background-subtracted geometric mean fluorescence intensity (geoMFI), 28 days post-CRISPR KI for different constructs and loci in SK-N-BE2c and SK-N-AS neuroblastoma cell lines. Compared promoters include human CMV and a custom EF1α-derived promoter with or without a lentivirus-derived 5′ long terminal repeat (LTR) sequence. HDRT templates were designed for cytokines with a Q8 reporter and for CXCL10 with red (RFP) or green fluorescent protein (GFP) reporters. Additional tests included a tri-cistronic vector with two cytokines and a combinatorial knock-in using two HDRTs (differing homology arms) and guide RNAs (two tumor-specific loci) simultaneously without increasing total DNA or gRNA dose. In SK-N-BE2c, CHD1 and RBM12 were targeted; in SK-N-AS, AP1M1 and RPLP0. **(c)** Cumulative knock-in rate comparison for cytokine transgenes across three loci: AAVS1 (SK-N-BE2c and SK-N-AS), CHD1 (SK-N-BE2c only), and RPLP0 (SK-N-AS only). **Data presentation**: (b, c) Mean ± SD. Dots and *n* represent biological replicates (electroporation experiments followed by flow cytometry) **Statistical analysis**: (b) Kruskal-Wallis with Dunn's post hoc test; (c) Mixed-effects analysis with Tukey's multiple comparison test. *p* values: *<0.05, **<0.01, ***<0.001, ***<0.0001; *n.s.,* not significant.

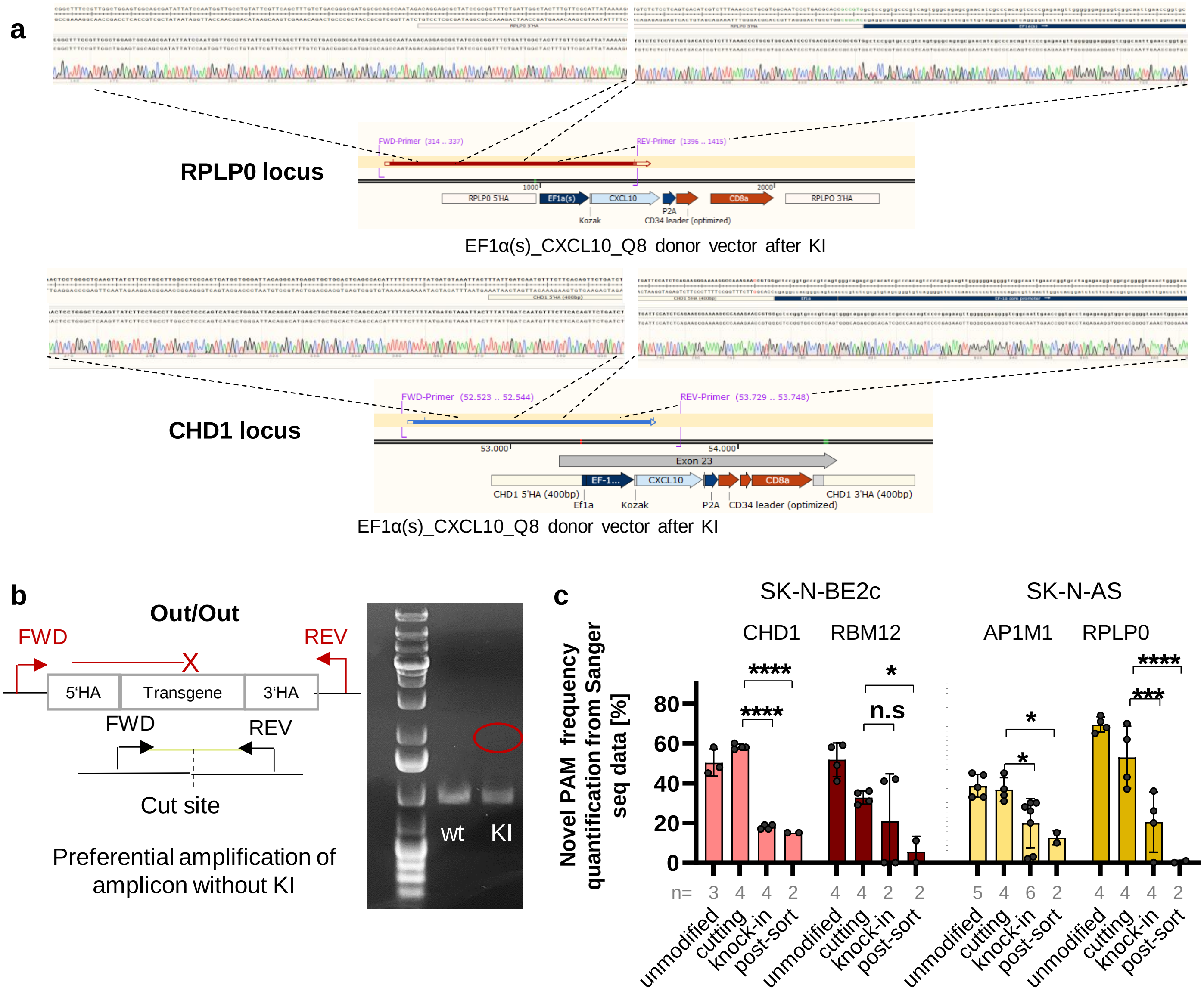

Supplementary Fig. 11. Site-specific knock-in confirmation via Sanger sequencing and PAM frequency analysis. (a) Schematic representation of on-target knock-in confirmation of an EF1α(s)_CXCL10_Q8 donor vector via PCR and Sanger sequencing with sequence alignment using SnapGene at the RPLP0 (SK-N-AS) and CHD1 (SK-N-BE2c) loci. **(b)** Schematic illustrating the preferential amplification of the non-knock-in amplicon using an Out/Out PCR, where forward and reverse primers are located upstream and downstream of the homology arms and cut site, complemented by representative gel electrophoresis results. Uncropped source images are unavailable. **(c)** Quantification of novel PAM frequency from Sanger sequencing data using EditR at the CHD1, RBM12, AP1M1, and RPLP0 loci in SK-N-BE2c and SK-N-AS cell lines. Data are shown for unmodified cells, cells treated with RNP only (cutting), and cells treated with RNP+HDRT (knock-in), with or without enrichment for reporter-positive cells. **Data presentation**: (c) Mean ± SD. Dots and *n* represent biological replicates of electroporation exerpiments ± enrichment followed by sanger sequencing and anlysis. **Statistical analysis**: (c) Two-way ANOVA with Dunnets post hoc test. *p* values: *<0.05, **<0.01, ***<0.001, ****<0.0001; *n.s.,* not significant. Source data and exact p values are provided as a Source Data file.

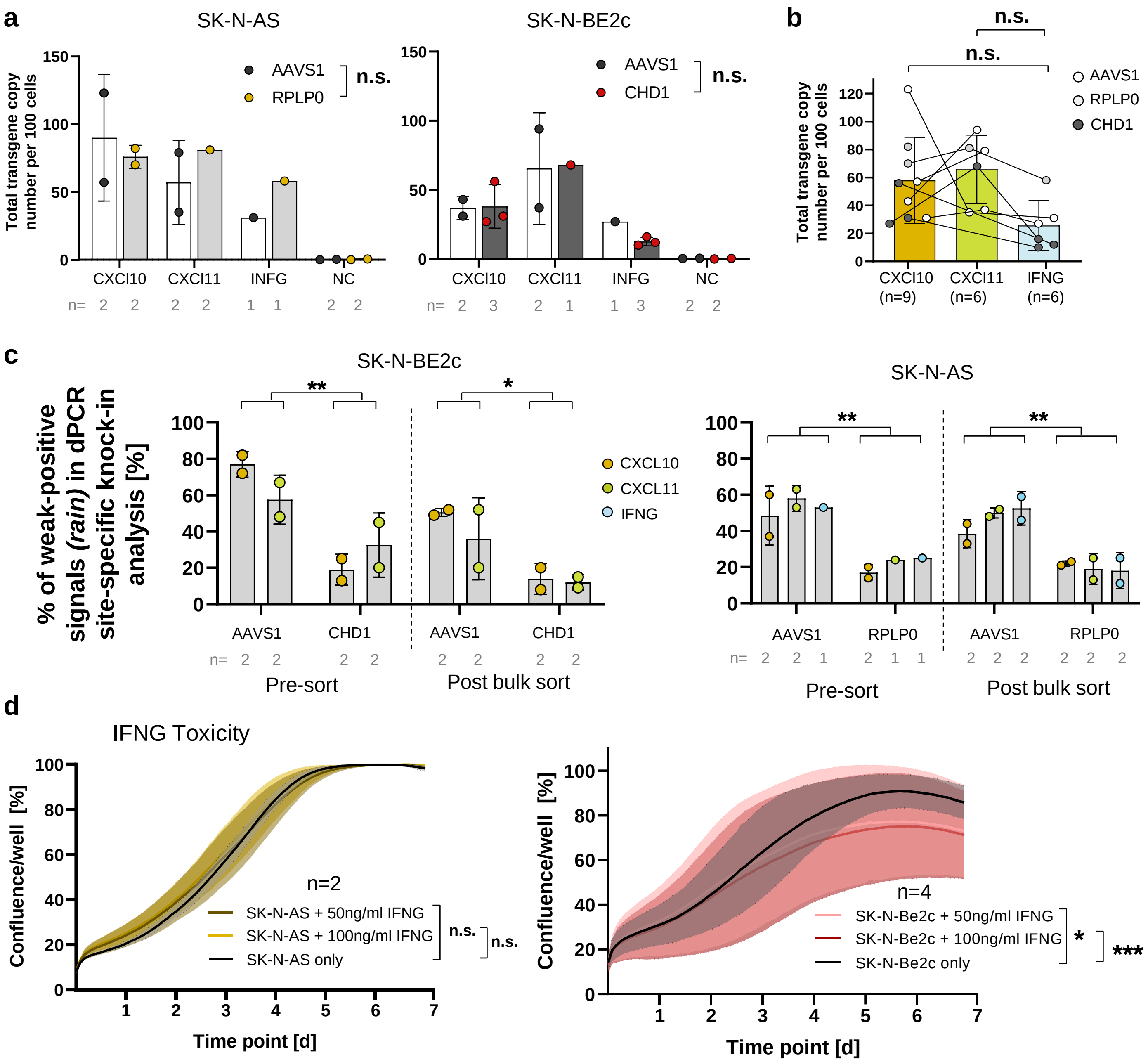

Supplementary Fig. 12. Site-specific knock-in analysis via dPCR and IFNG toxicity testing. (a) Total transgene copy number per 100 cells, 7 days after RNP/HDRT electroporation in SK-N-AS and SK-N-BE2c cell lines for different transgenes and target loci. **(b)** Paired analysis of cumulative total transgene copy number for the different cytokine transgenes CXCL10, CXCL11, IFNG. **(c)** Quantification of weak-positive signals ("rain") in digital PCR (dPCR) Out/In site-specific knock-in analysis for SK-N-BE2c and SK-N-AS neuroblastoma cell lines after HDRT knock-in at the AAVS1 control locus or novel PAM sites, before and after enrichment for reporter-positive cells.. **(d)** 7 day growth curves of unmodified SK-N-AS and SK-N-BE2c cell lines cultivated with varying concentrations of IFNG represented by confluence development recorded using Incucyte live imaging. **Data presentation**: (a-d) Mean ± SD. Dots and *n* represent biological replicates. **Statistical analysis**: (a-d) Two-way-ANOVA with Tukey test. *p* values: *<0.05, **<0.01, ***<0.001, ****<0.0001; n.s., not significant. Source data and exact p values are provided as a Source Data file.

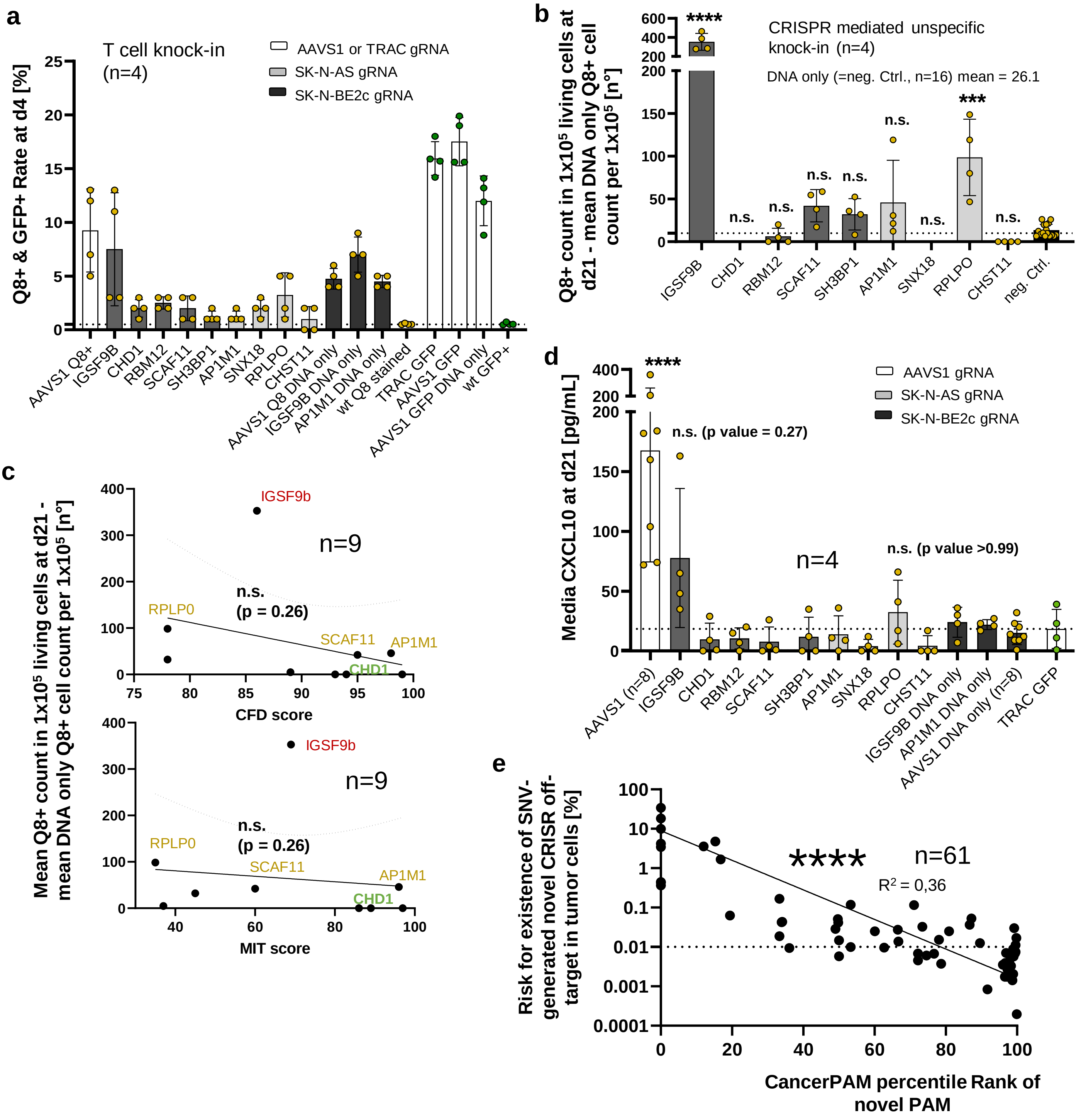

Supplementary Fig. 13. Unspecific knock-in. (a) Comparison of the percentage of reporter (Q8/GFP)-positive cells 4 days after RNP/HDRT electroporation in primary T cells from two donors using gRNAs targeting neuroblastoma-specific novel PAM sites or AAVS1 and TRAC control loci, measured via flow cytometry. **(b)** Analysis and comparison of CRISPR-mediated unspecific knock-in rates, calculated as the Q8⁺ count per 100,000 cells minus the mean Q8⁺ count per 100,000 cells in DNA-only control samples, via high-throughput flow cytometry (>300,000 cells analyzed per sample) for Q8-reporter harboring HDRTs targeted at neuroblastoma-specific PAM sites. **(c)** Correlation of the mean CRISPR-mediated unspecific T cell knock-in rates, analyzed via high-throughput flow cytometry and DNA-only background subtraction (b), with CRISPR efficiency scores (CFD and MIT). Dots and *n* represent mean values for selected targeted loci in T cells **(d)** Quantification of secreted CXCL10 two days after media change at day 21 post-RNP/HDRT electroporation in primary T cells from two donors using gRNAs targeting neuroblastoma-specific novel PAM sites or AAVS1 and TRAC control loci via ELISA. **(e)**. Calculated probability of tumor-specific CRISPR off-targets, against CancerPAM percentile rank for respective novel PAM sites across representative neuroblastoma patient samples. Dots and *n* represent individual PAM sites. **Data presentation**: (a,b,d) Mean ± SD. Dots and *n* represent biological replicates. **Statistical analysis**: (b,d) Kruskal-Wallis with Dunn's post hoc test; (c,e) (Log-)Linear regression for curve fitting with error bands representing the 95 % confidence interval, followed by two-sided Spearman correlation analysis. *p* values: *<0.05, **<0.01, ***<0.001, ****<0.0001; *n.s.,* not significant. Source data and exact p values are provided as a Source Data file.

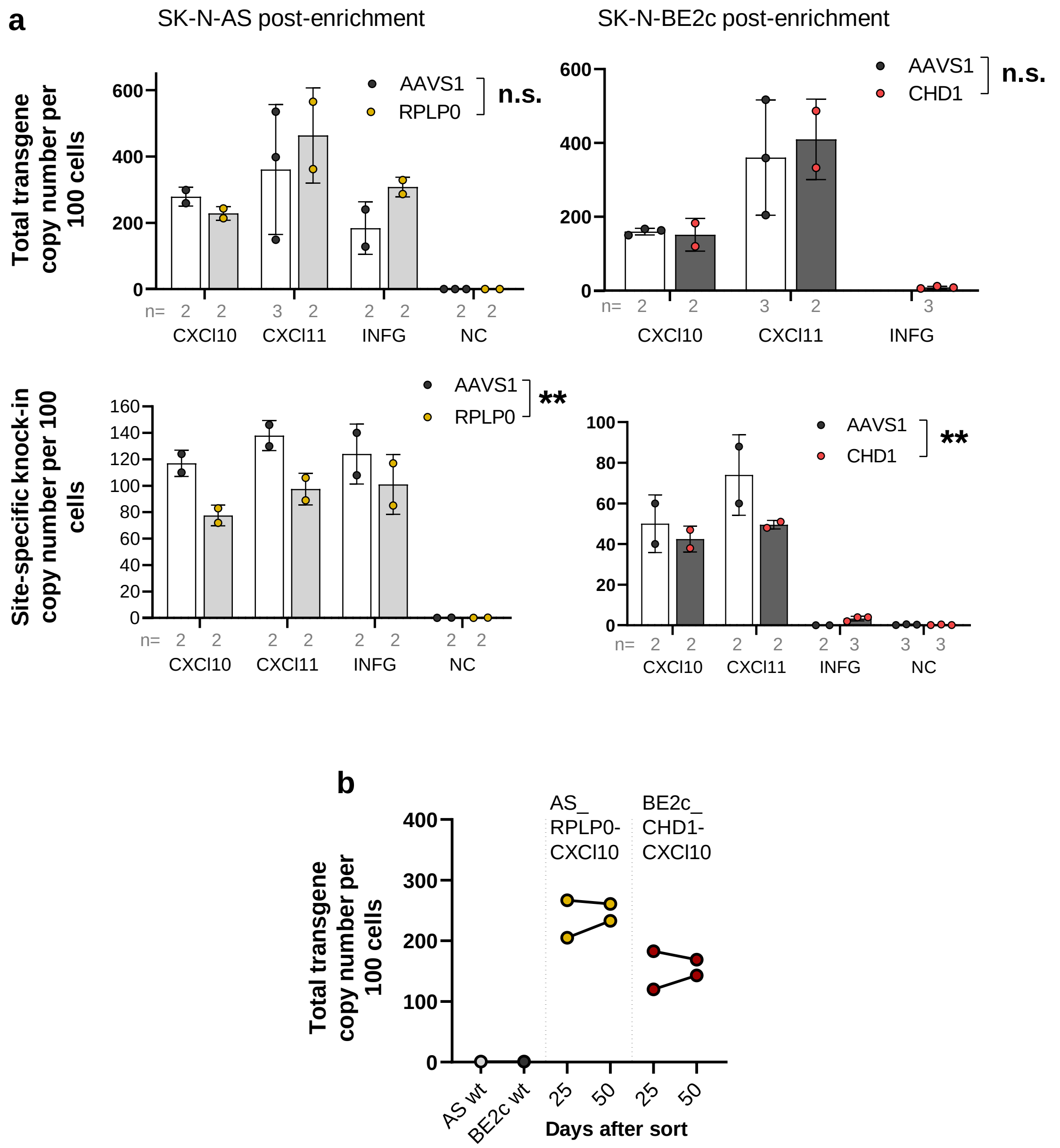

Supplementary Fig. 14. dPCR analysis after FACS enrichment. (a) Total transgene copy number and site-specific knock-in copy number per 100 cells after enrichment of SK-N-AS and SK-N-BE2c knock-in cell lines for different transgenes and target loci, determined via digital PCR (dPCR). **(b)** Total transgene copy number 25 and 50 days after enrichment in SK-N-AS CXCL10 RPLP0 and SK-N-BE2c CXCL10 CHD1 knock-in cell lines. **Data presentation**: (a) Mean ± SD. Dots and *n* represent biological replicates. **Statistical tests:** (a) Two-way ANOVA. *p* values: *<0.05, **<0.01, ***<0.001, ****<0.0001; *n.s.:* not significant. Source data and exact p values are provided as a Source Data file.

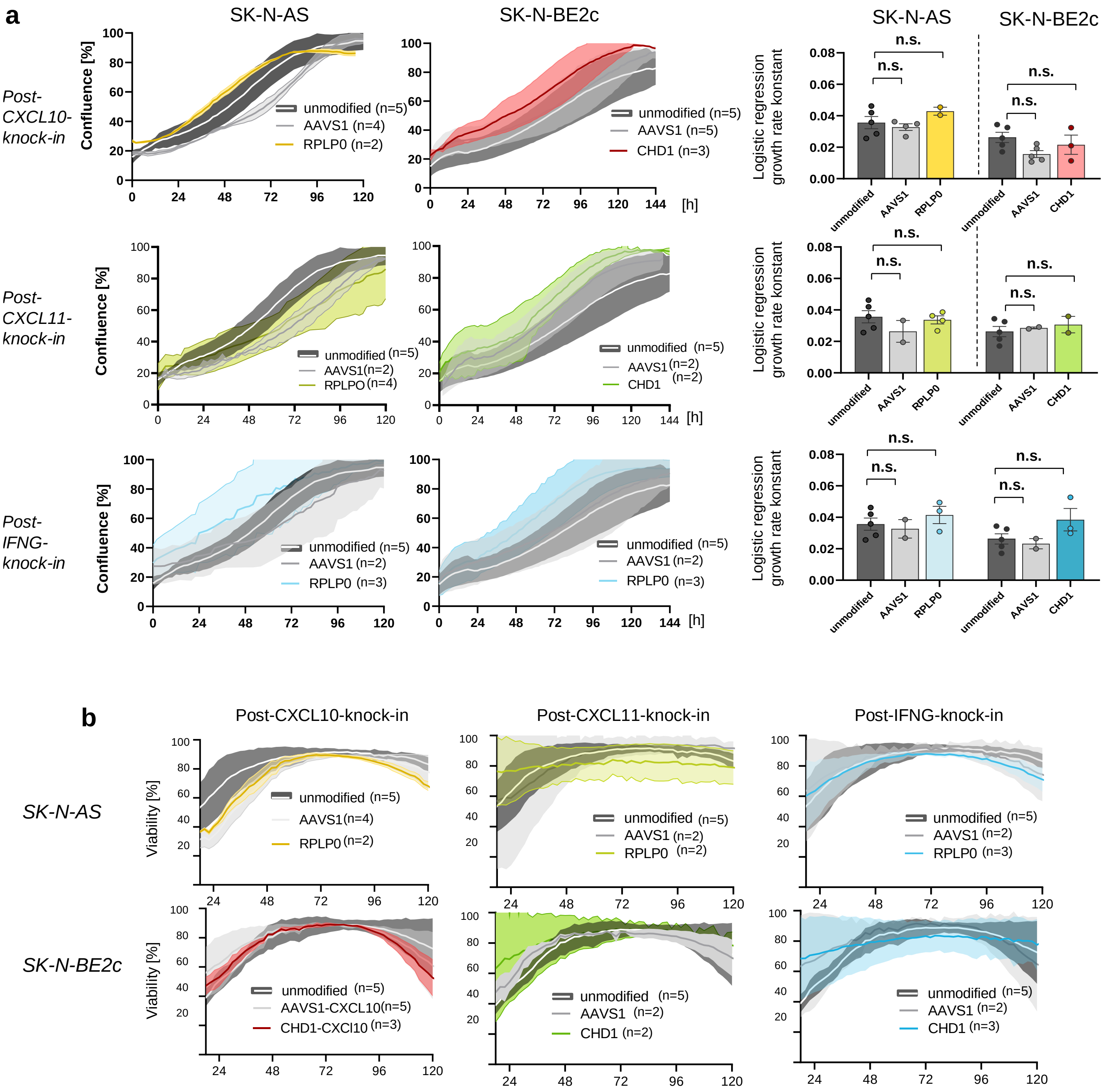

Supplementary Fig. 15. Characterization of enriched transgenic tumor cell lines. (a) Growth curves of unmodified or enriched SK-N-AS and SK-N-BE2c knock-in cell lines, represented by confluence development recorded using Incucyte live imaging, followed by growth rate comparison. **(b)** Viability curves of unmodified or enriched SK-N-AS and SK-N-BE2c knock-in cell lines, measured using Incucyte Cytotox Dye and Incucyte live imaging. **Data presentation**: (a,b) Mean ± SD. Dots and *n* represent biological replicates. **Statistical analysis**: (a) Kruskal-Wallis with Dunn's post hoc test; *n.s.,* not significant. Source data and exact p values are provided as a Source Data file.

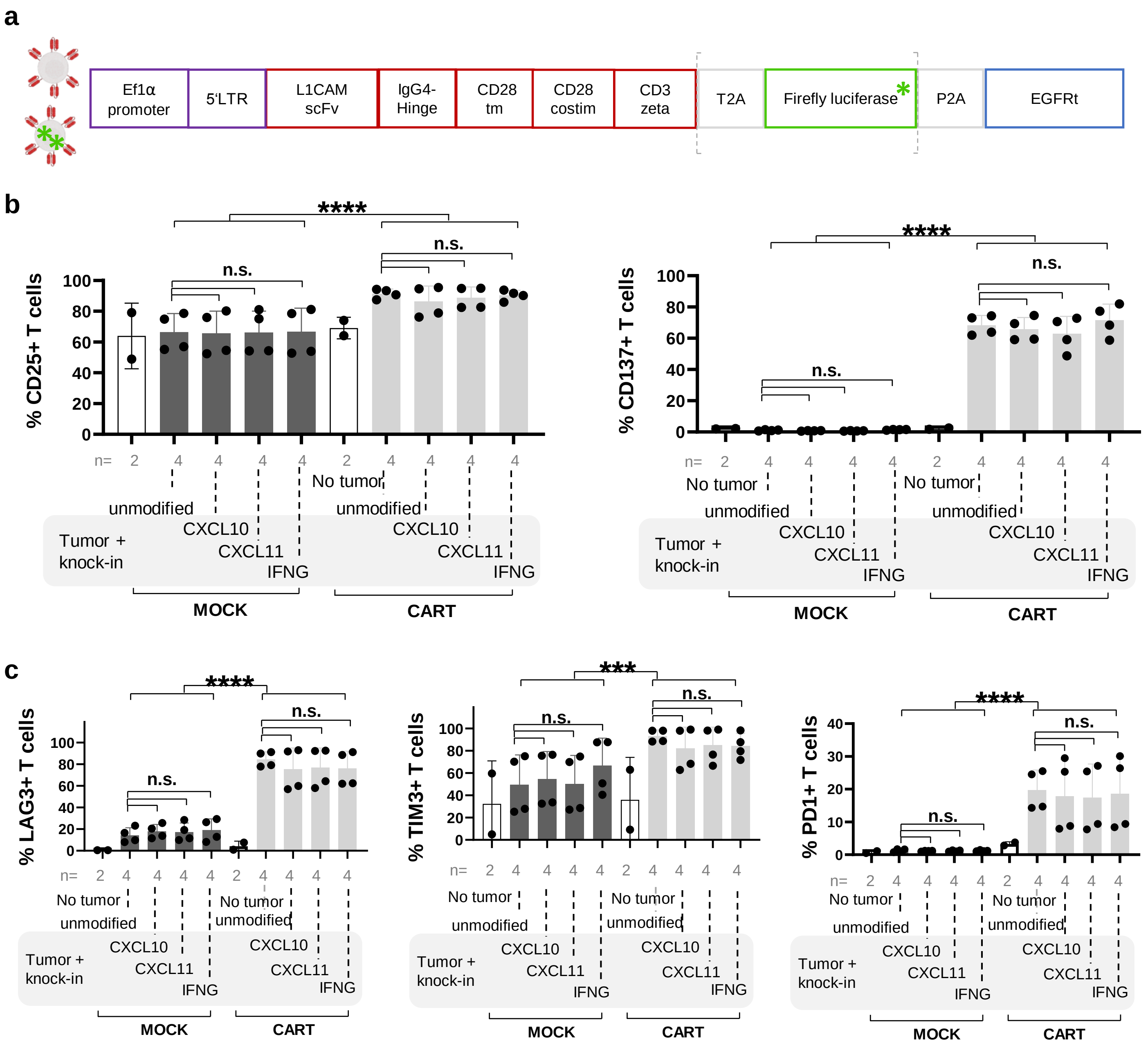

Supplementary Fig. 16. CAR T cell activation and exhaustion in tumor cell line-CAR T cell co-culture experiments. (a) Schematic overview of the two CAR constructs used in the study, featuring an EF1α promoter followed by a lentiviral 5′ long terminal repeat (LTR), a codon optimized L1CAM-targeting single-chain variable fragment (scFv), a short 12 amino acid long spacer domain from the immunoglobulin G4 (IgG4) Fc hinge, a CD28 transmembrane (tm) domain, a CD28 costimulatory (costim) domain, a CD3-zeta signaling domain, a T2A linker, firefly luciferase, a P2A linker, and a truncated non-functional epidermal growth factor receptor (EGFRt). **(b)** T cell activation, represented by CD25 and CD137 positivity, measured via flow cytometry 24 hours after co-culture initiation (see Fig. 4) for different tumor cell line–CAR T cell combinations. **(c)** T cell exhaustion, represented by LAG3, TIM3, and PD1 positivity, measured via flow cytometry under the same conditions. **Data presentation**: (a-c) Mean ± SD. Dots and *n* represent biological replicates. **Statistical analysis**: (a-c) Kruskal-Wallis with Dunn's post hoc test. *p* values: *<0.05, **<0.01, ***<0.001, ***<0.0001; *n.s.,* not significant. Source data and exact p values are provided as a Source Data file. **Illustration attribution:** CAR T cell illustration elements in (a): Created in BioRender. Kuenkele, A. (2025) https://BioRender.com/u71d212.

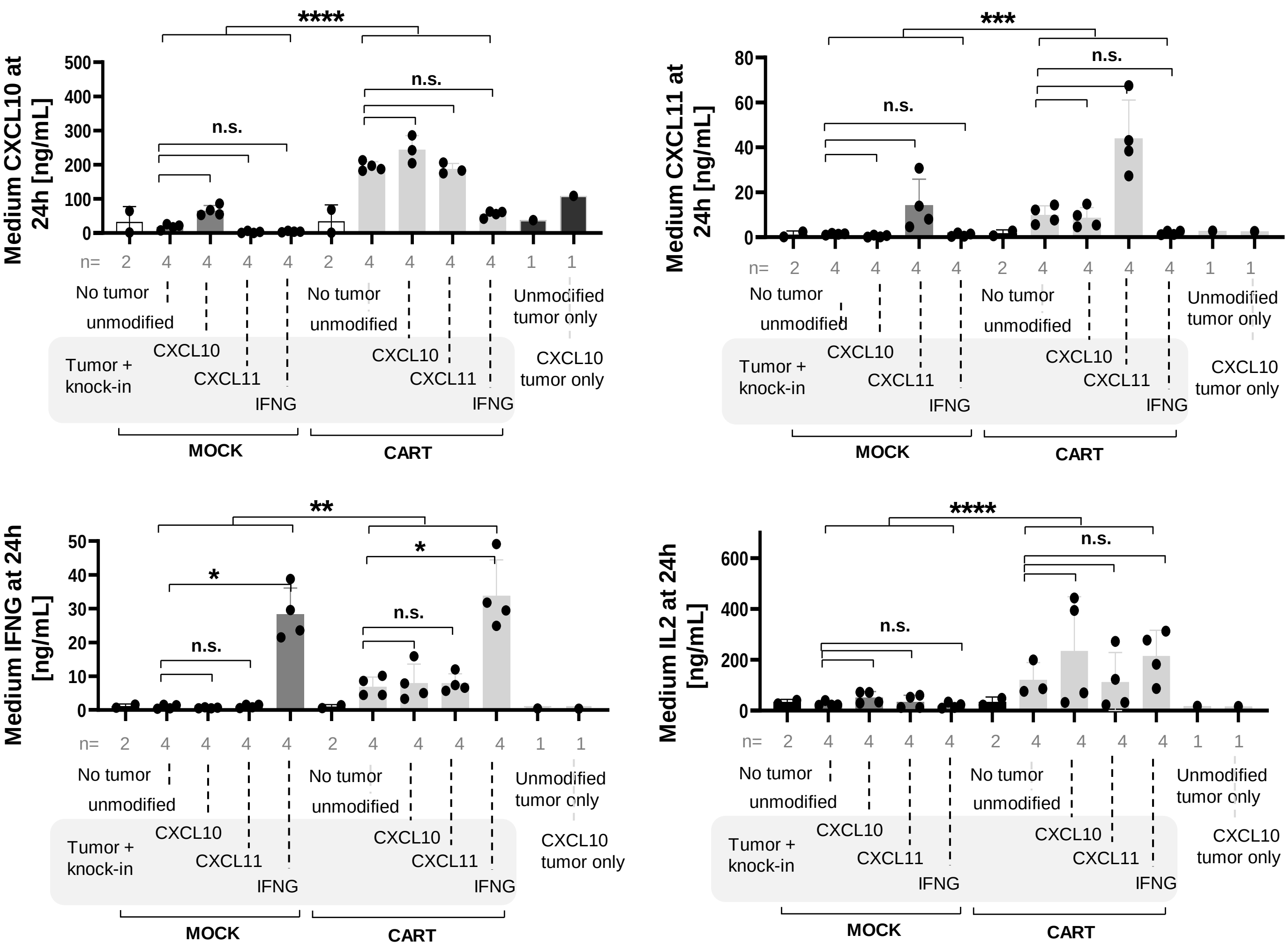

Supplementary Fig. 17. Cytokine secretion in tumor cell line-CAR T cell co-culture experiments. CXCL10, CXCL11, IFNG, and IL2 cytokine concentrations in the supernatant, determined via ELISA 24 hours after co-culture initiation. **Data presentation**: Mean ± SD. Dots and n represent biological replicates. **Statistical analysis**: Kruskal-Wallis with Dunn's post hoc test. p values: *<0.05, **<0.01, ***<0.001, ***<0.0001; n.s., not significant. Source data and exact p values are provided as a Source Data file.

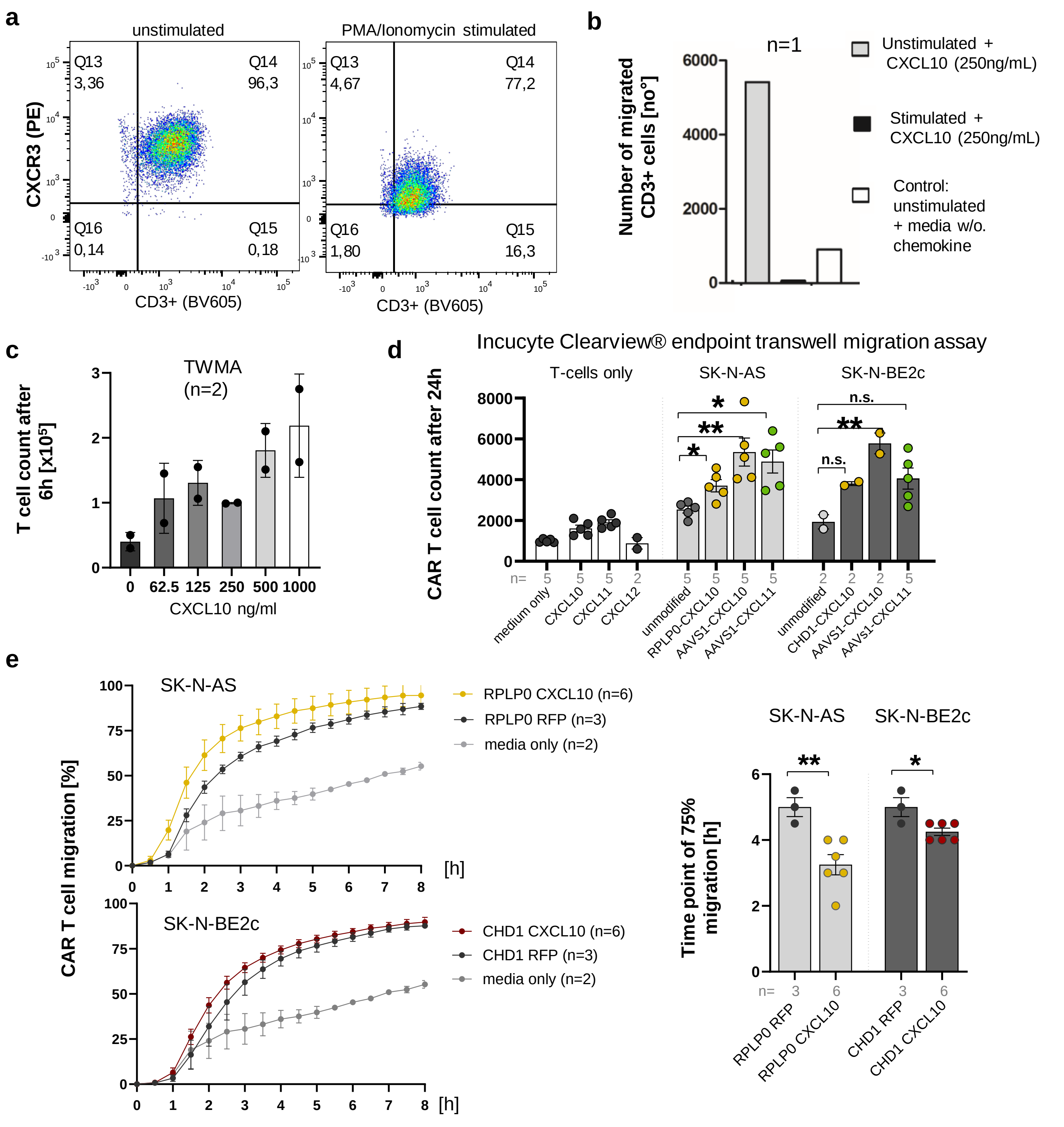

Supplementary Fig. 18. Cytokine-mediated T cell migration in vitro. (a) Flow cytometry data confirming CXCR3 expression, the receptor for CXCL10 and CXCL11, on unstimulated and PMA (Phorbol 12-Myristate 13-Acetate)/Ionomycin-stimulated T cells. **(b)** Preliminary Boyden chamber transwell migration assay (Corning) comparing the number of migrated CD3$^+$ T cells between unstimulated and PMA/Ionomycin-stimulated conditions. **(c)** Boyden chamber transwell migration assay comparing the number of migrated CD3$^+$ T cells in response to increasing CXCL10 concentrations. **(d)** Incucyte Clearview endpoint transwell migration assay (Sartorius) measuring the number of migrated L1CAM-targeting CD3$^+$ CAR T cells after 24 hours in either media only, recombinant cytokines, or supernatants from unmodified or knock-in SK-N-AS and SK-N-BE2c cell lines. **(e)** Migration curves generated using a Boyden chamber transwell migration assay (Corning) and Incucyte live imaging (Sartorius) to determine the migration rate of CD3$^+$ CAR T cells in response to supernatants from transgenic SK-N-AS and SK-N-BE2c cell lines (CXCL10-expressing or RFP) or media only, followed by statistical comparison of the time points at which 75% of migration was reached. **Data presentation**: (c-e) Mean ± SD. Dots and *n* represent biological replicates. **Statistical analysis**: (d, e) Kruskal-Wallis with Dunn's post hoc test. *p* values: *<0.05, *<0.01; *n.s.*, not significant. Source data and exact p values are provided as a Source Data file.

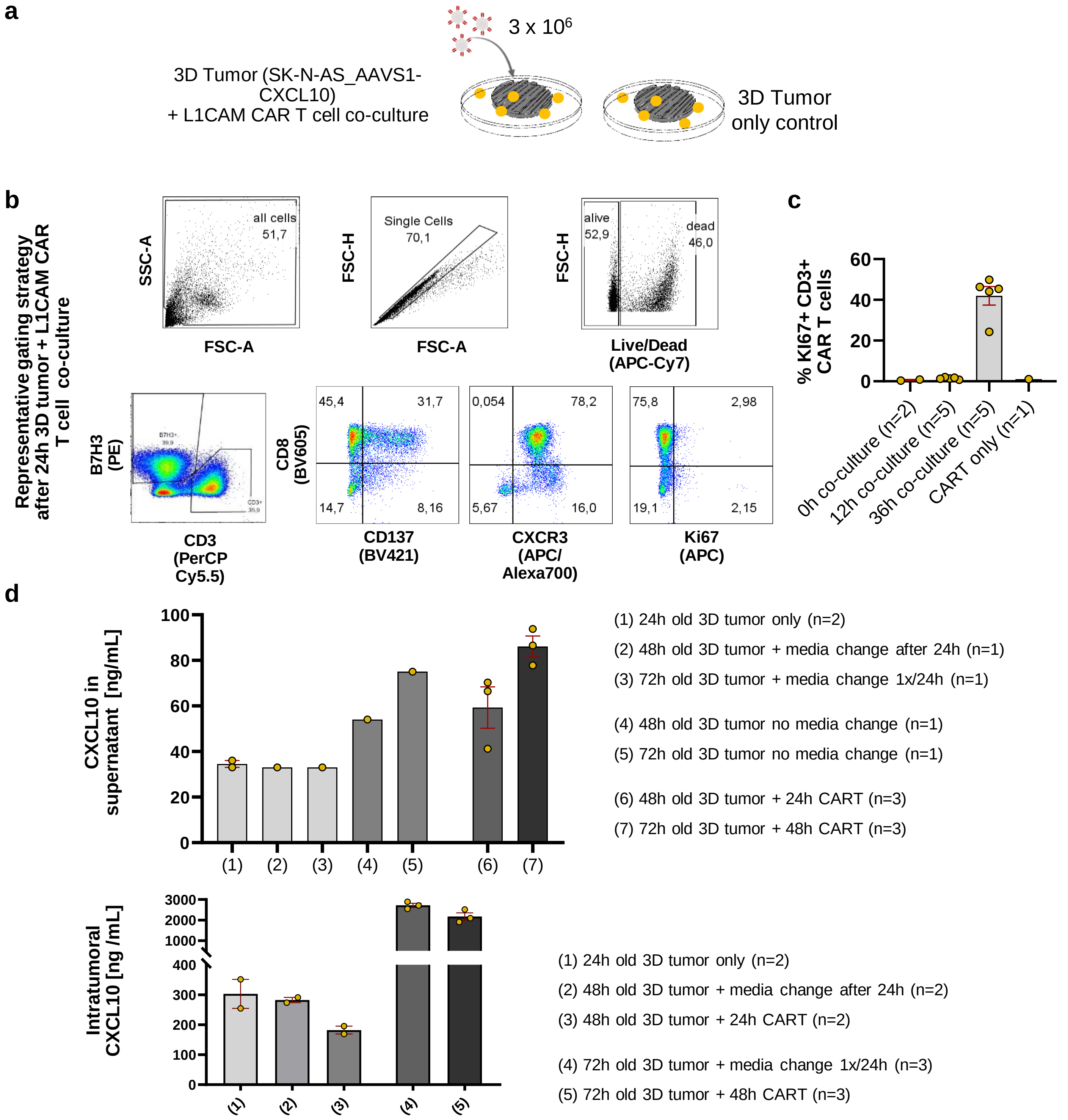

Supplementary Fig. 19. 3D tumor infiltration assay. (a) Schematic overview of the preliminary 3D bioprinted SK-N-AS tumor model and L1CAM-targeting CAR T cell co-culture. **(b)** Representative flow cytometry gating strategy 24 hours after 3D bioprinted SK-N-AS tumor-L1CAM CAR T cell co-culture, distinguishing T cells and tumor cells via B7H3 and CD3 surface markers. Representative of biological replicates shown in Figure 5e and f, with similar results obtained in all replicates. **(c)** KI67 proliferation marker positivity in CD3^{+} CAR T cells at different time points following 3D tumor–CAR T cell co-culture initiation. **(d)** CXCL10 concentration measured by ELISA in the supernatant or within the 3D tumor construct under various co-culture conditions. **Data presentation**: (c, d) Mean ± SD. Dots and *n* represent biological replicates. Source data are provided as a Source Data file. **Illustration attribution:** CAR T cell illustration elements in (a): Created in BioRender. Kuenkele, A. (2025) https://BioRender.com/u71d212.

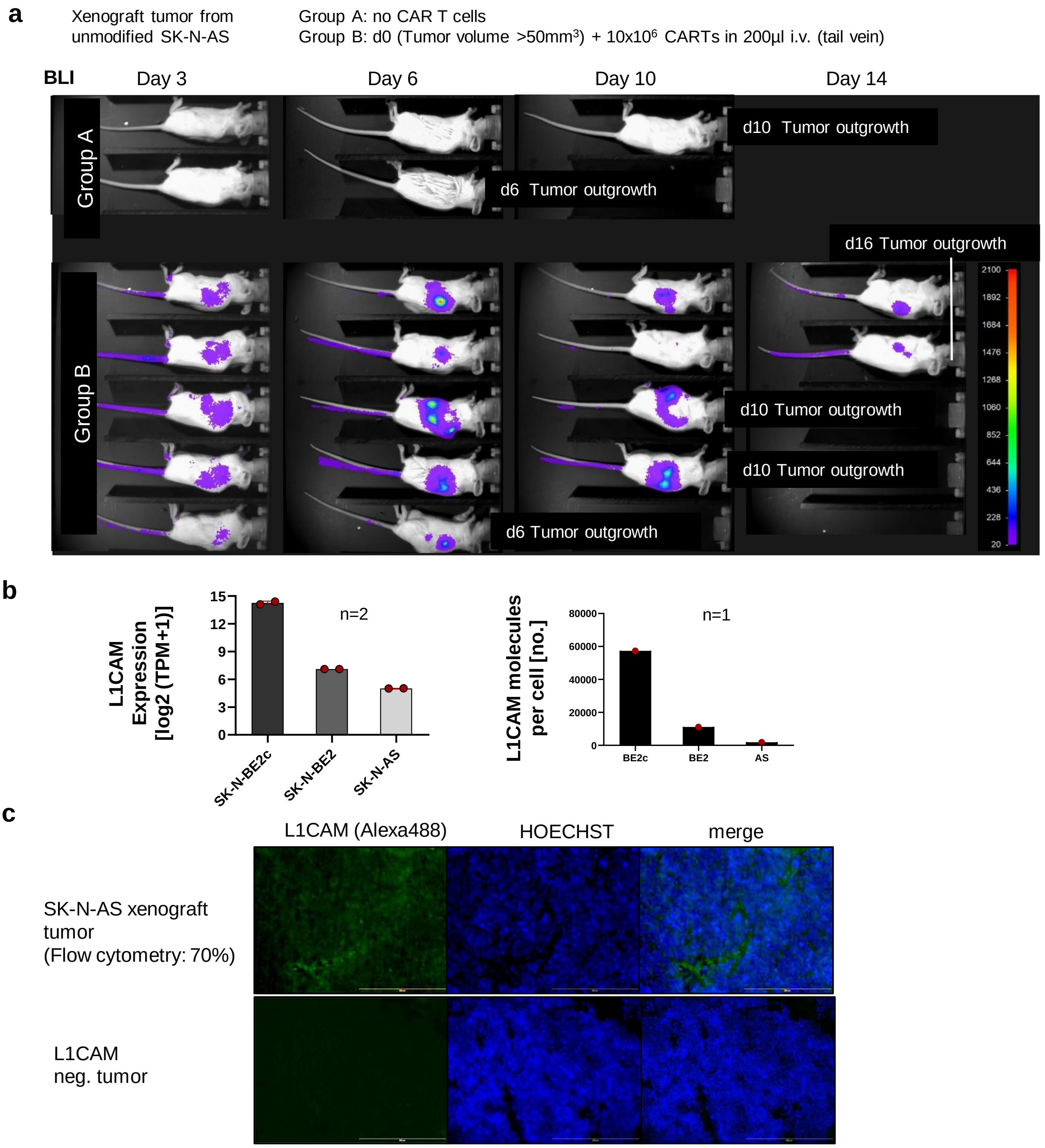

Supplementary Fig. 20. In vivo CAR T cell infiltration model development. (a) Preliminary in vivo CAR T cell infiltration analysis in a xenograft mouse model using unmodified SK-N-AS neuroblastoma cells and L1CAM-targeting, firefly luciferase-expressing CAR T cell treatment after tumor engraftment (tumor size >50 mm^3), followed by regular monitoring and bioluminescence imaging to assess CAR T cell migration, tumor infiltration, and expansion. **(b)** L1CAM expression levels from DepMap 24Q2 and 24Q4 datasets and L1CAM molecule per cell count as determined via QuantiBRITE PE bead surface expression analysis. **(c)** Immunofluorescence staining of two different neuroblastoma PDX mouse models, one L1CAM-positive and one L1CAM-negative. **Data presentation:** *(b) Mean ± SD.* Dots and *n* represent biological replicates. Source data are provided as a Source Data file.

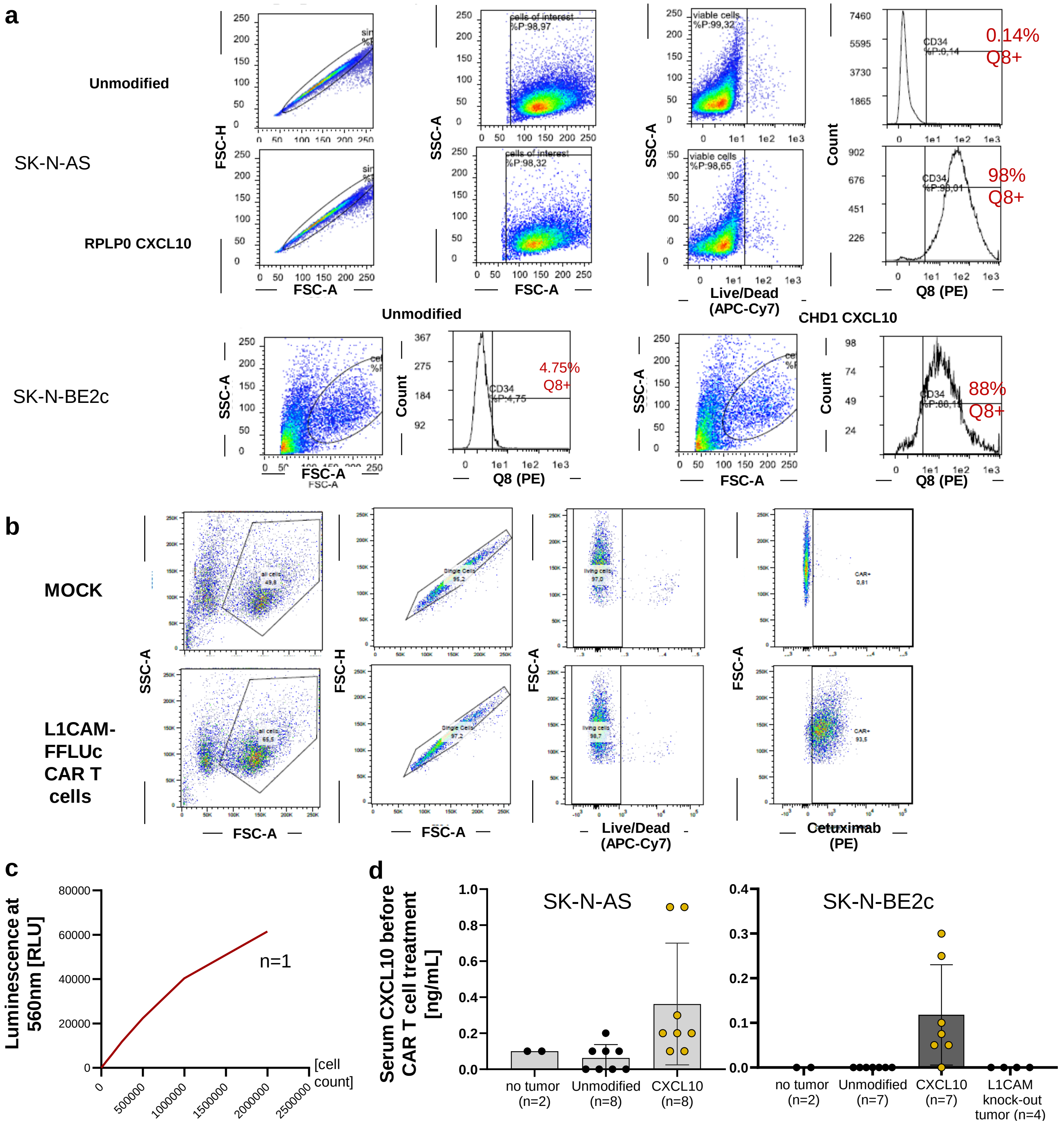

Supplementary Fig. 21. In vivo CAR T cell infiltration experiment: pre-transplant and pre-treatment analysis. (a) Confirmation of transgene expression in SK-N-AS and SK-N-BE2c neuroblastoma cell lines, assessed via Q8 positivity using flow cytometry. **(b)** CAR expression confirmation before in vivo application, determined by flow cytometry in CAR T cells after transduction with an L1CAM CAR, firefly luciferase, and EGFRt reporter-expressing lentiviral vector, followed by expansion. Cetuximab staining was used for EGFRt detection on the cell surface. **(c)** In vitro bioluminescence intensity measured for different L1CAM-targeting, firefly luciferase-expressing CAR T cell counts after luciferin incubation. **(d)** Serum CXCL10 levels on day 0, measured by ELISA in NSG mice harboring unmodified or CXCL10-expressing SK-N-AS or SK-N-BE2c xenograft tumors, directly before CAR T cell treatment. **Data presentation**: (b) Mean ± SD. Dots and *n* represent biological replicates. Source data are provided as a Source Data file.

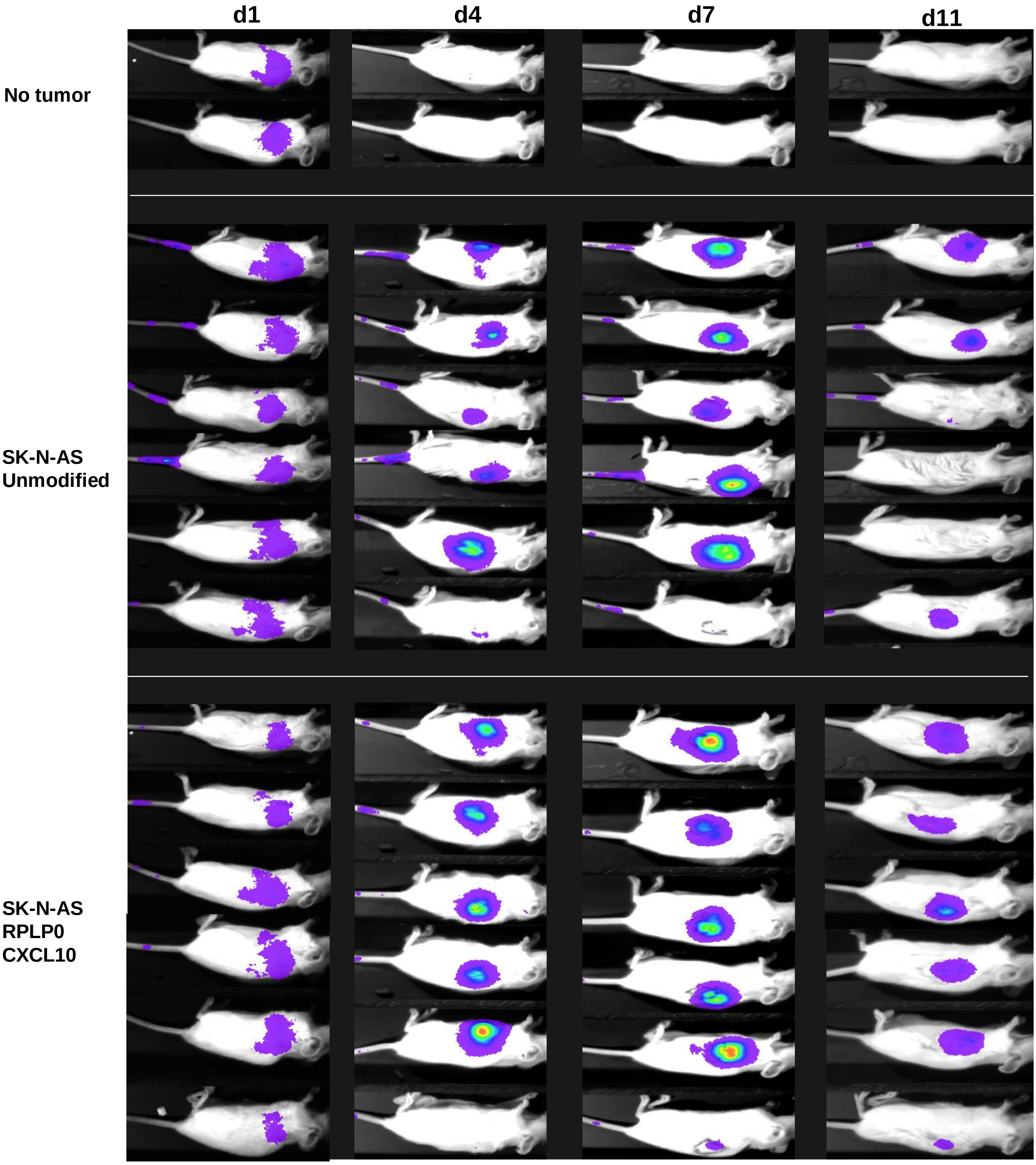

Supplementary Fig. 22. Bioluminescence imaging of CAR T cell treated SK-N-AS xenograft neuroblastoma models. Imaging timepoints are given relative to treatment with L1CAM targeting, luciferase expressing human CAR T cells. Mice with tumor volumes of >1200-1500mm^3 were removed from the study. Raw images from d14 to d30 are not shown.

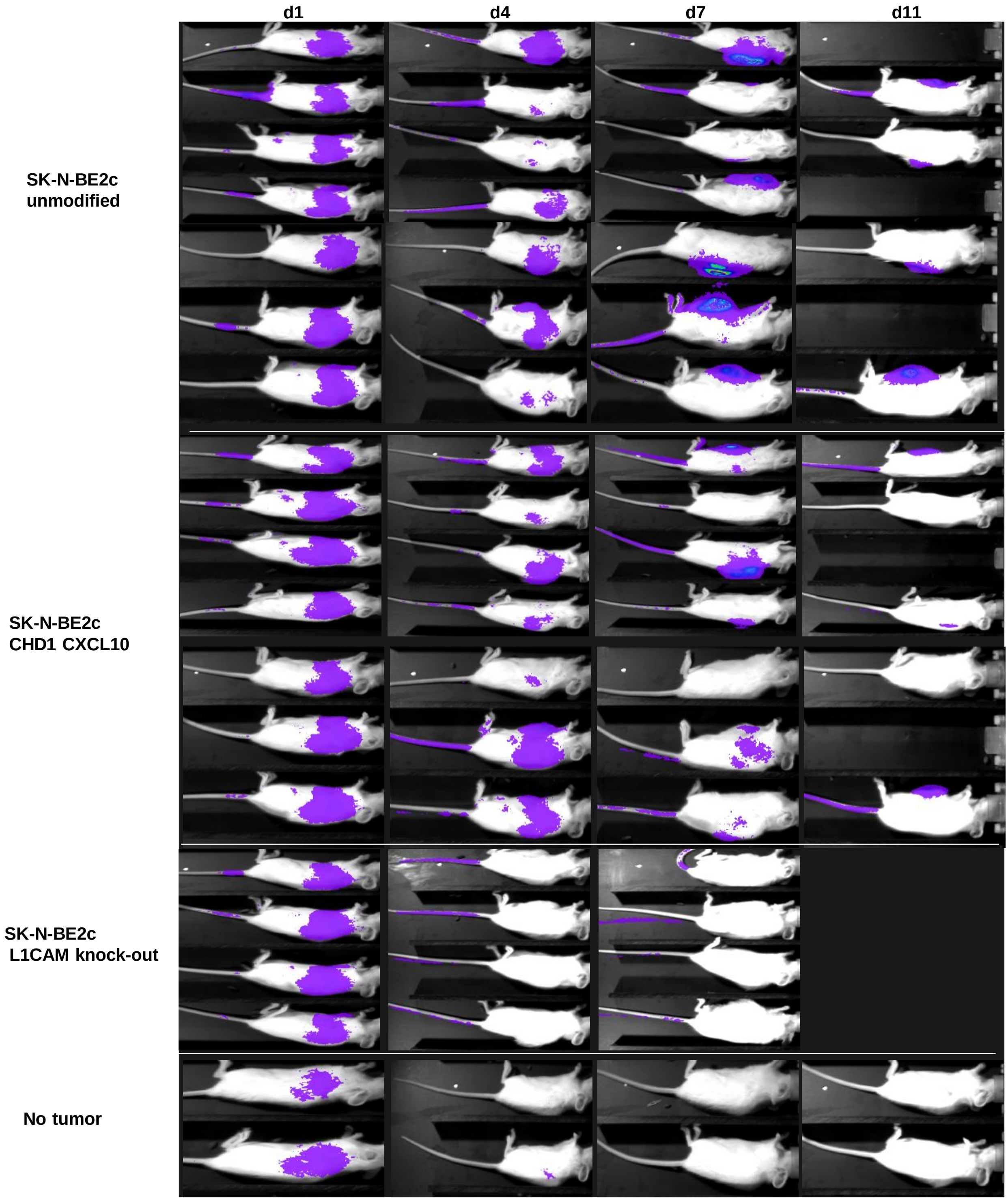

Supplementary Fig. 23. Bioluminescence imaging of CAR T cell treated SK-N-BE2c xenograft neuroblastoma models. Imaging timepoints are given relative to treatment with L1CAM targeting, luciferase expressing human CAR T cells. Mice with tumor volumes of >1200-1500mm^3 were removed from the study. Raw images from d14 to d30 are not shown.

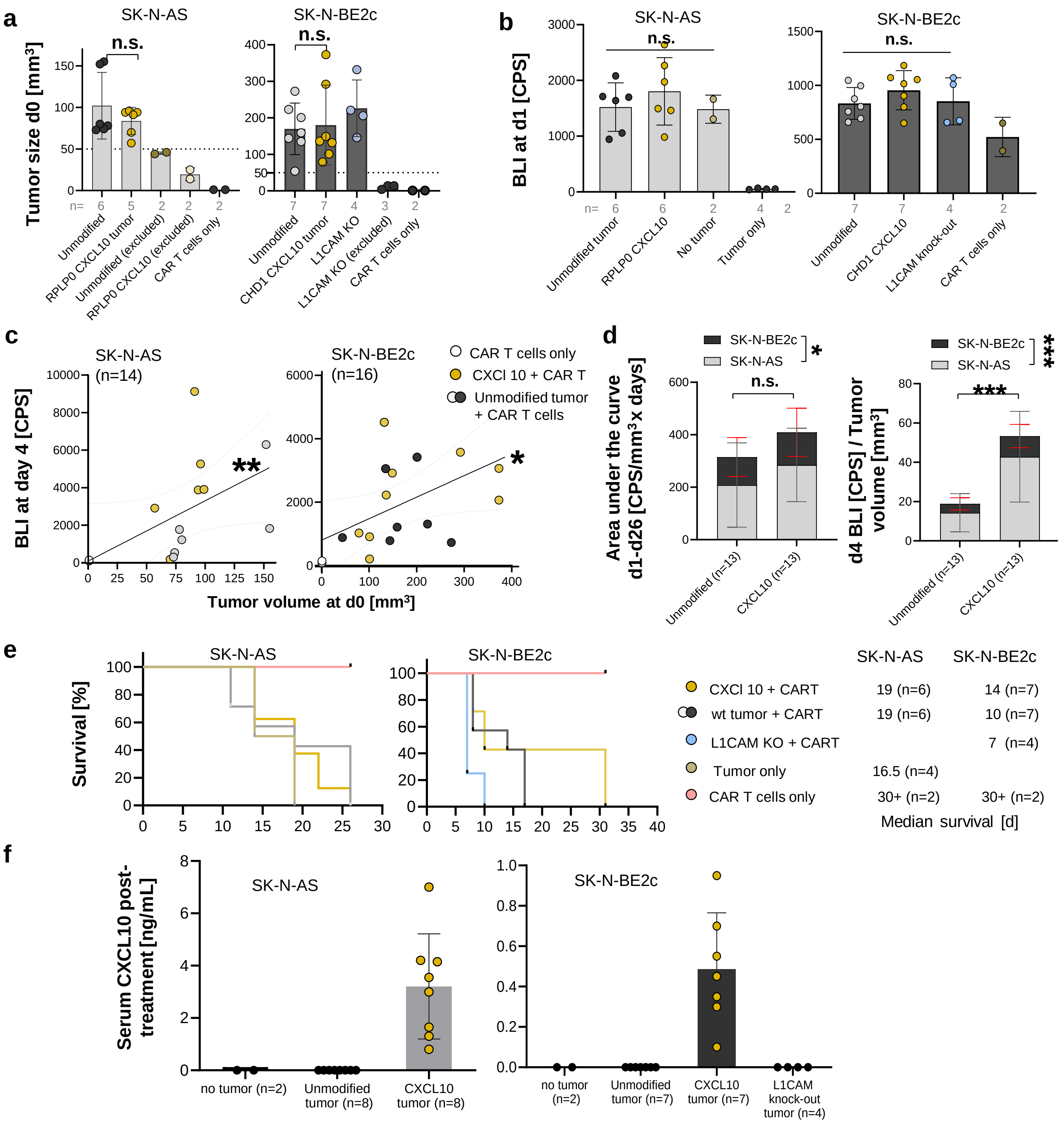

Supplementary Fig. 24. In vivo CAR T cell infiltration in CXCL10-secreting xenograft tumors. (a) Manually measured tumor size on the day of CAR T cell injection (d0), compared across treatment groups; only animals with tumors bigger than 50 mm³ received CAR T cell treatment. **(b)** Bioluminescence measurement in the lung on day 1 after treatment with L1CAM-targeting, firefly luciferase-expressing CAR T cells. **(c)** Correlation between the bioluminescence signal in the left flank on day 4 and tumor volume at day 0. **(d)** Cumulative comparison of total tumoral CAR T cell infiltration and expansion, represented by the area under the curve (AUC) of the bioluminescence-to-tumor volume ratio, and early infiltration based on the bioluminescence-to-tumor volume ratio on day 4, combining SK-N-AS and SK-N-BE2c data. **(e)** Survival curves for different SK-N-AS and SK-N-BE2c xenograft mouse model treatment groups after CAR T cell treatment (d0), including median survival. **(f)** Serum CXCL10 levels measured on the day of tumor outgrowth and animal removal after CAR T cell treatment via ELISA. **Data presentation**: (a, b, d, f) Mean ± SD. (a-f) Dots and *n* represent individual animals. (E) Kaplan-Meier survival curves. **Statistical analysis:** (a,c) Kruskal-Wallis with Dunn's post hoc test; (b) Linear regression for curve fitting with error bands representing the 95 % confidence interval, followed by two-sided Spearman correlation analysis. (d) Two-way ANOVA. *p* values: *<0.05, **<0.001; *n.s.*, not significant. Source data and exact p values are provided as a Source Data file.

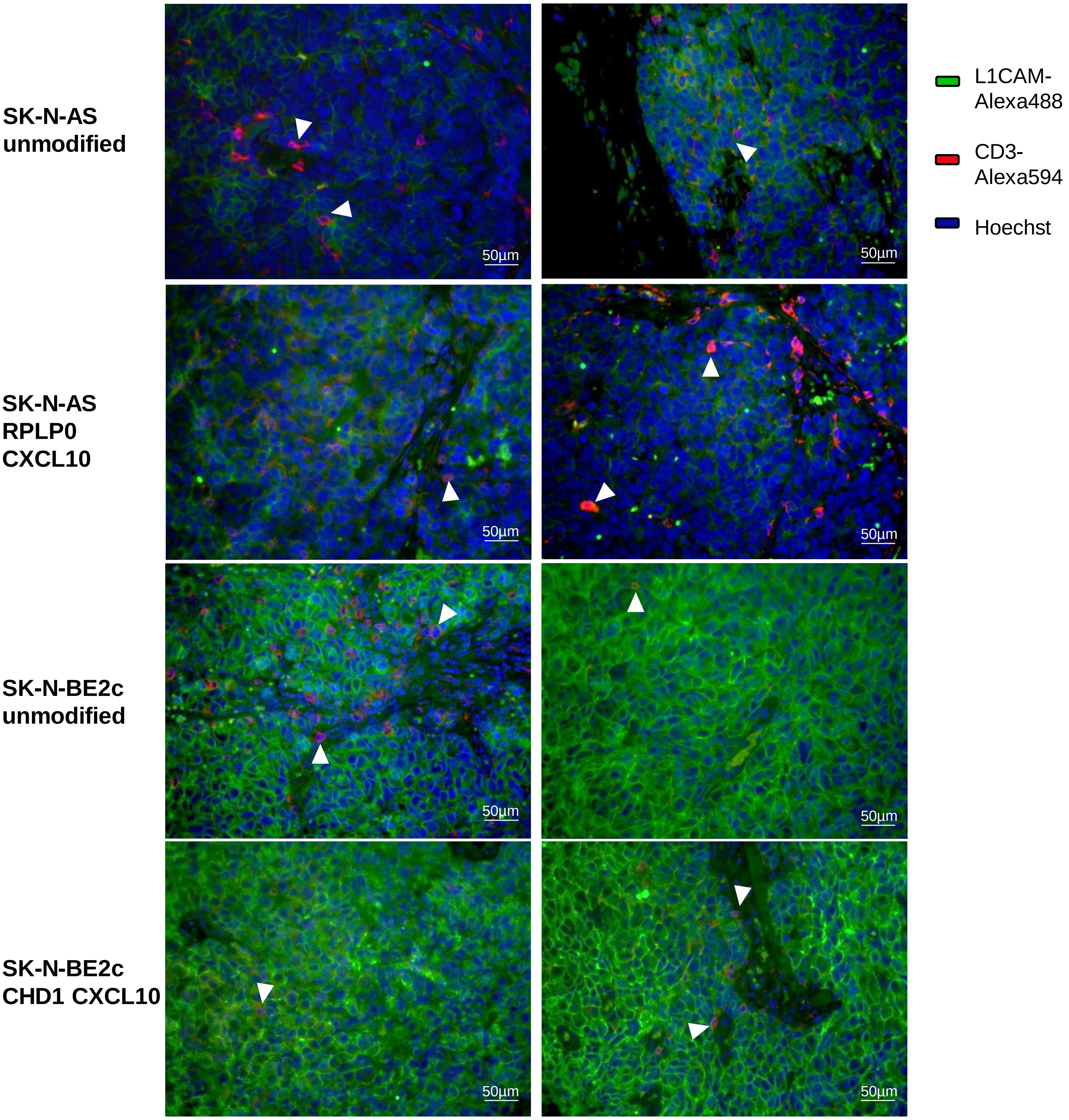

Supplementary Fig. 25. Immunofluorescence staining of tumor sections. Representative 40x magnification images of L1CAM and CD3 immunofluorescence staining of tumor sections at the time point of outgrowth and animal removal after treatment with L1CAM-targeting, firefly luciferase-expressing CAR T cells. White arrows indicate examples of intratumoral $CD3^{+}$ T cells. Representative of n=4 tumors per group (unmodified or CXCL10), with similar results obtained in all replicates.

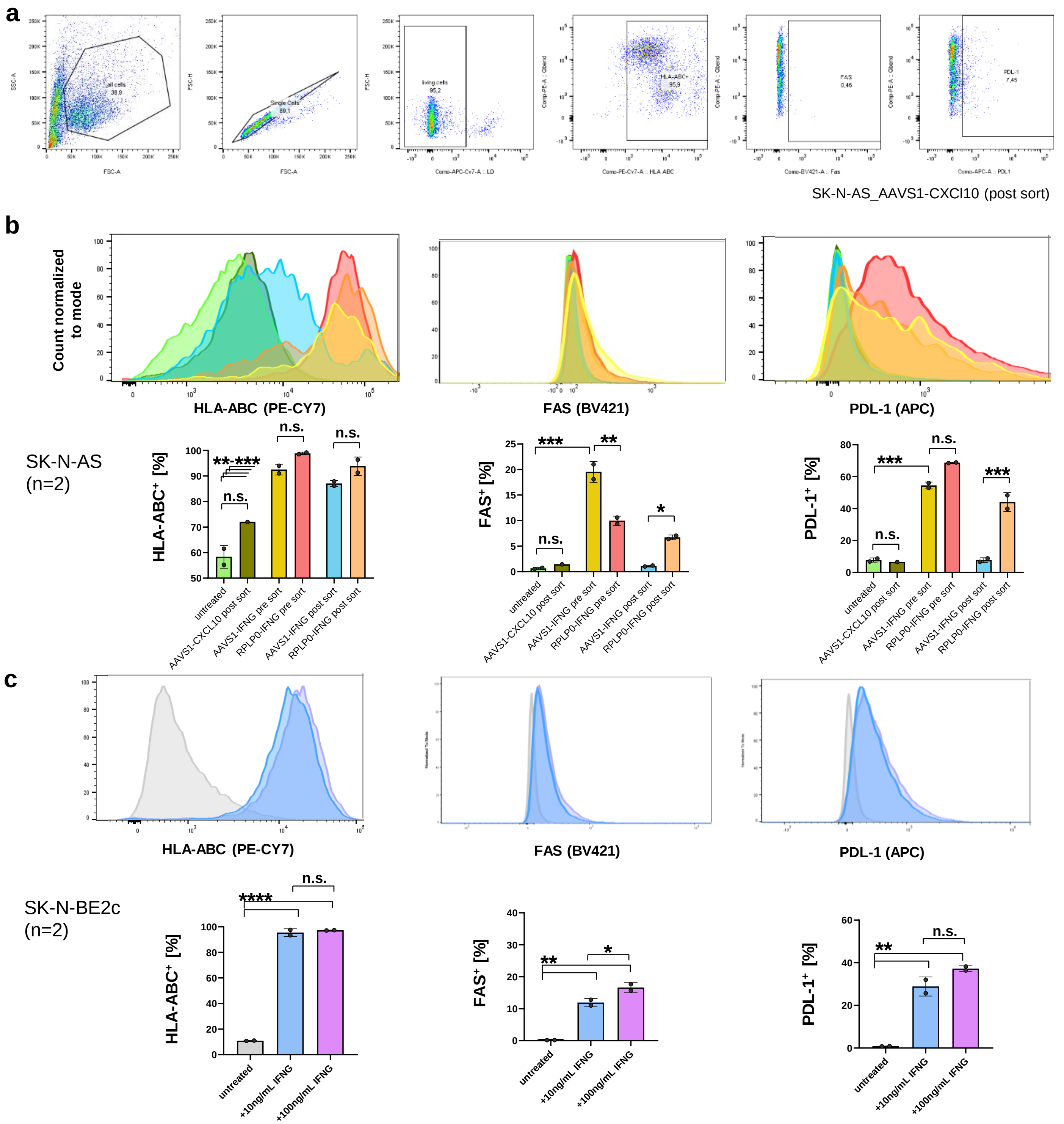

Supplementary Fig. 26. Flow cytometric analysis of IFNG-mediated regulation of surface markers in neuroblastoma cell lines. (a) Representative gating strategy for live, single tumor cells and surface marker assessment. Representative of n=2 biological replicates shown in b and c, with similar results obtained in all replicates. **(b)** Flow cytometry histograms and quantification of HLA-ABC, FAS, and PD-L1 expression in SK-N-AS cells with or without IFNG expression following CRISPR-mediated targeting at AAVS1 or RPLP0 loci, before and after enrichment. **(c)** Flow cytometry histograms and quantification of HLA-ABC, FAS, and PD-L1 expression in SK-N-BE2c cells with or without IFNG expression. **Data presentation**: (b,c) Mean ± SD. Dots and *n* represent biological replicates. **Statistical analysis**: (b,c) One-Way ANOVA + Tukey's multiple comparisons test. *p* values: *<0.05, **<0.01, ***<0.001, ***<0.0001; *n.s.,* not significant. Source data and exact p values are provided as a Source Data file.

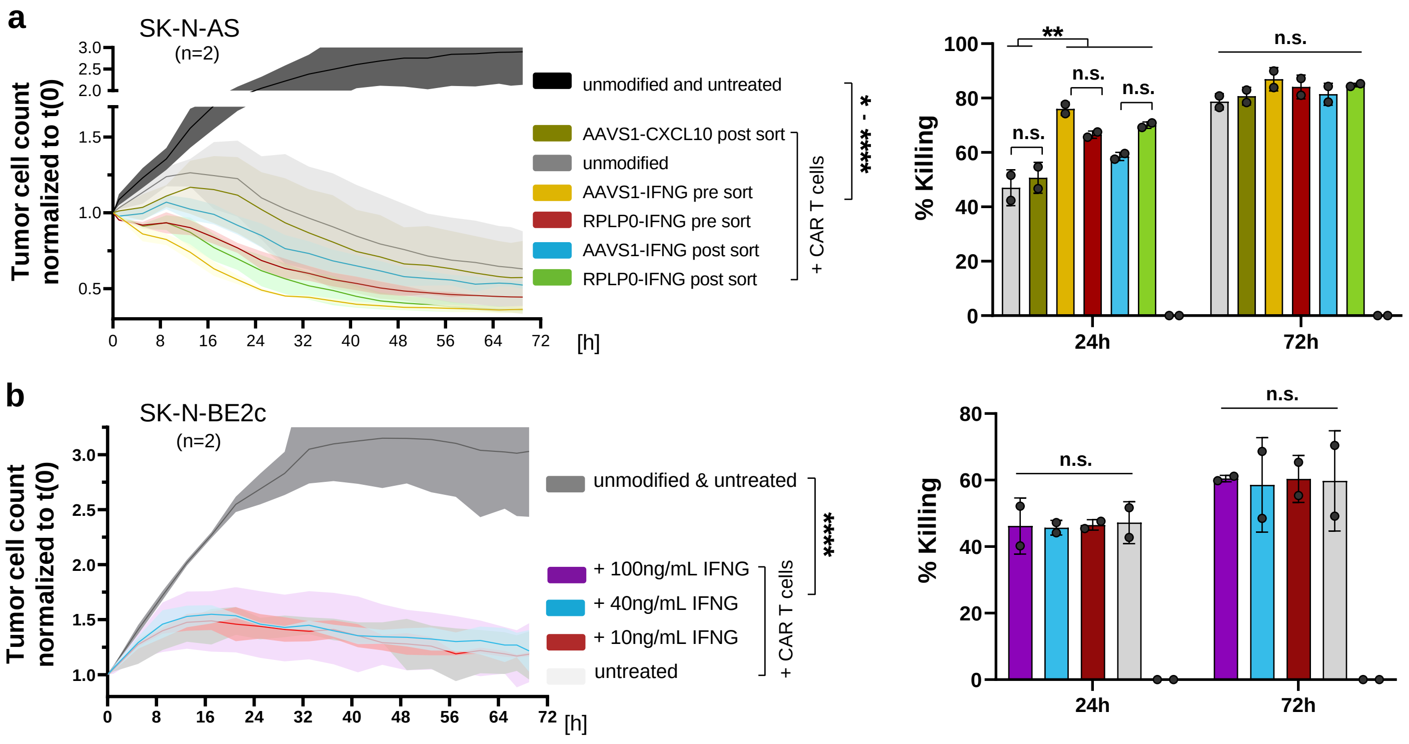

Supplementary Fig. 27. Impact of IFNG expression or supplementation on CAR T cell-mediated killing of neuroblastoma cells. (a) Real-time live-imaging based cytotoxicity assay of SK-N-AS cells (low L1CAM expression) with IFNG knock-in at AAVS1 or RPLP0 loci (pre-sort and post-sort) co-cultured with L1CAM-directed CAR T cells. Left: tumor cell growth kinetics normalized to baseline (t0). Right: quantification of CAR T cell–mediated killing at 24 h and 72 h relative to unmodified and untreated control. **(b)** Real-time live-imaging based cytotoxicity assay of SK-N-BE2c cells (high L1CAM expression) co-cultured with CAR T cells in the presence of recombinant IFNG at varying concentrations (10–100 ng/mL). Left: tumor cell growth kinetics normalized to baseline. Right: quantification of CAR T cell-mediated killing at 24 h and 72 h relative to unmodified and untreated control. **Data presentation**: (a,b) Mean ± SD. Dots and *n* represent biological replicates. **Statistical analysis**: (a,b) left: Two-way ANOVA with Tukey's multiple comparisons test for CAR T cell vs. MOCK comparison, right: Sidak's multiple comparisons test; *p* values: *<0.05, **<0.01, ***<0.001, ***<0.0001; *n.s.*, not significant. Source data and exact p values are provided as a Source Data file.

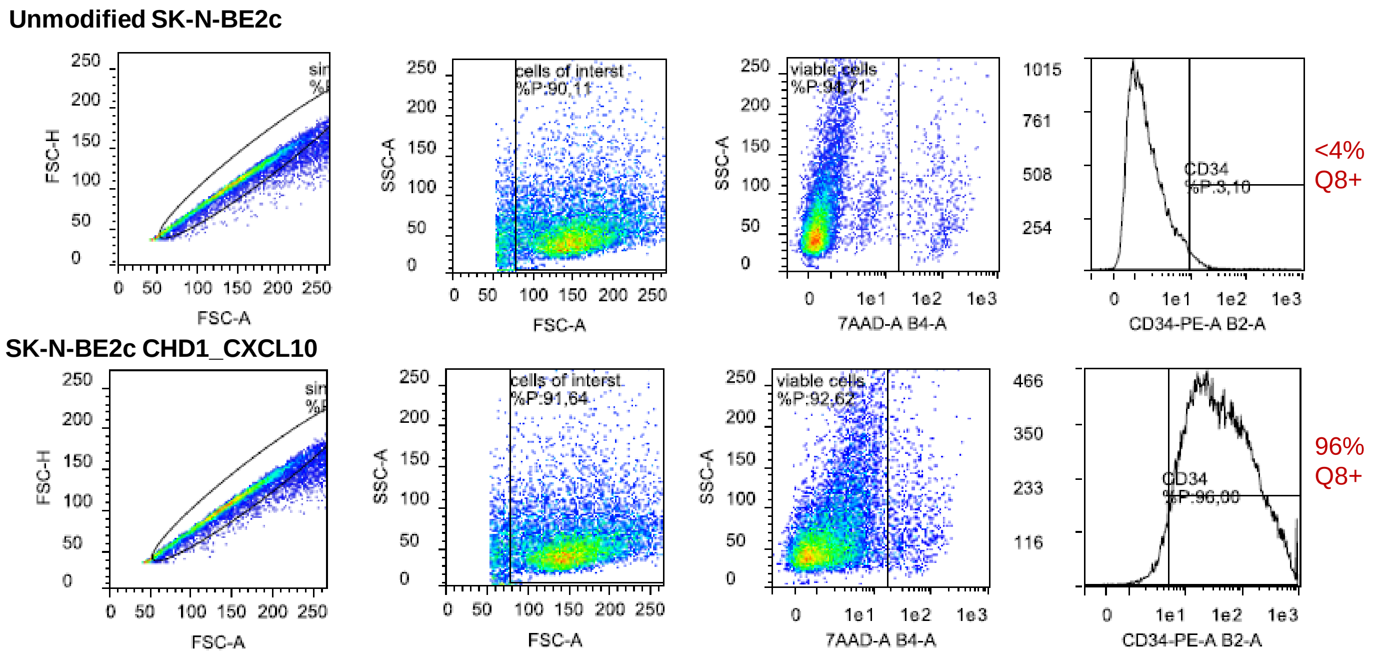

Supplementary Fig. 28. Tumor cell line analysis prior to HuNOG transplantation. Confirmation of transgene expression in SK-N-BE2c neuroblastoma cells. Shown are representative flow cytometry plots of unmodified SK-N-BE2c (Q8$^+$ <4%) and SK-N-BE2c transduced with CHD1_CXCL10 (Q8$^+$ ~96%). Gates indicate viable cells and CD34 (Q8) expression.

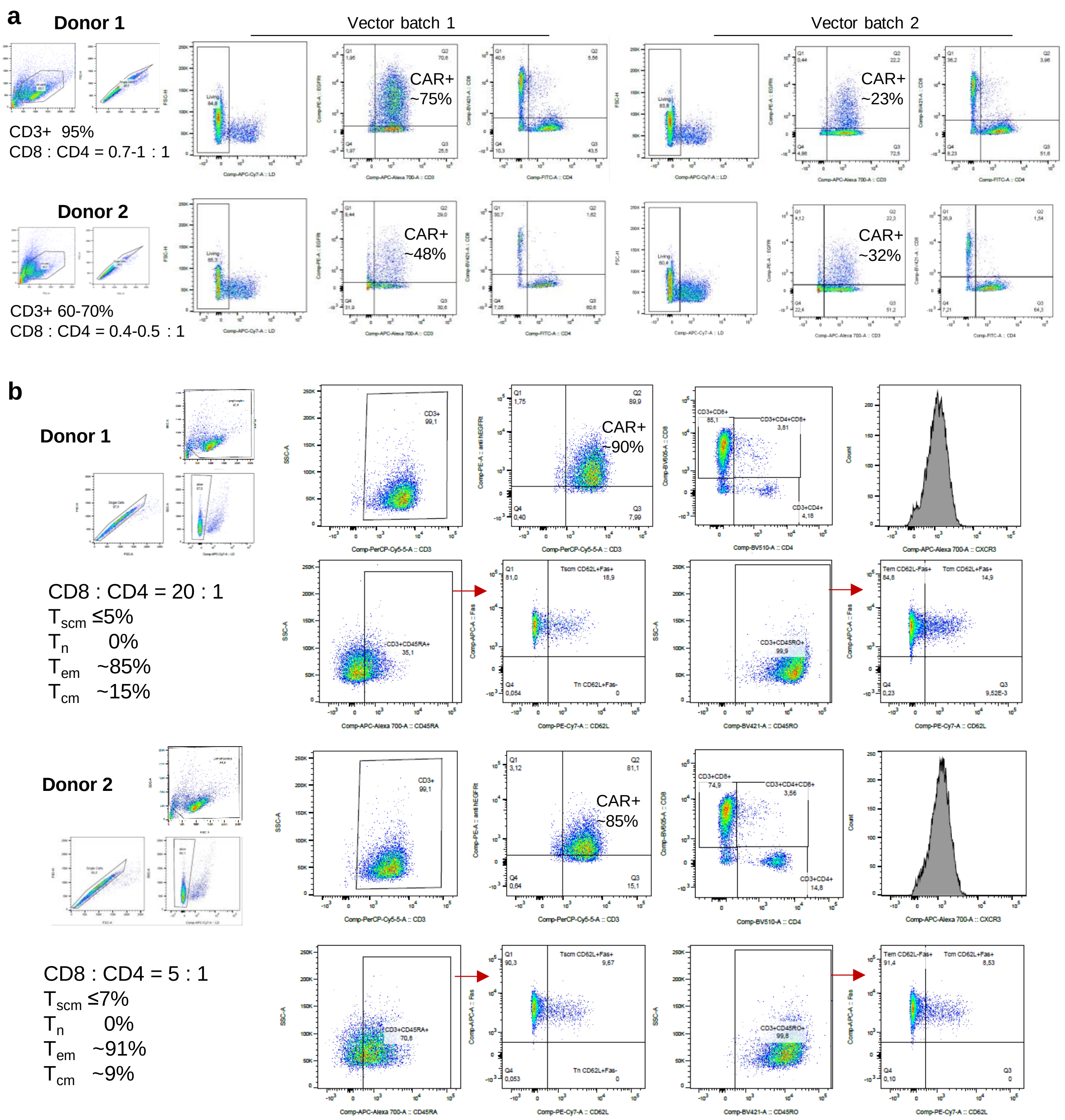

Supplementary Fig. 29. Phenotypic characterization of CAR T cells prior to infusion in HuNOG tumor-bearing mice. (a) Flow cytometry analysis on day 9 after initiation of the rapid expansion protocol, following enrichment for CAR⁺ cells at day 12 post-transduction, from two independent healthy donors using two separate vector batches. Representative gating strategies and CAR expression levels are shown, with frequencies of CAR⁺ T cells indicated. CD3⁺ purity and CD8:CD4 ratios for each donor are provided. (b) Phenotypic composition of CAR T cell products from the same donors. Shown are representative flow cytometry plots with corresponding gating strategies for T cell subsets. Donor 1 and Donor 2 CAR T cell products contained predominantly effector memory T cells (T_{em}), with lower proportions of central memory T cells (T_{cm}) and minimal or absent stem cell memory (T_{scm}) and naïve (T_n) populations. CD8:CD4 ratios and subset distributions are indicated for each donor.

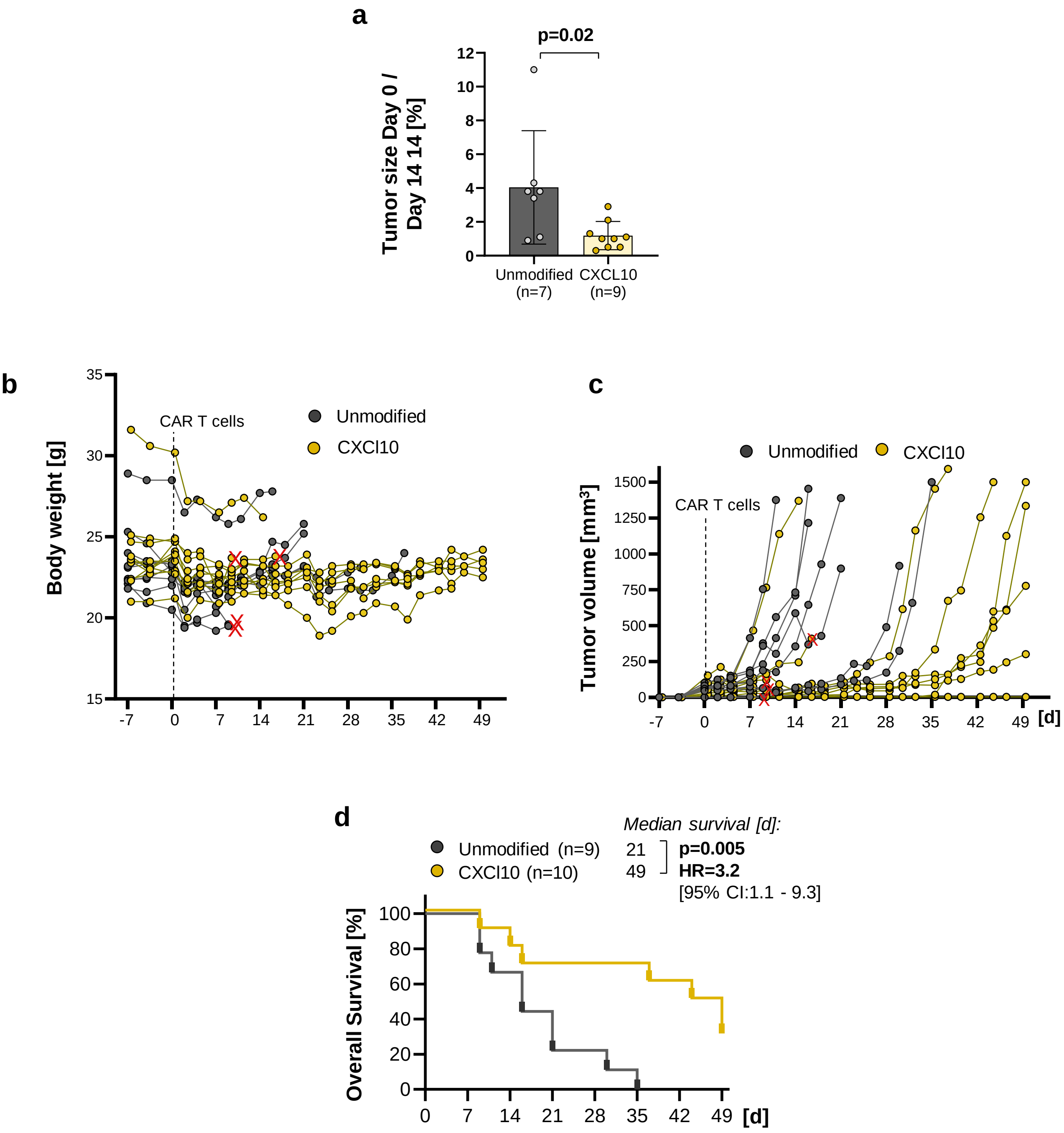

Supplementary Fig. 30. In vivo efficacy of CAR T cells against (CXCL10-expressing) SK-N-BE2c tumors in HuNOG mice. (a) Ratio of tumor volume at day 0 to day 14 after CAR T cell infusion as a measure of early tumor growth. **(b)** Body weight of individual animals over time. Excluded animals are marked with a red X. **(c)** Individual tumor growth curves following CAR T cell treatment. Excluded animals are marked with a red X. **(d)** Kaplan-Meier analysis of overall survival counting excluded animals as non-controlled as an intention to treat (ITT) sensitivity analysis. *N* represents the number of animals at t0. **Data presentation**: (a) Mean ± SD. Dots and *n* represent individual animals. **Statistical analysis**: (a) Two-sided Mann-Whitney test; (d) Two-sided log-rank (Mantel-Cox) test. Source data and exact p values are provided as a Source Data file.

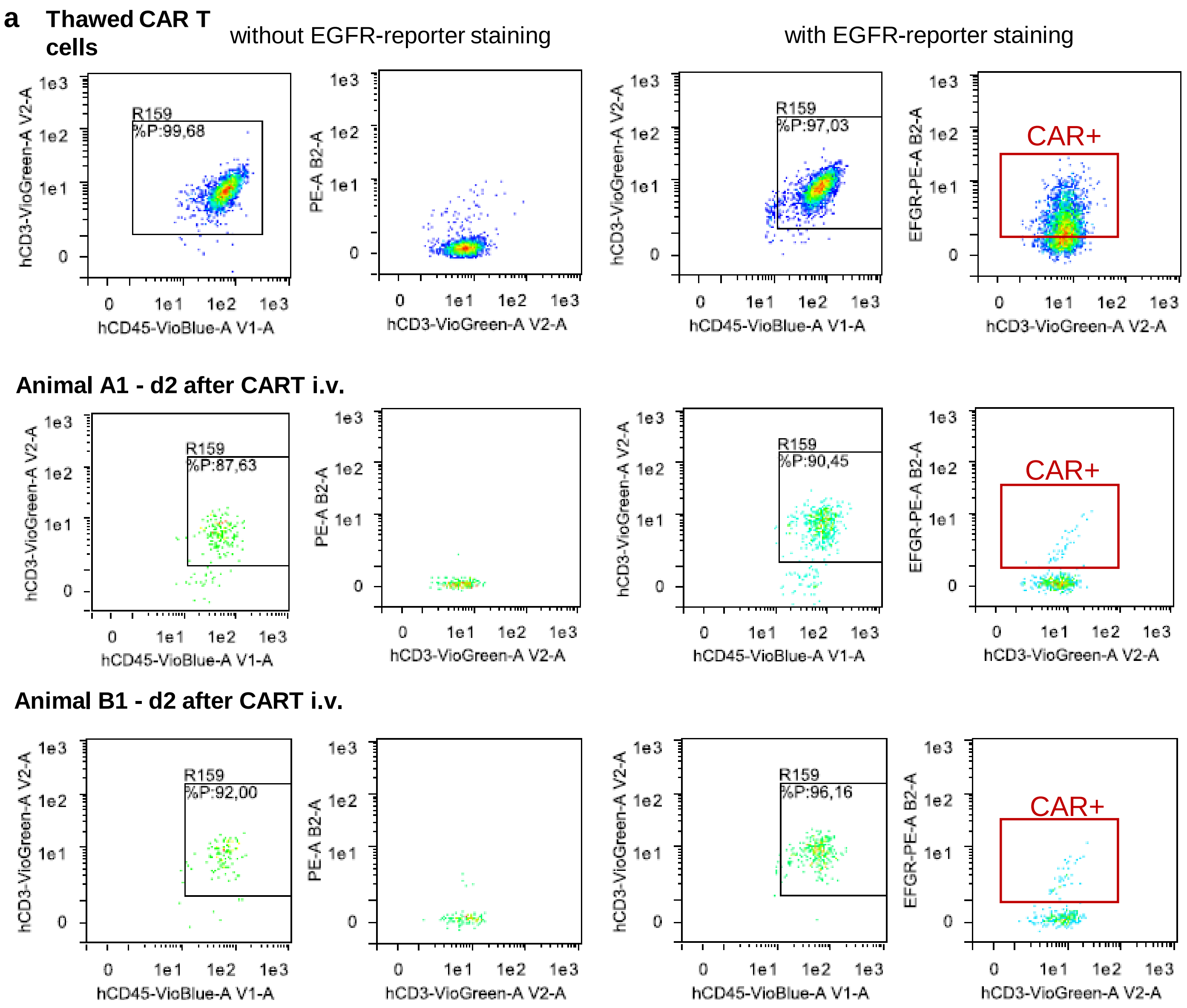

Supplementary Fig. 31. Detection of CAR T cells in vivo by EGFR-reporter staining. Flow cytometry analysis of thawed CAR T cells prior to injection (top row) and peripheral blood mononuclear cells (PBMCs) isolated from mice two days after intravenous infusion of CAR T cells (middle and bottom rows). Representative plots show (from left to right): human CD3^{+}CD45^{+} lymphocyte gating, unstained control, EGFR-reporter staining, and identification of CAR^{+} cells (red gate).

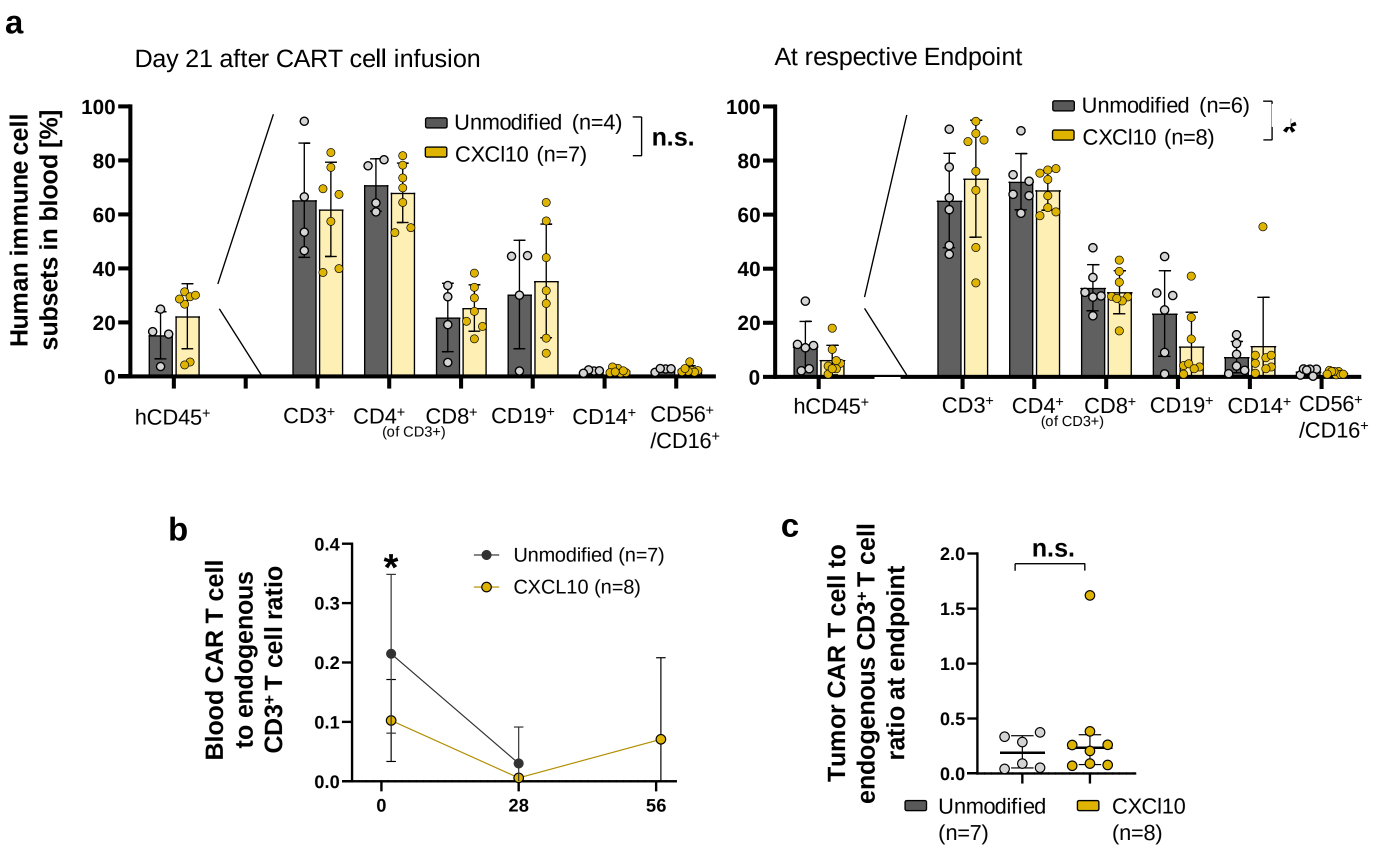

Supplementary Fig. 32 Quantification of human immune and CAR T cell subsets in HuNOG mice. (a) Flow-cytometric quantification of peripheral human immune-cell subsets 21 days after CAR T cell infusion and at the respective experimental endpoints. Human leukocytes are shown as percent of living mononuclear cells; other subsets as percent of human CD45⁺ cells; CD4⁺ and CD8⁺ cells as percent of CD3⁺ cells **(b)** Ratio of circulating CAR T cells (EGFRt⁺) to endogenous human CD3⁺ T cells over time. **(c)** Ratio of tumor-infiltrating CAR T cells (EGFRt⁺) to endogenous human CD3⁺ T cells at endpoint. **Data presentation**: (a-d) Mean ± SD. Dots in (b) represent means, while dots in (a, c) and n in (a-c) represent individual animals. **Statistical analysis**: (c) Two-way ANOVA with Sidaks multiple comparisons test; (c) Mann-Whitney test. *p* values: *< 0.05; *n.s.*, not significant. Source data and exact p values are provided as a Source Data file.

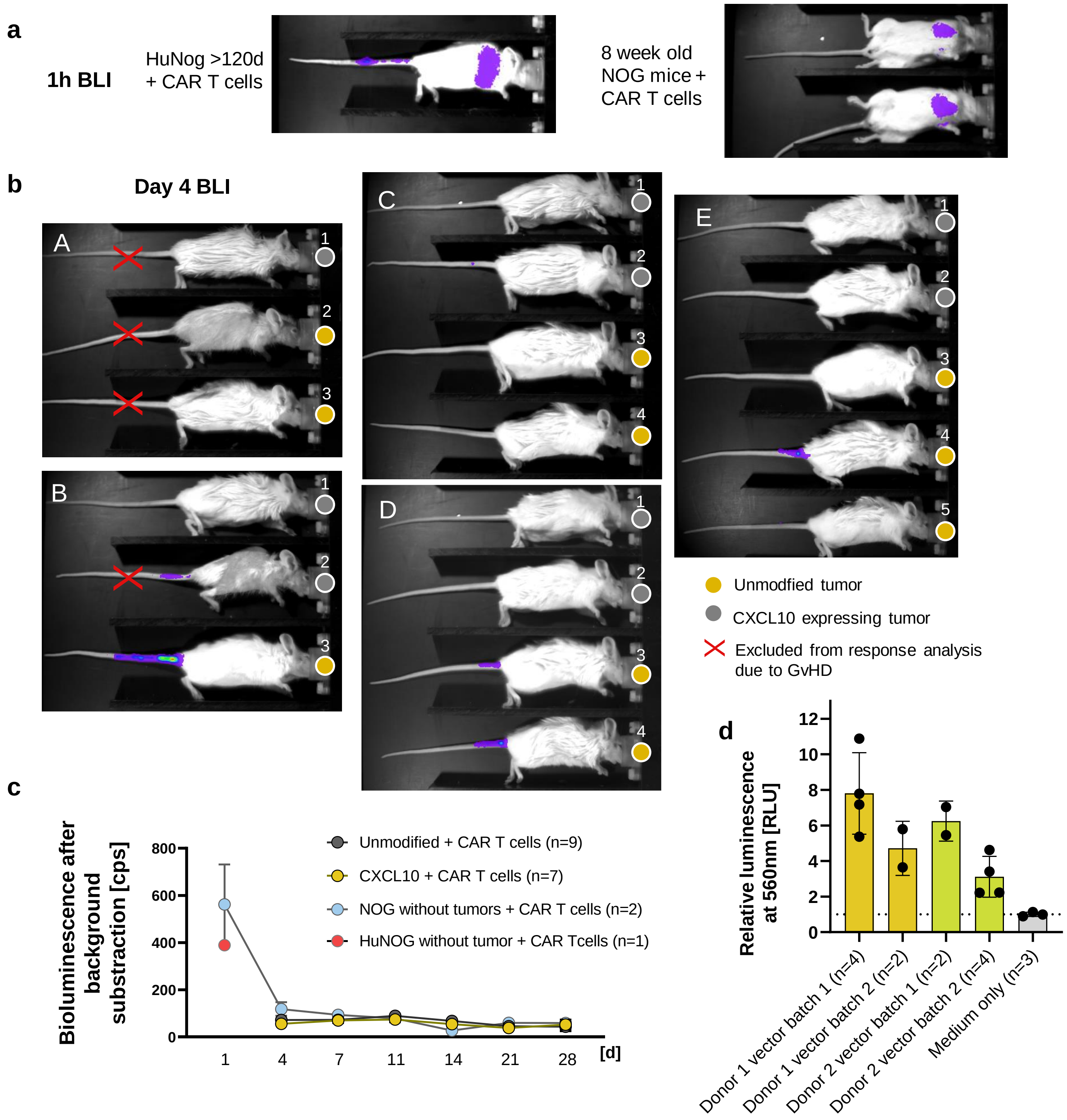

Supplementary Fig. 33. CAR T cell bioluminescence imaging (BLI) in HuNOG mice and functional validation. (a) HuNOG mouse >120 days and two 8-week-old NOG mice without tumor infused with luciferase-expressing L1CAM-targeting CAR T cells and imaged 1 h later to verify BLI signal in the lung. **(b)** Representative BLI images on day 4 after CAR T cell infusion. CXCL10-expressing tumors are indicated by yellow dots, unmodified tumors by grey dots. Mice excluded from response analysis due to GvHD (A1-3) or ectopic tumor (B2) are marked with red crosses. **(c)** Quantification of total bioluminescence over time following CAR T cell injection. Dots represent means, while *n* represents individual animals. **(d)** Functional in vitro validation of CAR T cell luciferase activity prior to in vivo application. Relative luminescence intensity was measured after incubation of luciferase-expressing CAR T cells (derived from two independent donors and two vector batches) with luciferin. Medium-only controls are shown. Dots in and *n* represent biological replicates. **Data presentation**: (c, d) Mean ± SD. **Statistical analysis**: (d) one-way ANOVA with Tukey's post hoc test. *p* values: *< 0.05; *n.s.*, not significant. Source data and exact p values are provided as a Source Data file.

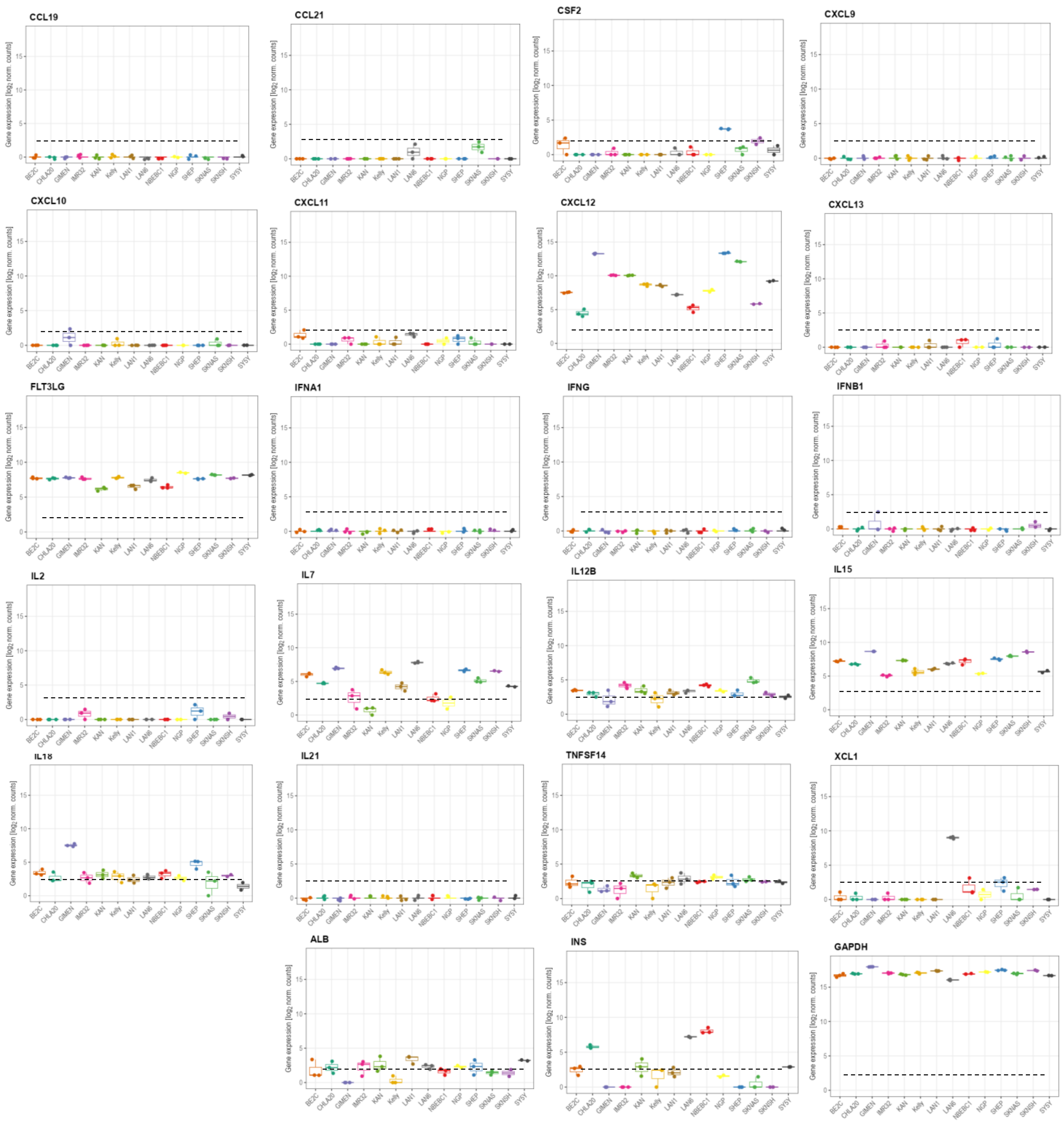

Supplementary Fig. 34. Cytokine expression in neuroblastoma cell lines. Box-and-whisker plots show the distribution of cytokine expression levels stratified by neuroblastoma cell line. Panels depict expression of CCL19, CCL21, CSF2, CXCL9, CXCL10, CXCL11, CXCL12, CXCL13, FLT3LG, IFNA1, IFNG, IL2, IL7, IL12B, IL15, IL18, IL21, TNF, TNFSF14, and XCL1, with ALB and INS included as negative controls, and GAPDH as a positive control. Dotted horizontal lines indicate the estimated mean of ALB and INS expression, serving as a baseline reference for genes with no detectable expression. *Data presentation: median, interquartile range, and overall spread.*

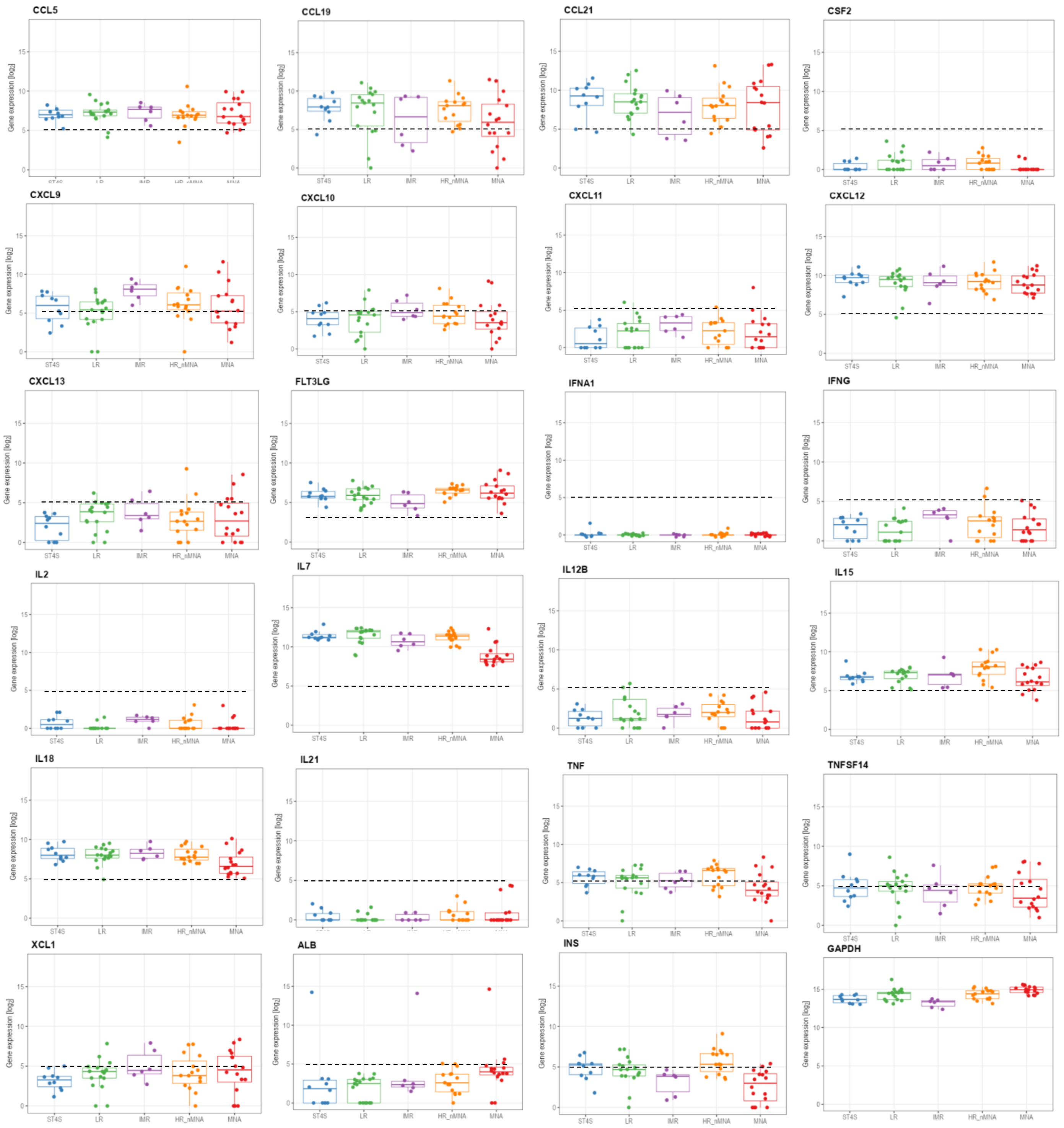

Supplementary Fig. 35. Cytokine expression in patient tumors. Box-and-whisker plots show the distribution of cytokine expression in patient tumor samples stratified by cohort: ST4S (Stage 4S), LR (low risk), HR nMNA (high risk, MYCN non-amplified), and MNA (MYCN amplified). Panels depict expression levels of CCL5, CCL19, CCL21, CSF2, CXCL9, CXCL10, CXCL11, CXCL12, CXCL13, FLT3LG, IFNA1, IFNG, IL2, IL7, IL12B, IL15, IL18, IL21, TNF, TNFSF14, and XCL1, with ALB and INS included as negative controls, and GAPDH as a positive control. Dotted horizontal lines indicate the estimated mean of INS expression, serving as a baseline reference for genes with no detectable expression. *Data presentation: median, interquartile range, and overall spread*

Supplementary Tables

Supplementary Table 1. Exemplary patient data for top and bottom rank novel PAM sites identified via CancerPAM

	Gene	Location	Variant	Expression [log 2 + 1 norm]	gRNA + PAM [5'-3']	CRISPR efficiency (Doench / Moreno Mateos)	CRISPR specificity (CFD / MIT)	rated position	copy number
Patient A								out of 110	
	QKI	Chr6:163575669	T>C	11.1	accagacaccgattgtgtgt AGG	69 / 44	94 / 89	1	2
	SARM1	Chr 17:28392620	A>G	11.9	tggactctcgaggcttgagt CGG	62 / 50	91 / 85	2	5
	PIN1	Chr19:9846446	C>G	9.1	cacaagactgtcaggcggag GGG	62 / 61	86 / 84	3	3
	EMCN	Chr4:100684664	A>C	7.6	cagctttgttctttctactt AGG	33 / 43	27 / 1	96	2
Patient B								out of 289	
	GDAP1L!	Chr20:44250939	T>C	10.5	aggcacgttgtatcaaacat CGG	64 / 62	95 / 91	1	3
	CDYL	Chr6:4844537	A>G	10.7	cacgctgctctgtccgtccg AGG	49 / 50	94 / 88	2	3
	PTCD3	Chr2:86133390	T>C	11.8	tagccgattggccacatcca AGG	49 / 59	93 / 82	3	3
	LINC02253	Chr15:97289166	T>G	0	tattaacttgattttttaaa AGG	19 / 23	23 / 10	263	1

Supplementary Table 2. Neuroblastoma cell line target loci

Gene	Variant	Variant RNA reads	gRNA + PAM [5'-3']	PAM Copy number	Expres-sion [TPM]	depMap CRISPR score	Doench	Moreno	CFD	MIT	CancerPAM Rank
SK-N-AS											out of 406
AP1M1	T>G	59%	CCGCCAGCGCCGTCTACGTG CGG	0.9	12.5	-0.06	67	67	98	96	6
SNX18	C>G	22%	GCGCTACAAGCACTTCGACT GGG	0	9.5	-0.02	52	52	99	97	8
RPLP0	A>G	62%	GCCGTGATGCCCAGGGAAGA CGG	2	16.5	-1,6	47	47	78	35	304
CHST11	T>C	50%	TTGACATCGTCCCCTTTGCG CGG	1	9.5	0	32	32	93	86	34
SK-N-BE2c											out of 426
IGSF9B	C>G	99%	ACGGCAGCCTGACAGTGACA TGG	2.4	14	0.05	37	37	86	69	104
CHD1	A>C	33%	GAATAGTCCGTGGTCTTCCA CGG	1	12.5	0.23	44	44	94	89	36
RBM12	T>G	42%	AATAATCCATCACCCAGTGT AGG	1.2	12.5	-0.33	37	37	89	37	59
SCAF11	G>C	33%	GTAAGTGCCTCTGCCACGGT TGG	0.9	12.5	0.04	60	60	95	60	26
SH3BP1	T>C	45%	GAATTCTTGGTTGCCTGACT GGG	0	9.5	-0.06	45	45	78	45	243

Supplementary Table 3. HDRT & gRNA sequences

high-copy-number pUC-based backbone vector

[INSERT 3‘ end]
GTCATAGCTGTTTCCTGTGTGAAATTGTTATCCGCTCACAATTCCACACAACATACGAGCCGGAAGCATAAAGTGTAAAGCCTGGGGTGCCTAATGAGTGAGCTAACTCACATTA
ATTGCGTTGCGCTCACTGCCCGCTTTCCAGTCGGGAAACCTGTCGTGCCAGCTGCATTAATGAATCGGCCAACGCGCGGGGAGAGGCGGTTTGCGTATTGGGCGCTCTTCCGCT
TCCTCGCTCACTGACTCGCTGCGCTCGGTCGTTCGGCTGCGGCGAGCGGTATCAGCTCACTCAAAGGCGGTAATACGGTTATCCACAGAATCAGGGGATAACGCAGGAAAGAA
CATGTGAGCAAAAGGCCAGCAAAAGGCCAGGAACCGTAAAAAGGCCGCGTTGCTGGCGTTTTTCCATAGGCTCCGCCCCCCTGACGAGCATCACAAAAATCGACGCTCAAGTC
AGAGGTGGCGAAACCCGACAGGACTATAAAGATACCAGGCGTTTCCCCCTGGAAGCTCCCTCGTGCGCTCTCCTGTTCCGACCCTGCCGCTTACCGGATACCTGTCCGCCTTTCT
CCCTTCGGGAAGCGTGGCGCTTTCTCATAGCTCACGCTGTAGGTATCTCAGTTCGGTGTAGGTCGTTCGCTCCAAGCTGGGCTGTGTGCACGAACCCCCCGTTCAGCCCGACCG
CTGCGCCTTATCCGGTAACTATCGTCTTGAGTCCAACCCGGTAAGACACGACTTATCGCCACTGGCAGCAGCCACTGGTAACAGGATTAGCAGAGCGAGGTATGTAGGCGGTGC
TACAGAGTTCTTGAAGTGGTGGCCTAACTACGGCTACACTAGAAGAACAGTATTTGGTATCTGCGCTCTGCTGAAGCCAGTTACCTTCGGAAAAAGAGTTGGTAGCTCTTGATCC
GGCAAACAAACCACCGCTGGTAGCGGTGGTTTTTTTGTTTGCAAGCAGCAGATTACGCGCAGAAAAAAAGGATCTCAAGAAGATCCTTTGATCTTTTCTACGGGGTCTGACGCTC
AGTGGAACGAAAACTCACGTTAAGGGATTTTGGTCATGAGATTATCAAAAAGGATCTTCACCTAGATCCTTTTAAATTAAAAATGAAGTTTTAAATCAATCTAAAGTATATATGAG
TAAACTTGGTCTGACAGTTACCAATGCTTAATCAGTGAGGCACCTATCTCAGCGATCTGTCTATTTCGTTCATCCATAGTTGCCTGACTCCCCGTCGTGTAGATAACTACGATACG
GGAGGGCTTACCATCTGGCCCCAGTGCTGCAATGATACCGCGAGACCCACGCTCACCGGCTCCAGATTTATCAGCAATAAACCAGCCAGCCGGAAGGGCCGAGCGCAGAAGTG
GTCCTGCAACTTTATCCGCCTCCATCCAGTCTATTAATTGTTGCCGGGAAGCTAGAGTAAGTAGTTCGCCAGTTAATAGTTTGCGCAACGTTGTTGCCATTGCTACAGGCATCGTG
GTGTCACGCTCGTCGTTTGGTATGGCTTCATTCAGCTCCGGTTCCCAACGATCAAGGCGAGTTACATGATCCCCCATGTTGTGCAAAAAAGCGGTTAGCTCCTTCGGTCCTCCGAT
CGTTGTCAGAAGTAAGTTGGCCGCAGTGTTATCACTCATGGTTATGGCAGCACTGCATAATTCTCTTACTGTCATGCCATCCGTAAGATGCTTTTCTGTGACTGGTGAGTACTCAA
CCAAGTCATTCTGAGAATAGTGTATGCGGCGACCGAGTTGCTCTTGCCCGGCGTCAATACGGGATAATACCGCGCCACATAGCAGAACTTTAAAAGTGCTCATCATTGGAAAAC
GTTCTTCGGGGCGAAAACTCTCAAGGATCTTACCGCTGTTGAGATCCAGTTCGATGTAACCCACTCGTGCACCCAACTGATCTTCAGCATCTTTTACTTTCACCAGCGTTTCTGGG
TGAGCAAAAACAGGAAGGCAAAATGCCGCAAAAAAGGGAATAAGGGCGACACGGAAATGTTGAATACTCATACTCTTCCTTTTTCAATATTATTGAAGCATTTATCAGGGTTATT
GTCTCATGAGCGGATACATATTTGAATGTATTTAGAAAAATAAACAAATAGGGGTTCCGCGCACATTTCCCCGAAAAGTGCCACCTGACGTCTAAGAAACCATTATTATCATGAC
ATTAACCTATAAAAATAGGCGTATCACGAGGCCCTTTCGTCTCGCGCGTTTCGGTGATGACGGTGAAAACCTCTGACACATGCAGCTCCCGGAGACGGTCACAGCTTGTCTGTAA
GCGGATGCCGGGAGCAGACAAGCCCGTCAGGGCGCGTCAGCGGGTGTTGGCGGGTGTCGGGGCTGGCTTAACTATGCGGCATCAGAGCAGATTGTACTGAGAGTGCACCAT
ATGCGGTGTGAAATACCGCACAGATGCGTAAGGAGAAAATACCGCATCAGGCGCCATTCGCCATTCAGGCTGCGCAACTGTTGGGAAGGGCGATCGGTGCGGGCCTCTTCGCT
ATTACGCCAGCTGGCGAAAGGGGGATGTGCTGCAAGGCGATTAAGTTGGGTAACGCCAGGGTTTTCCCAGTCACGACGTTGTAAAACGACGGCCAGT
[Insert 5‘ end]

AAVS1 5‘homology arm (399bp)

ACCGTTTTTCTGGACAACCCCAAAGTACCCCGTCTCCCTGGCTTTAGCCACCTCTCCATCCTCTTGCTTTCTTTGCCTGGACACCCCGTTCTCCTGTGGATTCGGGTCACCTCTCACT
CCTTTCATTTGGGCAGCTCCCCTACCCCCCTTACCTCTCTAGTCTGTGCTAGCTCTTCCAGCCCCCTGTCATGGCATCTTCCAGGGGTCCGAGAGCTCAGCTAGTCTTCTTCCTCCA
ACCCGGGCCCCTATGTCCACTTCAGGACAGCATGTTTGCTGCCTCCAGGGATCCTGTGTCCCCGAGCTGGGACCACCTTATATTCCCAGGGCCGGTTAATGTGGCTCTGGTTCTG
GGTACTTTTATCTGTCCCCTCCACCCCACAGTGGGGCCACTAGGGACAG

AAVS1 3’homology arm (400bp)

GATTGGTGACAGAAAAGCCCCATCCTTAGGCCTCCTCCTTCCTAGTCTCCTGATATTGGGTCTAACCCCCACCTCCTGTTAGGCAGATTCCTTATCTGGTGACACACCCCCATTTCC
TGGAGCCATCTCTCTCCTTGCCAGAACCTCTAAGGTTTGCTTACGATGGAGCCAGAGAGGATCCTGGGAGGGAGAGCTTGGCAGGGGGTGGGAGGGAAGGGGGGGATGCGT
GACCTGCCCGGTTCTCAGTGGCCACCCTGCGCTACCCTCTCCCAGAACCTGAGCTGCTCTGACGCGGCCGTCTGGTGCGTTTCACTGATCCTGGTGCTGCAGCTTCCTTACACTTC
CCAAGAGGAGAAGCAGTTTGGAAAAACAAAATCAGAATAAGTTGGTCCTGAGTTCT

AP1M1 5’homology arm (400bp)

GTCGGTTTCACCACCAGGCTCTCTCGCCTAACTCGGCCCACGGGAGGCCGTTTCCAGCTGCTCTGGCTCCATAGCTCAGCCTGGCAGCGGCCACTGGGTGCCCTGATTTGGTCG
GCCCCGCCCCACCGGGCCTTACTTCCGGGATGCTTCTGGCGGGAGCAAGAAACATGGTTACATACGACGTAATGCGCCTGCGCACAGTCCGTCCTGCGCCTGCGCATTCCTGAC
AGCACCTCCCGGACCGGACATAACGGTCCCCGCGCGGCTCCCCGAACCGGAAGTGGAGGTGAGCTGTCGCGGGCGGCGCCCGGCCTTGCTCAACGCCCAGCAGTCCCCACCG
TCGCTGCCGACGCCACCGCCCTCGACCGCTGCCGAGGCCTCCTGCAGCCATCATGTCCGC

AP1M1 3’homology arm (400bp)

GACCTGAAGGGCAAGGTACTGAGGGCTCCCCACCCTCCCTGTTGCCAGGCAACCGGCAGGGGCCTCCGCCCGCGGGGACCCACCCTGTTGCTAGGTAACCGGCTTATGAGCCC
CATCCTGGTGTGAGGCAGAGAGGCCGGTAGACCTCACCTGAGAGCCCCATCCTGTTTCTGGGTAACCGGGCGGGGGTCTTAAGTGGGTAATCCCCGCCCTGTTGCTACGTAAC
GCGGTGGGCTGCAGCCACCGCGTTGCCAGGTAACTGACTGCACCGCCCTCTGCTCCCCAGCGCCGGGGCCAGGGGCTCGGGCCTCGGTGGAGGACCCAGAAGCGGTGCCGCA
GCCGGCCTCGGAGCCAGGCAGGGCGTCCAGGGGCTGCGCGACCCTTGGCCGGGGGTGGCGAG

SNX18 5’homology arm (400bp)

CTAGATCGACGGCTCGTCTTCGGCGGGTGTGGGCGCAGCCGGCCGCTACCGCCTGTCCACGCGCTCCGACCTGTCCCTGGGTTCCCGCGGCGGCTCGGTCCCCCCGCAGCACC
ACCCGTCGGGGCCCAAGAGCTCGGCCACCGTGAGCCGCAACCTCAATCGCTTCTCCACCTTCGTCAAGTCCGGCGGGGAGGCCTTCGTGCTGGGGGAGGCGTCAGGCTTCGTG
AAGGACGGGGACAAGCTGTGCGTGGTGCTGGGGCCCTATGGCCCCGAGTGGCAGGAGAACCCCTACCCGTTCCAGTGCACCATCGACGACCCCACCAAGCAGACCAAGTTCA
AGGGCATGAAGAGCTACATCTCCTACAAGCTGGTGCCCACGCACACGCAGGTGCCGGTGCAT

SNX18 3’homology arm (395bp)

TGTACGCGCGCCTGGCGGAGAAGTTCCCGGTCATCTCCGTGCCCCACCTGCCCGAGAAGCAGGCCACCGGCCGCTTCGAGGAGGACTTCATCTCTAAGCGCAGGAAGGGCCTG
ATCTGGTGGATGAACCACATGGCCAGCCACCCAGTGCTGGCGCAGTGCGACGTCTTCCAGCACTTCCTGACGTGCCCCAGCAGCACCGACGAGAAAGCCTGGAAGCAGGGCAA
GAGGAAGGCCGAGAAGGACGAGATGGTGGGCGCCAACTTCTTCCTGACCCTTAGCACGCCCCCCGCCGCTGCCCTTGACCTGCAGGAGGTGGAGAGCAAGATCGACGGCTTC
AAGTGCTTCACCAAGAAGATGGACGACAGCGCGCTGCAGCTCAACCACACGGCCAAC

RPLP0 5’homology arm (400bp)

ACAGGAGCGCTATCCGCGGTTTCTGATTGGCTACTTTGTTCGCATTATAAAAGGCACGCGCGGGCGCGAGGCCCTTCTCTCGCCAGGCGTCCTCGTGGAAGGTTCGTGTGCTAG
TTAGATGGGCGCCAGGGGTCGCCGGCGGGAAGCATGGAGGGGTCTTTGGGGGCCTTTGGGAACATGGAGTCCTATTCTGTTCCGCCTGGGGCCTCGGTGGCGGCTTGCACGC
CCCGAGATGACGGCCGCTGCCCTAGGCAGGGCCGGCGGGCGATTGCGCGTGTCCTGCTCCTCTTAGGCCCGGGACCGCGGGATGGGTGTCGGCGTGACCAGGCCTGAGCTCC
CTGTCTCTCCTCAGTGACATCGTCTTTAAACCCTGCGTGGCAATCCCTGACGCACCGCCGTG

RPLP0 3’homology arm (400bp)

GGCGACCTGGAAGTCCAACTACTTCCTTAAGATCATCGTAAGTGCAGGGTGGGTCGCCTCTGCTCTTCATGTTGCCCCAGCGCAAATAGGGACAGTCAGCTGCTATGTGCTGAG
GGTCTACTCACACCGGCTACTGAATTAGGCCATTTTTGGGAAAATACTGTTTAGTTAACAATTTCCTGAGATAGGTCCCTTCTGTTGCAGATAAACGGGCTCAGGCAAGTTAAGT
GGGTCCTAAGATGACAGCATTCGTATCCAGGTCTGTCTGGCTTCTAAAAGAGCGCGCTTTATACTTTTTATTTTTTAAACGGAGTCTCGCTCTGTTGTCCAGGCTGGAGTGCAGTG
GCTTGATCTTGGCTTACTGCAACCTCCGCTTCCCAGGTTCAAGCGATTCTCCTGC

CHST11 5’homology arm (400bp)

AAGCCGTAAGCGGAGGGTGCTGACCCCCAACGACCTGAAGCACTTGGTGGTGGATGAGGACCACGAGCTCATCTACTGCTACGTGCCCAAGGTGGCCTGCACCAACTGGAAGC
GGCTCATGATGGTCCTGACCGGGCGGGGGAAGTACAGCGACCCCATGGAGATCCCGGCCAACGAGGCACACGTCTCCGCCAACCTGAAGACCCTGAACCAGTACAGCATCCCA
GAAATCAACCACCGCTTGAAAAGCTACATGAAGTTCCTGTTTGTCCGGGAGCCCTTCGAGAGGCTAGTGTCCGCCTACCGCAACAAGTTCACCCAGAAGTACAACATCTCCTTCC
ACAAGCGGTACGGCACCAAGATCATCAAACGCCAGCGGAAGAACGCCACCCAGGAGGCC

CHST11 3’homology arm (400bp)

ACGATGTCAAATTCGAGGAGTTTGTGGCCTATCTCATCGACCCACACACCCAGCGGGAGGAGCCTTTCAACGAACACTGGCAAACCGTCTACTCACTCTGCCATCCCTGCCACAT
CCACTATGACCTCGTGGGCAAGTACGAGACACTGGAAGAGGATTCTAATTACGTCCTGCAGCTGGCAGGAGTGGGCAGCTACCTGAAGTTCCCCACCTATGCAAAGTCTACGAG

AACTACTGATGAAATGACCACAGAATTCTTCCAGAACATCAGCTCAGAGCACCAAACGCAGCTGTACGAAGTCTACAAACTCGATTTTTTAATGTTCAATTACTCAGTGCCAAGCT
ACCTGAAATTGGAATAAAGGGGGTGGGGAGAGGGAGAGAATCATGCTTTTTAATT

IGSF9b 5' homology arm (400bp)

AAGCCCATTGTCACCTGGCTCAAGGAGGGGACGCTCCTCGGTGCTAGTGGGAAATACCAGGTGAGTGTGGTTCTAGGTTGGGGCCGCTGCGGTCTCTTCCCCCGTTTCCCCAG
GGCAGCCTCCTACTGCAGCCGACCCCTGATGCTACAGGCACAAGCACAGAGGAGGCTGGAAAGCCGTAGCCTGGCTCAGCTGCAGGCGTCTCTGTGTCGCTGGCGGGAGATG
GGCAAGGGGGCACCTGCCCTTCGTGTCCCCCAGATGCATCTGTCCCTGTGGTCATCTCCACCTTAGTGTCTCCGACTGCATCTCCGTGTTTGAGGGTAGCAGCGGTGGGTGTGA
GCGTGGGTGCTGAGCTCCCTCTGTTTTGGGCTCCCAGGTGAGTGACGGCAGCCTGACAGTG

IGSF9b 3' homology arm (400bp)

GTCAGTCGGGAGGACAGAGGTGCCTACACCTGCCGAGCGTACAGCATTCAGGGGGAGGCTGTCCACACGACTCACCTGCTTGTCCAAGGTAGGAGCCATTTCCTCAGCCTCTTT
AACCCTGGGCTCCCGCGGGGATGTCCAGCCCGGCTGAGCTACGGAGGGCGTTTTCCCAGGGCTCAGCTGGGGCTAGTGGAGGTGCAGGCGGCCATGAGGACCTGTGCGGGG
CTCCTGTGTGCTGCCTGCTGCCCTGGTCAGAGCTCCTGCACCGGCTGCCTGGGAGAGGGCCAGGCCCACAGGCCGTGGCCAGGCACCACATGGCGTGGCAGAGAGGCCCCTG
CTGTGTGGGTTTCTGGTTTCTCCTTCTGTTCAGATAGCATCTCTCCCATGTCTCCGCCTCCCT

CHD1 5'homology arm (400bp)

TTATTGATCAATGTTTCTTCACAGTTCTGATCTCCTCAGGAAAAATGCCTAGAGGTTATGTTGCTGCGTCAGTAGAATAACTTTTGGAGGCTTTTGATATAATGTCTGATTACCTTT
TAAATCAAAATATCCACCAGAGGTATATATTTTTCTCTGTTTGCTCTTTAATTTTGTTTATGGAATGTTTTTGATACATTTAATAACATTTTTAAGATTCCGACCATCTTTTATAATGG
TTTTTGCTCTTAAGATACAAGCTGTTTTTACACTAAGGTTGGGTAAATATTTTCCAATTCCAGATTAGTTTCAATGGAAGTGAAGGGAGGCGCAGTAGAAGTAGGAGATACTCTG
GATCTGATAGTGATTCCATCTCAGAAGGGAAAAGGCCAAAGAACCGTGG

CHD1 3'homology arm (400bp)

AAGACCACGGACTATTCCTCGGGAGAATATTAAAGGATTTAGTGATGCAGAAATTAGGCGGTAAGGTGTTATAAGACAATCGCTTCAACTTGGTTTTAGTAGAGTGTGAAACTA
AAATGTAAAAACGCCGTAATTTTCAAAAATTGTGATGAAGTTTTAGAAGAGGCAAATCTGTTATTTGAGTAGGAATTGGGTGGTTCTGCTTTTGGGTATTTGTTATAACTGAACAA
AACAAGTTTGGTGTCTAATCTTTGCATGTTATTTATTTATCGCTAAACTGCTTAGACTACACAATTTTTTTTATTAATTTGACTCAATAGTGATCCTTTCGAATAATAAAAGTAGTTTT
GATGCATTTGATTTTTAAAAATTCTCACGGTGTTTATGACTCCCAAAGTTA

RBM12 5'homology arm (400bp)

TGTGGTTTCAGCGCAGCATGGCTGTGGTCATCCGTTTGCAAGGTCTCCCAATTGTGGCGGGGACCATGGACATTCGCCACTTCTTCTCTGGATTGACCATTCCTGATGGGGGCGT
GCATATTGTAGGGGGTGAACTGGGTGAGGCTTTCATCGTTTTTGCCACTGATGAAGATGCAAGGCTTGGTATGATGCGCACAGGTGGTACAATTAAAGGGTCAAAAGTAACACT
ATTGTTGAGTAGTAAGACGGAAATGCAGAATATGATTGAACTGAGTCGTAGGCGTTTTGAAACTGCCAACTTAGATATACCACCAGCAAATGCCAGTAGATCAGGACCACCACC
TAGCTCAGGAATGAGTAGCAGGGTAAACTTGCCCACAACAGTATCCAACTTTAATAA

RBM12 3'homology arm (400bp)

TACTGCCACCACTTCTGTTCATGAAAGCAACAAAAACATACAGACATTTTCCACAGCCAGCGTAGGAACAGCTCCTCCAAATATGGGGGCTTCCTTTGGGAGCCCAACGTTTAGC
TCAACTGTTCCAAGCACAGCCTCTCCAATGAACACAGTCCCGCCGCCACCAATTCCTCCAATTCCAGCGATGCCATCTCTGCCACCAATGCCATCCATTCCCCCAATTCCAGTTCCT
CCTCCAGTACCTACATTGCCTCCTGTGCCTCCTGTGCCCCCGATTCCCCCAGTTCCTTCTGTGCCACCCATGACCCCACTGCCACCCATGTCGGGCATGCCGCCCTTGAATCCGCCA
CCTGTGGCACCTCTACCTGCTGGAATGAATGGCTCTGGAGCACCTATGAAT

SCAF11 5'homology arm (401bp)

TGTAGAACAAAAAGTAAGAGTTCATCATTTGGTAGAATTGACAGAGATAGTTACTCTCCCCGGTGGAAGGGAAGATGGGCAAATGATGGTTGGAGATGTCCACGAGGAAATGA
TCGGTACAGAAAGAATGACCCAGAGAAACAGAATGAAAATACAAGAAAAGAAAAAAATGACATCCATCTAGATGCTGATGATCCAAATTCTGCTGACAAACATAGAAATGACTG
TCCCAATTGGATAACAGAAAAAATAAACTCTGGGCCTGATCCAAGAACCAGAAATCCAGAAAAGTTGAAAGAGTCTCATTGGGAAGAAAATAGAAATGAAAATTCAGGAAATTC
TTGGAATAAAAACTTTGGTTCTGGTTGGGTATCTAACCGTGGTAGAGGCAGAGCCAACCG

SCAF11 3'homology arm (400bp)

GTGGCAGAGGCACTTACAGAAGTAGTTTTGCCTATAAAGATCAGAATGAAAATCGGTGGCAAAATCGAAAACCCCTCTCAGGGAATTCAAACAGTTCAGGGAGTGAATCTTTCA
AGTTTGTGGAACAGCAATCCTATAAGCGAAAAAGTGAACAGGAGTTCTCATTTGATACACCAGCAGATAGATCTGGATGGACATCTGCATCCAGCTGGGCCGTGAGAAAGACTT
TGCCAGCAGATGTACAAAACTACTACTCACGACGAGGCAGAAATTCTTCAGGTCCACAGTCTGGATGGATGAAACAAGAGGAGGAAACATCTGGACAGGGTAATGTATTCTTGT
TGGTTTATTGATACTGTTTTATTTGAAGAATTATAAATTTAATATGTTATTTGTATGA

SH3BP1 5'homology arm (400bp)

CCACACCAGGAGGATGCTGTGGGGTCTGCTGAGTACAGTGTGTGTACTTGTGCACGCCTGGTCCCTAGGAGCACAGGTGGTTAGCGTATCTGTGAGGGTGTGCCCGCGTGTGT
CACAGCTGGTGCTGGGGCAACCCTAAAGCCTGGGATCCTGTAAACAGTCCTGCGGCAGCCCTGTTCTCCCCTCTGTGGAGTGGCTGTGATCACCAAATCTGCCACCAGGGGGCA
ACCCAGAGCACTCATGGCCCAGCCTGGGCAGAGGCCCCGGCCAGTGGAGCTCTCAGATCTGCAGCTCTAAATCCTAGAATCCCATGTGGTCTGCTGTATGGAGGGGACACTTG
GCTCTGGACATGCAACAGGGCAGGTGACCAGCTCACCTTGGGATCATCTCCAGGCCCAGT

SH3BP1 3'homology arm (400bp)

CAGGCAACCAAGAATTCAGGCAGCAGTCAAGGCCTAGGAGGCAGCCCGGGTAGTCACAGCCATACGACCATGGCCAACAAGGTGGAGACGCTGAAGGAGGAGGAGGAGGAG
CTGAAGAGGAAAGTGGAGCAATGCAGGGTGAGGGCCATGGGGGTCCCCTGGATATGTAGGGGTGGCAGGGGTGGGTCCTGGCCCATCTCACAGGCAAGGAAGCTGAAGTTC
AGAGAGGTGACATGACTTGCCTAAGGCCACGTGGCTAGGAGCCAGCAGAGCTCCGCCATTCCCTCGTAGCCTCTTCTGTCTGTACCCCATGCATAACTTGGGAAGGCAGAGACT
TGGTCTGTTCCCTGAGTCTCCATGTGTTTGTTTGCTGAGCGAGCGAGTGAATGAATAAATGAAG

Ef1a(s) promoter 239bp

GCTCCGGTGCCCGTCAGTGGGCAGAGCGCACATCGCCCACAGTCCCCGAGAAGTTGGGGGGAGGGGTCGGCAATTGAACCGGTGCCTAGAGAAGGTGGCGCGGGGTAAACT
GGGAAAGTGATGTCGTGTACTGGCTCCGCCTTTTTCCCGAGGGTGGGGGAGAACCGTATATAAGTGCAGTAGTCGCCGTGAACGTTCTTTTTCGCAACGGGTTTGCCGCCAGAA
CACAGGCCGCCACC

CXCL10 (294bp)

ATGAACCAGACCGCCATCCTGATCTGCTGCCTGATCTTCCTGACCCTGAGCGGCATCCAGGGAGTCCCCCTGTCCAGAACAGTGCGGTGCACCTGTATCAGCATCTCTAATCAAC
CTGTGAACCCAAGAAGCCTGGAAAAGCTCGAGATCATCCCTGCTTCTCAGTTCTGCCCTAGAGTGGAAATCATCGCCACAATGAAAAAGAAGGGCGAGAAGAGATGTCTGAACC
CCGAGAGCAAGGCCATTAAGAACCTGCTGAAGGCCGTGTCCAAAGAGATGAGCAAGCGGAGCCCT

CXCL11 (282bp)

ATGAGTGTGAAGGGCATGGCTATAGCCTTGGCTGTGATATTGTGTGCTACAGTTGTTCAAGGCTTCCCCATGTTCAAAAGAGGACGCTGTCTTTGCATAGGCCCTGGGGTAAAA
GCAGTGAAAGTGGCAGATATTGAGAAAGCCTCCATAATGTACCCAAGTAACAACTGTGACAAAATAGAAGTGATTATTACCCTGAAAGAAAATAAAGGACAACGATGCCTAAAT
CCCAAATCGAAGCAAGCAAGGCTTATAATCAAAAAAGTTGAAAGAAAGAATTTT

IFNG (498bp)

ATGAAGTACACCTCTTACATTCTGGCATTCCAATTGTGCATTGTGCTGGGAAGCCTCGGGTGCTATTGTCAAGATCCCTACGTGAAAGAGGCTGAGAATCTGAAGAAGTACTTCA
ACGCCGGGCACAGTGACGTCGCTGACAACGGTACACTGTTCTTGGGAATCTTAAAGAACTGGAAGGAAGAAAGCGATCGAAAGATCATGCAATCCCAGATCGTGAGCTTCTATT
TTAAGCTGTTCAAGAACTTCAAGGACGACCAATCTATACAGAAATCAGTCGAGACAATCAAAGAGGATATGAACGTGAAGTTCTTTAACTCTAATAAGAAGAAGAGGGACGATT
TTGAGAAACTTACAAACTACAGCGTCACGGATCTCAACGTACAGCGGAAGGCGATCCACGAGCTTATTCAGGTCATGGCCGAGTTGTCCCCGGCTGCGAAGACTGGCAAACGG
AAGCGTTCACAAATGCTTTTCAGAGGGAGACGAGCGTCACAA

GFP (714bp)

GTGAGCAAGGGCGAGGAGCTGTTCACCGGGGTGGTGCCCATCCTGGTCGAGCTGGACGGCGACGTAAACGGCCACAAGTTCAGCGTGTCCGGCGAGGGCGAGGGCGATGCC
ACCTACGGCAAGCTGACCCTGAAGTTCATCTGCACCACCGGCAAGCTGCCCGTGCCCTGGCCCACCCTCGTGACCACCCTGACCTACGGCGTGCAGTGCTTCAGCCGCTACCCC
GACCACATGAAGCAGCACGACTTCTTCAAGTCCGCCATGCCCGAAGGCTACGTCCAGGAGCGCACCATCTTCTTCAAGGACGACGGCAACTACAAGACCCGCGCCGAGGTGAA
GTTCGAGGGCGACACCCTGGTGAACCGCATCGAGCTGAAGGGCATCGACTTCAAGGAGGACGGCAACATCCTGGGGCACAAGCTGGAGTACAACTACAACAGCCACAACGTC
TATATCATGGCCGACAAGCAGAAGAACGGCATCAAGGTGAACTTCAAGATCCGCCACAACATCGAGGACGGCAGCGTGCAGCTCGCCGACCACTACCAGCAGAACACCCCCAT

CGGCGACGGCCCCGTGCTGCTGCCCGACAACCACTACCTGAGCACCCAGTCCGCCCTGAGCAAAGACCCCAACGAGAAGCGCGATCACATGGTCCTGCTGGAGTTCGTGACCG CCGCCGGGATCACTCTCGGCATGGACGAGCTGTACAAG
**P2A (66bp)**
GGCAGTGGAGCAACCAACTTCAGCCTGCTGAAGCAGGCAGGCGACGTGGAAGAAAATCCAGGACCT
**Q8 reporter tag (417bp)**
ATGGGATTGGTTCGCCGAGGAGCGCGAGCAGGTCCCCGGATGCCCCGCGGATGGACTGCCCTGTGCCTGCTTTCCCTCCTTCCAAGCGGCTTCATGGCAGAATTGCCAACTCAA GGTACTTTCTCAAATGTTTCCACGAACGTTAGTCCAGCTAAACCCACTACAACACCTGCACCTAGACCCCCGACGCCAGCCCCTACTATAGCTTCTCAACCACTGAGTCTTCGGCC AGAGGCATGCAGGCCCGCAGCAGGTGGGGCAGTGCACACAAGGGGTCTCGATTTTGCTTGTGACATTTATATTTGGGCTCCTTTGGCAGGGACCTGTGGGGTACTTCTGCTGA GTCTGGTGATCACGTTGTATTGTAACCACAGGAATAGGCGGCGGGTTTGTAAATGTCCACGACCGGTTGTCTGA
**sPA (49bp)**
AATAAAAGATCTTTATTTTCATTAGATCTGTGTGTTGGTTTTTTGTGTG
**AAVS1 gRNA**
GGGGCCACTAGGGACAGGAT
**AP1M1 gRNA**
CCGCCAGCGCCGTCTACGTG
**SNX18 gRNA**
GCGCTACAAGCACTTCGACT
**RPLP0 gRNA**
GCCGTGATGCCCAGGGAAGA
**CHST11 gRNA**
TTGACATCGTCCCCTTTGCG
**IGSF9B gRNA**
ACGGCAGCCTGACAGTGACA
**CHD1 gRNA**
GAATAGTCCGTGGTCTTCCA
**RBM12 gRNA**
AATAATCCATCACCCAGTGT
**SCAF11 gRNA**
GTAAGTGCCTCTGCCACGGT
**SH3BP1 gRNA**
GAATTCTTGGTTGCCTGACT

HDRT: Homology Directed Repair Template, bp: base pairs

Supplementary Table 4. Primer and probe sequences

HDRT amplification primers	Orientation	Sequence
AAVS1_5‘HA_FWD	FWD	ACCGTTTTTCTGGACAACCCC
AAVS1_3‘HA_REV	REV	AGAACTCAGGACCAACTTATTCTGATTTTGT
AP1M1_5‘HA_FWD	FWD	GTCGGTTTCACCACCAGGC
AP1M1_3‘HA_REV	REV	CTCGCCACCCCCGGC
CHD1_5‘HA_FWD	FWD	TTATTGATCAATGTTTCTTCACAGTTCTGATCTCC
CHD1_3‘HA_REV	REV	TAACTTTGGGAGTCATAAACACCGTGAG
CHST11_5'HA_FWD	FWD	AAGCCGTAAGCGGAGGGT
CHST11_3'HA_REV	REV	GCATGATTCTCTCCCTCTCCCC
IGSF_5'HA_FWD	FWD	AAGCCCATTGTCACCTGGC
IGSF_3'HA_REV	REV	AGGGAGGCGGAGACATGG
RPlP0_5'HA_FWD	FWD	AGCGCTATCCGCGGTTTCT
RPlP0_5'HA_REV	REV	GCAGGAGAATCGCTTGAACCTG
RBM12_5'HA_FWD	FWD	TGTGGTTTCAGCGCAGCATG
RBM12_3'HA_REV	REV	ATTCATAGGTGCTCCAGAGCCATT
SCAF_5'HA_FWD	FWD	GGTCACCCAACAAAGACACTCCAC
SCAF_3'HA_REV	REV	TCATACAAATAACATATTAAATTTATAATTCTTCAAATAAAACAGTATCAATAAACCAACAAG
SH3BP1_5'HA_FWD	FWD	CCACACCAGGAGGATGCTG
SH3BP1_3'HA_REV	REV	CTTCATTTATTCATTCACTCGCTCGCTC
SNX18_5'HA_FWD	FWD	CTAGATCGACGGCTCGTCTTC
SNX18_3'HA_REV	REV	GTTGGCCGTGTGGTTGAGC
**PAM frequency/ CRISPR cutting (out/out)**	**Orientation**	**Sequence**
AAVS1_PAM freq. /Cutting_FWD	FWD	GGCATCTCTCCTCCCTCA
AAVS1_PAM freq./Cutting_REV	REV	TGGGGACTAGAAAGGTGAAG
AP1M1_PAM freq./Cutting_FWD	FWD	TCCAAACGCCAAAACGGAGA
AP1M1_PAM freq./Cutting REV	REV	CGGCATTAGTAGGTGCTCCC
CHD1_PAM freq./Cutting_FWD	FWD	GCTCAAGTTATCTTCCTGCCTTGGC
CHD1_PAM freq./Cutting_REV	REV	GCCTGGCCAATCTTACCTCATCTC
CHST11_PAM freq./Cutting_FWD	FWD	AGCCAAGTGGAAATGTTTCAGG
CHST11_PAM freq./Cutting_REV	REV	AGAATGGTGGCATCCATACACA
GBA2_PAM freq./Cutting_FWD	FWD	TGCCTGGGAATATGGGGAGA
GBA2_PAM freq./Cutting_REV	REV	CTAGTCCACACCCAACACCC
IGSF9b_PAMfreq./Cutting_FWD	FWD	CCCTGATGCTACAGGCACAA
IGSF9b_PAM freq. _REV	REV	CACAGGTCCTCATGGCCG
IGSF9b_Cutting_REV	REV	CTCTGCCAAATGCCCACTGT
RBM12_PAM freq. /cutting_FWD	FWD	GCAAGGCTTGGTATGATGCG
RBM12_PAM freq. _REV	REV	GGAGGAACTGGAATTGGGGG
RBM12_Cutting_REV	REV	GGCAGGGCTAACTTCCACAT
RPLP0_PAM freq./Cutting_FWD	FWD	AATTATGTCTCAGCTCCACGTCAT

RPLP0_PAM freq./Cutting_REV	REV	CTCAAAGGCCATCCAAAAGTCA
SCAF11_PAM freq. /Cutting_FWD	FWD	GTCCACGAGGAAATGCGGT
SCAF11_PAM freq._REV	REV	AAGTCTTTCTCACGCCCAG
SCAF11_Cutting_REV	REV	TTTGGCAAGGTTTGGAGCAG
SNX18_PAM freq./Cutting_FWD	FWD	GAGCTCTCATTCACCGCACT
SNX18_PAM freq./Cutting_REV	REV	GATGTCCGGGAAGTTAGCCA
**dPCR Primers and probes: Transgene CNV (In/In) Assays**		
CXCL10_Transgene_FWD	FWD	GGAAAAGCTCGAGATCATCC
CXCL10_Transgene_REV	REV	TCTTTTTCATTGTGGCGATGATT
CXCL10_Transgene_Probe	Probe	6FAM-CTGCTTCTCAGTTCTGCCCTAGAGTGG-BHQ1
CXCL11_Transgene_FWD	FWD	CTACAGTTGTTCAAGGCTTC
CXCL11_Transgene_REV	REV	GGTACATTATGGAGGCTTTC
CXCL11_Transgene_Probe	Probe	6FAM-ACGCTGTCTTTGCATAGGCCC-BHQ1
IFNG_Transgene_FWD	FWD	AAGGAAGAAAGCGATCGAAA
IFNG_Transgene_REV	REV	ATAGATTGGTCGTCCTTGAA
IFNG_Transgene_Probe	Probe	6FAM-TGCAATCCCAGATCGTGAGCTTCT-BHQ1
**dPCR Primers and probes: Site-specific knock-in (Out/In) Assays**		
Ef1a_ Out/In _REV	REV	GCACTTATATACGGTTCTCC
AAVS1_Out/In_FWD	FWD	GCCGTCTTCACTCGCTG
AAVS1_Out/In _Probe	Probe	6FAM-TCCCTTGCGTCCCGCCTCCCCTT-BHQ1
CHD1_Out/In_FWD	FWD	GAACTCCTGGGCTCAAGTTATC
CHD1_Out/In_Probe	Probe	6FAM-ATGCTGGGATTACAGGCATGAGCT-BHQ1
RPLP0_Out/In_FWD	FWD	TATCCAATGGTTGCCTGTAT
RPLP0_ Out/In_Probe	Probe	6FAM-CTATTGGCTGCGCCATCGCCCGT-BHQ1
**dPCR Primers and probes: Endogenous Control Assay**		
AFF3 FWD	FWD	CACCTAGCATGTGTGGCATT
AFF3 REV	REV	GCAGATCCAGGTCGTTGAAG
AFF3 probe	Probe	HEX-AACAACTCTTTCTGTCCCCCT-BHQ1

HDRT: Homology Directed Repair Template, FWD: forward, REV: reverse, freq.: Frequency, dPCR: digital PCR, BHQ1: Black Hole Quencher 1.

Supplementary Table 5. Equipment and Consumables used

Item	Catalogue n°	Provider
Equipment		
4D-Nucleofector® core unit		Lonza Bioscience
4D-Nucleofector® X Unit		Lonza Bioscience
C1000 Touch Thermal cycler		Bio-Rad
Centrifuge 5415R		Eppendorf GmbH
Centrifuge 5424		Eppendorf GmbH
Centrifuge 5427R		Eppendorf GmbH
Centrifuge 5810 R		Eppendorf GmbH
Duomax 1030, platform shaker		Heidolph instruments
Electronic Balance ABS 80-4		Kern & Sohn GmbH
Electronic Rotary Microtome HM340E		Biotek
Erlenmeyer flasks		Thermo Scientific
FlowCytometer LSR-Fortessa X-20		BD Biosciences
Fortessa Aria Cell sorter		BD Bioscience
Fortessa X-20		BD Bioscience
Freezer (-20°C) LCv4010		Carl Roth
Freezer (-80°C), HeraFreeze T series HFU400TV63		Thermo Scientific
GloMaxR Multi		Promega
Heat controlled pressure cooker		Dako
Ice machine Manitowoc SOTTO		Manitowoc
Incubator HERAcell 240i CO2		Thermo Scientific
Laminar Airflow Bench HERA safe 2020 KSP18		Thermo Scientific
Luminometer GloMaxR- Multi+Microplate Multimode Reader with InstinctR E8032		Promega
Magnetic Shaker RH basic		IKA Laboratoy Equipment
Manual system microscope Olympus BX43		Olympus
Microscope Axio Vert.A1		Zeiss
Millipore Barnstead MicroPure		Thermo Scientific
NanoDrop 2000		Thermo Scientific
Neubauer counting chamber		Brand
PCR workstation Pro		VWR Peqlab
pH-meter Five Easy Le409		Mettler Toledo

Pipette filler pipetusR		Hirschmann Labortechnik
Pipette multipipetteR stream		Eppendorf GmbH
Pipettes (2.5 - 1000 µl)		Eppendorf GmbH
QuadroMACS		Miltenyi
Qubit fluorometer		Thermo Scientific
QIAcuity Digital PCR System (6-plex)		Qiagen
Shandon Excelsior ES		Thermo Scientific
Spectrophotometer EPOCH		BioTek Instruments
StepOnePlus Real-Time PCR system		Thermo Scientific
Suction pump AC02		Carl Roth
Table centrifuge mini star silverline		VWR
ThermoMixer C		Eppendorf GmbH
Transwell Permeable supports		Costar
Ultracentrifuge Optima L90K		Beckman Coulter
Vortexer Reax top		Heidolph instruments
Vortexer VWRR Galaxy Mini Star		VWR International bvba
Waterbath GFL 1086		GFL Technology
**General consumables**		
8-Well PCR tube strips plus 8 domed caps	strips: 72.985.002, caps: 65.989.002	Sarstedt
96-Well Plate Advanced TC (flat bottom)	655983	Greiner Bio-one
Biosphere® Filter Tip 10 µl	70.1130.210	Sarstedt
Biosphere® Filter Tip 1000 µl	70.762.211	Sarstedt
Biosphere® Filter Tip 100 µl	70.760.212	Sarstedt
Cell culture multiwell plate, 6 well, PS, clear, sterile	657160	Greiner Bio-one
Cell culture plates CELLSTAR®, sterile, white-96-well plates	KL43.1	Roth
CELLSTAR®, TC, lid with condensation rings, sterile	655180	Greiner Bio-one
CryoPure Tube 1.6 ml yellow	72.380.004	Sarstedt
Disposable needles Sterican® long bevel facet, 0.30x12 mm	4656300	B.Braun
Disposable Syringe, Luer, 1 ml	CH030001L	Charina
FACS tubes	352052	BD
Falcon® 10 ml Serological Pipet	357551	Corning
Falcon® 12-well Clear Flat Bottom TC-treated Multiwell Cell Culture Plate	353043	Corning
Falcon® 12-well Clear Flat Bottom TC-treated Multiwell Cell Culture Plate	353043	Corning
Falcon® 15 ml High Clarity PP Centrifuge Tube	352096	Corning
Falcon® 2 ml Serological Pipet	357507	Corning
Falcon® 24-well Clear Flat Bottom TC-treated Multiwell Cell Culture Plate	353047	Corning
Falcon® 24-well Clear Flat Bottom TC-treated Multiwell Cell Culture Plate	353047	Corning
Falcon® 25 ml Serological Pipet	357525	Corning
Falcon® 35 mm TC-treated Easy-Grip Style Cell Culture Dish	353001	Corning
Falcon® 40 µm Cell Strainer	352340	Corning
Falcon® 48-well Clear Flat Bottom TC-treated Multiwell Cell Culture Plate	353078	Corning
Falcon® 5 ml Round Bottom Polystyrene Test Tube	352052	Corning
Falcon® 5 ml Round Bottom Polystyrene Test Tube, with Cell Strainer Snap Cap	352235	Corning
Falcon® 5 ml Serological Pipet	357543	Corning
Falcon® 50 ml High Clarity PP Centrifuge Tube	352070	Corning
FrameStar Fast Plate 96-well semi skirted	4ti-1200	4titude
Injekt Solo-2-piece single-use syringe, 10 ml, Luer Lock	201235	B.Braun
MACS LS Columns	130-042-401	Miltenyi Biotec

MACS MS Columns	130-042-201	Miltenyi Biotec
Pasteurpipette glass, 145mm	500635	Brand
Pasteurpipette glass, 230mm	500636	Brand
Reagent Reservoirs, 25 ml	EKT8.1	Carl Roth
Rotilab®-syringe filters, CA, sterile, 0.45µm	KC71.1	Carl Roth
Sodium butyrate	B5887-1g	Sigma Aldrich
Spitzen Filter Surphob 100 µl (10x96)	VT0230	Biozym
Spitzen Filter Surphob 10 µl lang (10x96)	VT0200	Biozym
Spitzen Filter Surphob 1,250 µl (10x96)	VT0270	Biozym
Stericup	CT92.1	Carl Roth
Sterile, Stainless Steel, Premium Disposable Scalpel, 11PA	03025	Razormed
Transwell Permeable supports	10107341	Costar
Vasco® Nitril blue glove S	9205518	B.Braun
**Chemicals and reagents (general)**		
0.05% Trypsin-EDTA (1x)	25300-096	Gibco
2x HEPES	S0874	Takara
Albumin from bovine serum Fraktion V	8076.3	Carl Roth
Anti-human CD28 antibody	302934	Biolegend
Anti-PE microbeads	130-105-639	Invitrogen Miltenyi
Brefeldin A	#347688	BD Biosciences
CD3 antibody anti-human, pure-functional grade clone OKT3	130-093-387	Miltenyi
CD8 microbeads, human - lyophilized	130-097-057	Miltenyi
CS&T Research Beads	655050	Becton Dickinson
Cut Smart Buffer	NEB#B6004	New England Biolabs
D-Luciferin	122799	Perkin Elmer Inc.
Dimethyl sulfoxide (DMSO)	A994.1	Carl Roth
DISPASE II	D4693-1G	Sigma Aldrich
Dnase I	A3778.0100	AppliChem
Dynabeads Human T-Activator CD3/CD28	11131D	ThermoFisher
Dynabeads Mouse T-Activator CD3/CD28	114530	ThermoFisher
Ethanol Rotipuran 99.8% p.a.	2065.2	Carl Roth
Fetal Bovine Serum (FBS) SUPERIOR	S0615-500ML	Merck
Ficoll Paque Plus	17144002	Cytiva
Flow-Set Pro Fluorospheres	A62492	Beckman Coulter
FlowCheck Beads	A63493	Beckman Coulter
FlowClean Cleaning Agent	A64669	Beckman Coulter
Glycine Buffer ≥ 99% p.a.	3908.3	Carl Roth
H_2O_2 (2N)	339741	Sigma Aldrich
Human IL-15, premium grade	130-095-765	Miltenyi
Human IL-7, premium grade	130-095-362	Miltenyi
Human Fc Receptor Blocking Solution	422301	Biolegend
Hydrocortisone	H0396-100MG	Sigma Aldrich
Ionomycin	10364-1MG	Sigma Aldrich
IsoFlow Sheath Fluid	8546859	Beckman Coulter
Isoflurane (CPS)	1214	Cp-pharma
KAPA HiFi HotStart Ready Mix	7958927001	Roche
L-Glutamine	25020-081	Life Technologies
LE Agarose	240004	Biozym
Live/Dead Fixable Near IR (780) Viability Kit, for 633 nm excitation	L34975	ThermoFisher Scientific
Matrigel	356237	Corning
Monensin	M5273-500MG	Sigma Aldrich
Nuclease-free water	1097705	Invitrogen
Papain from papaya latex buffered aqueous solution	P3128-100MG	Sigma Aldrich
Penicillin-Streptomycin (10,000 U/mL)	18140122	Gibco
PhosSTOP	4906837001	Sigma Aldrich

PMA	P8139-1MG	Sigma Aldrich
Potassium chloride (KCl) ≥ 99.5% p.a.	6781.1	Carl Roth
Potassium dihydrogen phosphate (KH_2PO_4) ≥ 99% p.a.	3904.1	Carl Roth
Potassium hydrogen carbonate ($KHCO_3$) ≥ 99%	X887.1	Carl Roth
Powdered milk, blotting grade	T145.3	Carl Roth
Precision Plus Protein Standards	161-0374	Bio-Rad
Q5 Polymerase	M0491L	New England Biolabs
Retronectin	T1008	Takara
SDS Pellets ≥ 99.9%	CN30.3	Carl Roth
TEMED ≥ 98.5%	2367.1	Carl Roth
Tris Bufferan® ≥ 99.9% p.a.	4855.5	Carl Roth
Trypan Blue Solution 0.4%	15250061	Gibco
Tween®20	9127.2	Carl Roth
Vibrant™ Dio Cell-Labeling Solution	V22886	ThermoFisher
β-Mercaptoethanol 99% p.a.	4227	Carl Roth
**Cell culture media**		
RPMI Medium 1640 (1x)	21875-034	Life Technologies
DMEM (1x) Dulbecco's Modified Eagle Medium	41966052	Life Technologies
Endothelial Cell Growth Medium 2	C-22111/39211	PromoCell
Opti-MEM	31985062	Life Technologies
MCDB 131 Medium	10372019	ThermoFisher
Freezing medium	10% DMSO, 90% fetal calf serum (FCS)	
Tumor cell and HEK 293T cell medium with RPMI	RPMI Medium 1640 (1x), 10% FCS	
Tumor cell medium with DMEM	DMEM (1x), 10% FCS	
T cell (human) medium	RPMI Medium 1640 (1x), 10% FCS, 2 mM L-Glutamin	

**HDRT-generation**		
Q5 Hot Start High-Fidelity 2X Master Mix	M0494S	NEB
NEBuilder HiFi DNA Assembly Master Mix	E2621L	NEB
XL10-Gold Ultracompetent Cells	200315	Agilent
Kapa HiFi Hot Start ready mix	KK2602/7958935001	Roche
AMPure XP, 60 mL	A63881	Beckman Coulter
DynaMag2	12321D	Thermo Fisher Scientific
DpnI	R0176S	New England Biolabs
Effectene Transfection Reagent	301425	Qiagen
**CRISPR knock-in**		
Alt-R® S.p. Cas9 Nuclease V3, 500 µg	1081059	IDT
Poly-L-glutaminsäure Natriumsalz	P4761-100MG	Sigma-Aldrich
SF Cell Line 4D-NucleofectorTM X Kit L	V4XC-2024	Lonza
P3 Primary Cell 4D-NucleofectorTM X Kit L	V4XP-3024	Lonza
**dPCR**		
Pipet-Lite Multi Pipette L8-200XLS+	17013805	Mettler-Toledo
Rainin Pipette Tips TR LTS 200 µL F 960A/10	17014963	Mettler-Toledo
Eppendorf twin.tec® PCR Plate 96, semi-skirted, clean	30128575	Eppendorf
DNA LoBind® Tubes	30108051	Eppendorf
QIAamp DNA Mini Kit (50)	51304	Qiagen
QIAcuity Probe PCR Kit (5 ml)	250102	Qiagen
QIAcuity Nanoplate 26k 24-well (10)	250001	Qiagen
QIAcuity Nanoplate 26k 8-well (10)	250031	Qiagen
QIAcuity Nanoplate 8.5k 24-well (10)	250011	Qiagen
QIAcuity Nanoplate 8.5k 96-well (10)	250021	Qiagen
Nanoplate Seals (11)	250099	Qiagen
Nanoplate Tray	250098	Qiagen
XbaI	R0145S	New England Biolabs
**Incucyte**		

Incucyte® Clearview 96-well Reservoir Plate	4600	Sartorius
Incucyte® Clearview 96-well Plate for Chemotaxis	4582	Sartorius
Recombinant Protein G	101200	Invitrogen (Thermo)
ICAM-1 Protein, Human, Recombinant (ECD, His & hFc Tag)	10346-H03H	SinoBiological
Incucyte® Cytotox Dye for Counting Dead Cells: Green	4633	Sartorius
Incucyte® Nuclight Rapid Red Dye for Live-Cell Nuclear Labeling	4717	Sartorius
**CAR T cells and Cytokine effects**		
CD8⁺ T cell isolation kit, human	130-096-495	Miltenyi
Pan T cell isolation kit, human	130-096-536	Miltenyi
Human CXCL10/IP-10 DuoSet ELISA, 15 Plate	DY266	R&D
Recombinant human CXCL10	300-12-35	Peprotec
Human CXCL11/I-TAC DuoSet ELISA	DY672	R&D
Recombinant Human I-TAC (CXCL11)	300-46	Peprotec
Human IFN-gamma Recombinant Protein, PeproTech®	300-02-100UG	Thermo fisher
Human IFN-gamma ELISA Kit	DIF50C	R&D
Human IL-2 ELISA	555190	Becton Dickinson
pCMV-Rev2 (p13.33)		Takara
Viral Packaging PCHGP-2 (p14.36)		Takara
pCMV-G (p15)		Takara
UltraPure™ BSA (50 mg/mL)	AM2616	Thermo Fisher Scientific
ICAM-1	#10346-H03H	Sino Biological
Protein G	#101200	Thermo Fisher
96-well Essen ClearView Reservoir,	4600, 4601	Sartorius
Effectene	301425	Qiagen
Optimem I	31985070	Gibco

Supplementary Table 6. Antibodies used for flow cytometry and IF Staining

Antibodies	Clone	Dilution	Catalogue no.	Provider
Invitrogen, CD34 Monoclonal Antibody (QBEND/10), PE	537860	1:10	#MA1-10205	ThermoFisher Scientific
CXCR3, PE	G025H7	1:25	353706	Biolegend
CXCR3, AX700	G025H7	1:25	353741	Biolegend
B7H3, APC	MIH42	1:400	351005	Biolegend
B7H3, PE	MIH42	1:400	331606	Biolegend
L1CAM (CD171), APC	REA163	1:100	130-124-024	Miltenyi
L1CAM (CD171), PE	REA163	1:100	130-100-691	Miltenyi
CD14, PerCP-Cy5.5	M5E2	1:200	301824	Biolegend
CD115, PE-Cy7	9-4D2-1E4	1:50	347308	Biolegend
HLA-DR, BV711	L243	1:100	307644	Biolegend
CD163, APC	GHI/61	1:200	333610	Biolegend
CD206, BV421	15-2	1:50	321125	Biolegend
CD64, PE	10.1	1:400	305008	Biolegend
CD86, FITC	BU63	1:50	374204	Biolegend
CD4, FITC	RPA-T4	1:400	357406	Biolegend
CD4, BV421	RPA-T4	1:50	317434	Biolegend
CD8, PerCP	SK1	1:400	344707	Biolegend
CD8, BV605	SK1	1:400	301036	Biolegend
CD3, Ax700	HIT3a	1:400	300324	Biolegend
CD3, FITC	HIT3a	1:400	300306	Biolegend
CD3, PerCP-Cy5.5	HIT3a	1:400	300328	Biolegend
CD137, BV421	4B4-1	1:50	309819	Biolegend
CD137, BV605	4B4-1	1:50	309821	Biolegend
CD25, PE-Cy7	BC96	1:100	302611	Biolegend
CD25, Ax700	BC96	1:25	302622	Biolegend
HLA-ABC, PE-CY7		1:100	311429	Biolegend

FAS, BV421	DX2	1:200	305624	Biolegend
PDL-1, APC	29E.2A3	1:100	329708	Biolegend
EGFRt (Cetuximab), PE		1:100	537860	Invitrogen
Donkey anti-rabbit Alexa 594	polyclonal	1:300	15146896	Invitrogen
Donkey anti-mouse Alexa 488	polyclonal	1:300	15960296	Invitrogen
Goat anti-rabbit Alexa 594	polyclonal	1:500	10266352	Invitrogen
Goat anti-mouse Alexa 488	polyclonal	1:500	10256302	Invitrogen
Human TruStain FcX (Fc Receptor Blocking solution)			422302	Biolegend

Supplementary Table 7. Software used

Software	Use	Provider
FlowJo_v.10.6.2	Analysis of flow cytometry data	FlowJo
Gen5_v2.04	ELISA assay measurement	BioTek
GraphPad PRISM_v8 to v10	Data analysis and graphical presentation	GraphPad
Incucyte 2021B	Analysis of live cell imaging	Essen Bioscience
Primer3Plus	Primer design and adjustment	Free software, Copyright (c) 2006, 2007 by Andreas Untergasser and Harm Nijveen
ProSort_v1.6	FACS Sorter	Bio-Rad
SAP	Patient data collection	SAP SE
SnapGene	Development of cloning strategies, new Plasmids	Dotmatics
StepOne Software_v2.3	qRT-PCR set-up and analysis	Applied Biosystems
VisionCapt_v16.16d	Western Blot visualization	Fusion
Python version 3.11.4.	CancerPAM programming	
Visual Studio Code (Version: 1.80.1)	source-code editor	
Snakemake	Pipeline workflow management system	

Supplementary Table 8. Potential effector cytokines for tumor microenvironment remodeling in neuroblastoma

Cytokine	Immune Function	Potential in NB TME / solid tumors	Expression in NB cell line and patient data	References
**CCL5 (RANTES)**	Chemokine for CCR5; attracts effector T cells, NK cells, and also Tregs/monocytes to some extent.	Context-dependent. NK-/myeloid-derived CCL5 cooperates with CXCL9 to recruit T cells; in some cancers tumor-derived CCL5 can be protumoral (Tregs/MDSC). Consider local/regulated delivery or pairing with Th1 cues.	Expression in cell lines and patient samples	• Huffman AP, et al. JCI Insight. 2020; DOI: 10.1172/jci.insight.137263.<br>• Böttcher JP, et al. Nature. 2018; DOI: 10.1016/j.cell.2018.01.004
**CCL19 / CCL21**	Homeostatic lymphoid chemokines; attract naïve and central memory T cells and dendritic cells via CCR7.	Lymphoid chemokines (CCR7 ligands) organize TLS-like niches, attract naïve/central-memory T cells & DCs; IT CCL21-DC in NSCLC increased tumor CD8⁺ infiltration and systemic immunity.	No expression in cell lines but expression in patient samples	• Lee JM, et al. Clin Cancer Res. 2017; doi: 10.1158/1078-0432.CCR-16-2821<br>• Adachi K, et al. Nat Biotechnol. 2018; DOI: 10.1038/nbt.4086
**CSF2 (GM-CSF)**	Growth and differentiation factor for myeloid cells; mobilizes neutrophils, monocytes, and dendritic cell precursors from bone marrow.	Recruits/expands APCs at the tumor/vaccine site; classic GM-CSF-secreting tumor vaccines (GVAX) improved cross-presentation; clinically used with anti-GD2 in NB regimens.	No expression	• Dranoff G, et al. Proc Natl Acad Sci USA. 1993; DOI: 10.1073/pnas.90.8.3539<br>• Soiffer RJ, et al. J Clin Oncol. 2003; DOI: 10.1038/sj.onc.1206459
**CXCL9 (MIG)**	Chemokine for CXCR3; attracts activated Th1 cells, CTLs, and NK cells.	IFN-γ-inducible CXCR3 ligand; together with CCL5 orchestrates TIL entry; higher CXCL9 can enable anti-PD-L1 responses; macrophage-derived CXCL9/10 are essential for ICB.	No to low expression	• Dangaj D, et al. Cancer Cell. 2019; DOI: 10.1016/j.ccell.2019.05.004<br>• House IG, et al. Clin Cancer Res. 2020; DOI: 10.1158/1078-0432.CCR-19-1868<br>• Seitz S, et al. Br J Cancer. 2022; DOI: 10.1038/s41416-022-01763-0
**CXCL10 (IP-10)**	Chemokine for CXCR3; attracts Th1, Tc, NK cells.	Potently attracts CXCR3⁺ effector CD8⁺/Th1 T cells and NK cells, supports ICB responses; macrophage-derived CXCL10 (and CXCL9) is required for robust anti-PD-1/CTLA-4 efficacy; intratumoral CXCL10 correlates with better immunotherapy outcomes.	No expression	• Susek KH, et al. Frontiers in Immunology. 2018; doi:10.3389/fimmu.2018.02159.<br>• House IG, et al. Clin Cancer Res. 2020; DOI: 10.1158/1078-0432.CCR-19-1868<br>• Reschke R, et al. J Immunother Cancer. 2021;9:e003521. DOI: 10.1136/jitc-2021-003521<br>• Lee JY, et al. Mol Ther Oncolytics. 2022; DOI: 10.1016/j.omto.2022.04.005<br>• Cheng CC, et al. Cancer Immunology, Immunotherapy : CII. 2024; doi:10.1007/s00262-024-03761-y.

Cytokine	Immune Function	Potential in NB TME / solid tumors	Expression in NB cell line and patient data	References
**CXCL11 (I-TAC)**	Chemokine for CXCR3; attracts Th1, Tc, NK cells.	Strong CXCR3 ligand; intratumoral CXCL11 expression (e.g., by oncolytic vectors) enhances effector T-cell trafficking and improves ACT/CAR-T efficacy.	No expression	• Moon EK, et al. OncoImmunology. 2018; DOI: 10.1080/2162402X.2017.1395997<br>• Liu Z, et al. Nat Commun. 2017; DOI: 10.1038/ncomms14754<br>• Wang G, et al. Mol Ther. 2023; DOI: 10.1016/j.ymthe.2022.08.021.
**CXCL12 (SDF-1α)**	CXCL12 (SDF-1α) is a homeostatic chemokine that binds CXCR4/CXCR7 to regulate immune and stromal cell trafficking, typically recruiting lymphoid progenitors, regulatory T cells, and myeloid cells while also promoting angiogenesis and tissue repair.	Unlike CXCL9/10/11, CXCL12 is generally considered pro-tumorigenic: it promotes tumor growth, angiogenesis, metastasis, and preferentially attracts Tregs and MDSCs into the TME while excluding effector T cells. In NB, CXCL12-CXCR4 drives metastasis to bone marrow and poor prognosis. However, therapeutic blockade of CXCL12/CXCR4 (e.g. with AMD3100/plerixafor or engineered decoy molecules) has been shown to increase $CD8^+$ T-cell infiltration and sensitize tumors to immunotherapy. Thus, while not a candidate for overexpression, CXCL12 is a critical axis to inhibit for TME remodeling.	Moderate to high expression in cell lines and patient samples	• Russell HV, et al. Clin Cancer Res. 2004; DOI: 10.1158/1078-0432.CCR-04-1034.<br>• Feig C, et al. Proc Natl Acad Sci USA. 2013; DOI: 10.1073/pnas.1320318110.<br>• Zeng Y, et al. J Exp Med. 2019; DOI: 10.1084/jem.20190344.
**CXCL13**	Chemokine for CXCR5; attracts B cells, T_FH cells, and some T_H1/T_C cells expressing CXCR5.	Drives B-cell/Tfh recruitment and tertiary lymphoid structures (TLS); TLS associate with better survival and anti-PD-1 response in multiple tumors.	No expression	• Cabrita R, et al. Nature. 2020; DOI: 10.1038/s41586-019-1914-8<br>• Helmink BA, et al. Nature. 2020; DOI: 10.1038/s41586-019-1922-8
**Flt3L (FLT3 ligand)**	Key growth factor for dendritic cells (especially cDC1) and other hematopoietic progenitors; expands DC populations in vivo.	Expands cDC1/cDC subsets in situ, enabling cross-priming; in-situ vaccination (Flt3L + TLR3 agonist ± RT ± PD-1) increases intratumoral DCs and systemic T-cell responses.	Moderate expression in cell lines<br><br>Low Expression in patient samples	• King GD, et al. Neuro Oncol. 2008; DOI: 10.1215/15228517-2007-045<br>• Marron TU, et al. Clin Lymphoma Myeloma Leuk. 2016; NCT01976585
**IFN-α/β (Type-I)**	Innate anti-viral cytokines; activate dendritic cells and macrophages, enhance antigen presentation, and have direct anti-proliferative effects on tumors.	Critical for cDC1 activation & cross-priming in situ; STING agonism or viral sensing induces type-I IFN → turns "cold→hot," improves ICB.	No expression	• Fuertes MB, et al. J Exp Med. 2011; DOI: 10.1084/jem.20101159<br>• Corrales L, et al. Cell reports, 2015; DOI: 10.1016/j.celrep.2015.04.031<br>• Cheng CC, et al. Cancer Immunology, Immunotherapy : CII. 2024; doi:10.1007/s00262-024-03761-y.
**IFN-γ (IFNG, Type-II)**	Central Th1 cytokine; activates macrophages, NK and CTLs; upregulates antigen presentation.	Upregulates HLA/MHC-I and antigen-processing machinery on NB cells (a key NB immune-evasion node), improving CTL recognition; orchestrates Th1 chemokines (e.g., CXCL9/10/11).	No Expression	• Reid GSD, et al. J Transl Med. 2009; DOI: 10.1158/1078-0432.CCR-09-0829<br>• Propper DJ, et al. Clin Cancer Res. 2003; PMID: 12538455
**IL-2**	T cell growth factor; drives proliferation and activation of T cells and NK cells (also expands Tregs at low dose).	Expands effector T/NK cells; historic COG regimen (anti-GD2 + GM-CSF + IL-2 + isotretinoin) improved EFS vs isotretinoin alone; *Note*: later SIOPEN showed no added benefit of IL-2 with dinutuximab beta (toxicity ↑). However, intertumoral IL-2 secretion could improve immunotherapies.	No expression	• Yu AL, et al. N Engl J Med. 2010; DOI: 10.1056/NEJMoa0911123<br>• Ladenstein R, et al. Lancet Oncol. 2018; DOI: 10.1016/S1470-2045(18)30578-3
**IL-7**	Homeostatic cytokine for T cells; maintains naïve and memory T cell survival and restores T-cell numbers after lymphopenia.	Supports T-cell survival/homeostasis; co-expression with CCL19 in CAR-T ("7×19") enhances infiltration and persistence in solid tumors.	Moderate expression in cell lines and patient samples	• Adachi K, et al. Nat Biotechnol. 2018; DOI: 10.1038/nbt.4086.<br>• Pang N, et al. Signal Transduct Target Ther. 2021; DOI: 10.1186/s13045-021-01128-9
**IL-12**	Potent Th1-polarizing cytokine; activates NK and CD8 T cells; induces IFN-γ production.	Potent Th1-polarizer; increases intratumoral IFN-γ, CXCL9/10 and $CD8^+$ T-cell infiltration; localized delivery (IT plasmid/OV) overcomes systemic toxicity and sensitizes to PD-1.	No to very low expression	• Greaney SK, et al. Cancer Immunol Res. 2020; DOI: 10.1158/2326-6066.CIR-19-0359<br>• Ge Y, et al. J Immunother Cancer. 2020; DOI: 10.1136/jitc-2020-000710
**IL-15**	Cytokine that supports NK cell and memory CD8^+ T cell development and survival (shares common γ_c with IL-2 but ***does not*** strongly expand Tregs).	Expands & sustains NK and $CD8^+$ cells; enhances ADCC to anti-GD2 in NB vs IL-2; IL-15 superagonists (N-803/ALT-803) increase intratumoral $CD8^+$ infiltration and show clinical activity.	Moderate expression in cell lines and patient samples	• Nguyen R, et al. Clin Cancer Res. 2020; DOI: 10.1158/1078-0432.CCR-19-1045<br>• Mathios D, et al. J Immunother Cancer. 2016; DOI: 10.1002/ijc.29686<br>• Foltz JA, et al. Clin Cancer Res. 2021; DOI: doi: 10.1158/1078-0432.CCR-20-4575
**IL-18**	IFN-γ-inducing factor; works synergistically with IL-12 or IL-2 to activate NK and T cells.	Boosts Th1/NK function; in NB models IL-18 (±IL-2) reduces metastases and prolongs survival.	No to low expression	• Redlinger RE Jr, et al., Journal of Pediatric Surgery, 2003; DOI: 10.1053/jpsu.2003.50020<br>• Heuer JG, et al., Journal of Immunotherapy, 1999; DOI: 10.1097/00002371-199907000-00003
**IL-21**	Immunomodulatory cytokine (γ_c-family) produced by T_FH and Th17; enhances CD8^+ T	Enhances $CD8^+$ and NK cytotoxicity, can reduce Treg suppression; arming OVs or cells with IL-21 increases intratumoral effector cells.	No expression	• Sutherland AP, et al., Journal of Immunology, 2013; doi:10.4049/jimmunol.1201730.

Cytokine	Immune Function	Potential in NB TME / solid tumors	Expression in NB cell line and patient data	References
	and NK cell function, and can limit Treg and B-cell differentiation.			• Søndergaard H, et al. Journal of Immunotherapy, 2010; doi:10.1097/CJI.0b013e3181c0c1cb.<br>• Chen T, et al., Journal for Immunotherapy of Cancer. 2021; doi:10.1136/jitc-2020-001647.
**TNF-α (low-dose; vessel-targeted)**	Pro-inflammatory cytokine; activates endothelial cells and leukocytes, mediates tumor cell apoptosis at high concentrations.	At low doses/targeted delivery, normalizes abnormal tumor vessels, induces HEV-like endothelium, facilitates CD8⁺ infiltration, and improves vaccination/ACT efficacy.	No to very low expression	• Johansson A, et al. Proc Natl Acad Sci USA. 2012; DOI: 10.1073/pnas.1118296109<br>• Porcellini S, et al. J Exp Clin Cancer Res. 2015; DOI: 10.1080/2162402X.2015
**TNFSF14 (LIGHT)**	TNF-superfamily ligand that binds LTβR and HVEM; imitates lymphotoxin-β and TNF-α signaling in stromal cells.	Normalizes vessels and induces intratumoral HEVs, greatly facilitating lymphocyte entry; LIGHT within tumors/TLS correlates with improved CAR-T trafficking and antitumor immunity.	No to very low expression	• He B, et al. J Pathol. 2018; DOI: DOI: 10.1002/path.5080<br>• Zhang N, et al. J Immunother Cancer. 2023; DOI: 10.1016/j.ymthe.2023.06.015
**XCL1 (lymphotactin)**	Chemokine for XCR1; attracts XCR1^+ conventional dendritic cells type 1 (cDC1).	Recruits XCR1⁺ cDC1, the cross-presenting DC subset essential for CD8⁺ T-cell priming; can be co-delivered with CAR-T/ACT to boost endogenous responses.	No expression	• Böttcher JP, et al. Nature. 2018. DOI: 10.1016/j.cell.2018.01.004<br>• Li XN, et al. J Immunother Cancer. 2025; DOI: 10.1136/jitc-2024-010581

ADCC - Antibody-dependent cellular cytotoxicity; ACT - Adoptive cell transfer; APC - Antigen-presenting cell; CAR T - Chimeric antigen receptor T cell; cDC1 - Conventional type 1 dendritic cell (cross-presenting DC subset); CTL - Cytotoxic T lymphocyte (CD8⁺ T cell); DC - Dendritic cell; EFS - Event-free survival; GD2 - Disialoganglioside (tumor-associated antigen in neuroblastoma); HEV - High endothelial venule; HLA-I / MHC-I - Human leukocyte antigen class I / Major histocompatibility complex class I; HLA-II / MHC-II - Human leukocyte antigen class II / Major histocompatibility complex class II; ICAM / VCAM - Intercellular adhesion molecule / Vascular cell adhesion molecule; ICB - Immune checkpoint blockade; IT - Intratumoral; LTβR - Lymphotoxin beta receptor; MDSC - Myeloid-derived suppressor cell; NB - Neuroblastoma; NK cell - Natural killer cell; OV - Oncolytic virus; PBMC - Peripheral blood mononuclear cell; PD-1 / PD-L1 - Programmed death-1 receptor / Programmed death-ligand 1; PMID - PubMed identifier; RCT - Randomized controlled trial; SIOPEN - International Society of Paediatric Oncology European Neuroblastoma Group; STING - Stimulator of interferon genes (innate sensor); Tfh - Follicular helper T cell; TIL - Tumor-infiltrating lymphocyte; TLS - Tertiary lymphoid structure; TME - Tumor microenvironment; Treg - Regulatory T cell.
