# Supplementary material for: Personalized CRISPR knock-in cytokine gene therapy to remodel the tumor microenvironment and enhance CAR T cell therapy in solid tumors
Source: Nat Commun. 2025 Dec 9;16:10987. doi: 10.1038/s41467-025-67328-w (PMC12689812; doi:10.1038/s41467-025-67328-w)
Supplement: Supplementary file 2 — Reporting Summary [file 41467_2025_67328_MOESM2_ESM.pdf]

nature portfolio

Corresponding author(s): Dr. med. Michael Launspach
michael.launspach@charite.de

Last updated by author(s): Nov 9, 2025

Reporting Summary

Nature Portfolio wishes to improve the reproducibility of the work that we publish. This form provides structure for consistency and transparency in reporting. For further information on Nature Portfolio policies, see our Editorial Policies and the Editorial Policy Checklist.

Statistics

For all statistical analyses, confirm that the following items are present in the figure legend, table legend, main text, or Methods section.

n/a	Confirmed	
☐	☒	The exact sample size (*n*) for each experimental group/condition, given as a discrete number and unit of measurement
☐	☒	A statement on whether measurements were taken from distinct samples or whether the same sample was measured repeatedly
☐	☒	The statistical test(s) used AND whether they are one- or two-sided<br>*Only common tests should be described solely by name; describe more complex techniques in the Methods section.*
☐	☒	A description of all covariates tested
☐	☒	A description of any assumptions or corrections, such as tests of normality and adjustment for multiple comparisons
☐	☒	A full description of the statistical parameters including central tendency (e.g. means) or other basic estimates (e.g. regression coefficient) AND variation (e.g. standard deviation) or associated estimates of uncertainty (e.g. confidence intervals)
☐	☒	For null hypothesis testing, the test statistic (e.g. *F*, *t*, *r*) with confidence intervals, effect sizes, degrees of freedom and *P* value noted<br>*Give P values as exact values whenever suitable.*
☐	☒	For Bayesian analysis, information on the choice of priors and Markov chain Monte Carlo settings
☐	☒	For hierarchical and complex designs, identification of the appropriate level for tests and full reporting of outcomes
☐	☒	Estimates of effect sizes (e.g. Cohen's *d*, Pearson's *r*), indicating how they were calculated

Our web collection on statistics for biologists contains articles on many of the points above.

Software and code

Policy information about availability of computer code

Data collection	Data collection did not involve custom code. Standard instrument software was used: Illumina BaseSpace (v2.0) - sequencing data collection and primary processing (Illumina).BD FACSDiva (v9.0) - flow cytometry data acquisition (BD Biosciences).I ncuCyte 2021B - live-cell imaging and proliferation analysis (Essen BioScience).VisionCapt v16.16d - Western blot visualization (Fusion).Gen5 v2.04 - ELISA plate reading (BioTek).
Data analysis	Data pre-processing and analysis of sequencing datasets involved both standard and custom code. Variant calling was performed using MuTect2, and subsequent processing and annotation were conducted using the custom Python-based CancerPAM pipeline (Snakemake workflow integrating CRISPOR, UCSC API, and multi-omics datasets). Additional custom scripts were used for breakpoint-PAM discovery (bkp-PAM analysis). The CancerPAM pipeline and related code are available via Code Ocean and Zenodo (links in the manuscript Code Availability Statement). Other analysis software used: FlowJo v10.6.2 - flow cytometry analysis (FlowJo LLC).GraphPad Prism v8-v10 - statistical analysis and data visualization (GraphPad Software).Python v3.11.4 - development of custom analysis pipelines.CancerPAM (Snakemake workflow integrating CRISPOR, UCSC API, multi-omics annotation).bkp-PAM structural-variant pipeline.Tumor off-target risk model.Snakemake – workflow management (Köster & Rahmann 2012).Visual Studio Code v1.80.1 - source-code editor (Microsoft). Primer3Plus / SnapGene – primer design and cloning (Freeware; Dotmatics).StepOne Software v2.3 - qRT-PCR setup and analysis (Applied Biosystems). ProSort v1.6 - FACS sorting software (Bio-Rad). SAP (SE) - patient data management.<br><br>The CancerPAM bioinformatics pipeline used in this study, including sample data, is available in the Code Ocean repository under accession number 7671597 [https://codeocean.com/capsule/7671597] under DOI: https://doi.org/10.24433/CO.2312035.v180.<br><br>The ecDNA/SV Breakpoint-CRISPR pipeline is available in the Zenodo repository under accession number 17209179 [https://zenodo.org/

records/17209179] under DOI: https://doi.org/10.5281/zenodo.1720917981.

The Python implementation of the CancerPAM statistical tumour off-target risk model is available in the Zenodo repository under accession number 17209430 [https://zenodo.org/records/17209430] under DOI: https://doi.org/10.5281/zenodo.1720943082.

For manuscripts utilizing custom algorithms or software that are central to the research but not yet described in published literature, software must be made available to editors and reviewers. We strongly encourage code deposition in a community repository (e.g. GitHub). See the Nature Portfolio guidelines for submitting code & software for further information.

Data

Policy information about availability of data

All manuscripts must include a data availability statement. This statement should provide the following information, where applicable:

- Accession codes, unique identifiers, or web links for publicly available datasets
- A description of any restrictions on data availability
- For clinical datasets or third party data, please ensure that the statement adheres to our policy

Raw sequencing data from patient samples
The whole-exome and transcriptome sequencing data generated in this study from patient tumour and matched healthy tissue samples (n = 54) have been deposited in the German Human Genome-Phenome Archive (GHGA) under the accession code GHGAS41175626365361 [https://data.ghga.de/study/GHGAS41175626365361]. These data are available under restricted access due to data privacy regulations and ethical requirements related to personal data from a vulnerable patient population. Access to the data is granted only to qualified researchers for non-commercial research use upon approval of a controlled access request. Requests for data access can be submitted via the GHGA Data Portal [https://data.ghga.de] or by contacting ppk-c@charite.de. Applications are reviewed by the Data Access Committee (DAC) at Charité - Universitätsmedizin Berlin in coordination with GHGA, and responses and data access are typically provided within 3-4 weeks. Approved applicants are required to sign a Data Use Agreement (DUA) governed by Charité - Universitätsmedizin Berlin. A copy of the DUA is available upon request from the corresponding author or from the DAC. Users of these data are requested to cite this study in any resulting publications. Data reuse is limited to non-commercial research purposes.

The raw whole-genome sequencing data from 20 neuroblastoma primary tumours with matched controls used to call structural variants and reconstruct extrachromosomal DNA (ecDNA) elements in this study are publicly available in the European Genome–Phenome Archive (EGA) under accession numbers: EGAS00001001308 [https://ega-archive.org/studies/EGAS00001001308], EGAS00001004022 [https://ega-archive.org/studies/EGAS00001004022], and EGAS00001006983 [https://ega-archive.org/studies/EGAS00001006983]. The processed structural variant calls and ecDNA reconstructions supporting this study's conclusions are publicly available in Zenodo under DOI: https://doi.org/10.5281/zenodo.8032024, corresponding to the published whole-genome sequencing dataset of the discovery cohort by Rodríguez-Fos et al., (Cell Genomics, 2023)79.

Cell line sequencing data
Sequencing data generated from neuroblastoma cell lines in this study have been deposited in the NCBI Sequence Read Archive (SRA) under BioProject accession number PRJNA1292401 [https://www.ncbi.nlm.nih.gov/bioproject/PRJNA1292401]. The datasets are publicly available and can be accessed directly through the project webpage or by entering the accession number in the NCBI SRA Run Selector.

DepMap datasets
Publicly available sequencing and dependency datasets from the Cancer Dependency Map (DepMap) were used in this study:
DepMap 23Q4 [https://doi.org/10.25452/figshare.plus.24667905.v2]69;
DepMap 22Q2 [https://doi.org/10.6084/m9.figshare.19700056.v2]68.
DepMap 20Q4 [https://doi.org/10.6084/m9.figshare.13237076.v4]67;

Other data
All other data supporting the findings of this study are available within the article or its Supplementary Information. Source data are provided with this paper.

Research involving human participants, their data, or biological material

Policy information about studies with human participants or human data. See also policy information about sex, gender (identity/presentation), and sexual orientation and race, ethnicity and racism.

Reporting on sex and gender	The dataset was received in anonymized form, and information on participant sex or gender was not available. Sex and gender were not included as study variables because there is currently no evidence that these factors influence the occurrence of CRISPR target sites or gene-editing efficacy and safety. Consequently, sex-specific analyses could not be performed, and these variables were not relevant to the scientific objectives of the study.
Reporting on race, ethnicity, or other socially relevant groupings	No race- or ethnicity-related variables were used, as these characteristics were not relevant to the scientific objectives of the study and were not considered in the analyses. The neuroblastoma patient cohort is likely to reflect the demographic composition of patients treated in Germany, which predominantly includes individuals of European background as well as those of Middle Eastern and, to a smaller degree, African background.
Population characteristics	Human tumour and matched normal tissue samples (n = 54) from patients with neuroblastoma were analysed. The cohort consisted of paediatric and adolescent patients (age range: 1-17 years). The study analyzed genomic and transcriptomic data from pediatric neuroblastoma patients. Basic sample metadata were available through the collaborative research network, but detailed clinical information was not included in the dataset.
Recruitment	No new participant recruitment was conducted for this study. Tumour and matched normal tissue samples were obtained under a previously approved protocol within the collaborative research network, with ethical oversight by Charité –

Universitätsmedizin Berlin (EA2/055/17). Written informed consent was obtained from all patients and their legal guardians for the use of biological material and data in this research. No participant compensation was provided.

Ethics oversight

Ethics approval was obtained from the Ethics Committee of Charité – Universitätsmedizin Berlin (EA2/055/17) for the use and analysis of human tumor and matched normal samples.

Note that full information on the approval of the study protocol must also be provided in the manuscript.

Field-specific reporting

Please select the one below that is the best fit for your research. If you are not sure, read the appropriate sections before making your selection.

☒ Life sciences ☐ Behavioural & social sciences ☐ Ecological, evolutionary & environmental sciences

For a reference copy of the document with all sections, see nature.com/documents/nr-reporting-summary-flat.pdf

Life sciences study design

All studies must disclose on these points even when the disclosure is negative.

Sample size

No formal statistical sample size calculation was performed. Sample sizes for in vitro and in vivo experiments were chosen based on prior studies using comparable models and were sufficient to detect biologically meaningful differences. For animal studies, group sizes (n = 4–10 per group) were determined according to ethical, logistical, and feasibility considerations and are consistent with published CAR T-cell and xenograft studies. Sample sizes for each experiment are specified in the corresponding figure legends and Source Data file.

Data exclusions

No data were excluded from analyses unless samples failed quality control, such as bacterial contamination or insufficient cell yield in vitro, or graft-versus-host disease (GvHD), failed tumor engraftment, or ectopic tumor growth in vivo. All exclusion criteria were pre-established and are described in the Methods section. Details of included and excluded samples are provided in the Source Data file.

Replication

All key in vitro experiments were independently replicated at least twice, using separate biological replicates and independent cell preparations or donors to ensure reproducibility. In vivo experiments were performed in multiple animals per group, and results were consistent across independent experimental runs. All attempts at replication were successful, and no major findings could not be reproduced.

Randomization

For in vivo studies, animals were randomly assigned to treatment groups using balanced designs to control for donor variability. For in vitro studies, experimental conditions were randomly allocated to wells or culture plates.

Blinding

Investigators were not blinded during sample allocation or data acquisition in vitro or computational analyses. For in vivo experiments performed at EPO Berlin-Buch, researchers conducting the procedures were aware that different treatment groups existed but were blinded to group identities during data collection and initial handling. Data analysis and quantification were performed using automated or objective readouts (flow cytometry, digital PCR, bioluminescence imaging) to minimize bias.

Reporting for specific materials, systems and methods

We require information from authors about some types of materials, experimental systems and methods used in many studies. Here, indicate whether each material, system or method listed is relevant to your study. If you are not sure if a list item applies to your research, read the appropriate section before selecting a response.

Materials & experimental systems

n/a	Involved in the study	
☐	☒	Antibodies
☐	☒	Eukaryotic cell lines
☒	☐	Palaeontology and archaeology
☐	☒	Animals and other organisms
☒	☐	Clinical data
☒	☐	Dual use research of concern
☒	☐	Plants

Methods

n/a	Involved in the study	
☒	☐	ChIP-seq
☐	☒	Flow cytometry
☒	☐	MRI-based neuroimaging

Antibodies

Antibodies used

All antibodies used for flow cytometry and immunofluorescence are listed in Supplementary Table 6 with details on clone, dilution, catalogue number, and supplier.

For flow cytometry, monoclonal antibodies included CD34 (PE, clone QBEND/10, Thermo Fisher Scientific #MA1-10205, 1:10), CXCR3 (PE and Alexa Fluor 700, clone G025H7, BioLegend #353706/353741, 1:25), B7-H3 (APC and PE, clone MIH42, BioLegend #351005/331606, 1:400), L1CAM/CD171 (APC and PE, clone REA163, Miltenyi #130-124-024/130-100-691, 1:100), and additional antibodies targeting CD14 (M5E2), CD115 (9-4D2-1E4), HLA-DR (L243), CD163 (GHI/61), CD206 (15-2), CD64 (10.1), CD86 (BU63), CD4 (RPA-T4), CD8 (SK1), CD3 (HIT3a), CD137 (4B4-1), CD25 (BC96), HLA-ABC (W6/32), FAS (DX2), and PD-L1 (29E.2A3), all from

BioLegend, at 1:25–1:400 dilutions. EGFRt was detected using PE-conjugated Cetuximab (clone C225, Invitrogen #537860, 1:100). Fc receptor blocking was performed using Human TruStain FcX (BioLegend #422302).

For immunofluorescence, primary antibodies included rabbit anti-human/mouse CD3 (clone SP7, Abcam #ab16669, 1:100) and mouse anti-human L1CAM (clone UJ127.11, Invitrogen #MA1-46044, 1:500). Secondary antibodies included donkey anti-rabbit Alexa 594 (Invitrogen, Lot# 15146896, 1:300), donkey anti-mouse Alexa 488 (Invitrogen, Lot# 15960296, 1:300), goat anti-rabbit Alexa 594 (Invitrogen, Lot# 10266352, 1:500), and goat anti-mouse Alexa 488 (Invitrogen, Lot# 10256302, 1:500).

All antibodies were commercially sourced and validated for flow cytometry or immunofluorescence applications by the respective suppliers. Lot numbers are provided where available in Supplementary Table 6.

Validation

All primary antibodies were commercially available and previously validated by the manufacturers for flow cytometry and immunofluorescence staining in human cells. They were used according to the manufacturer's protocols. Specificity was confirmed by appropriate isotype controls and by comparing staining patterns between known positive and negative control populations.

Eukaryotic cell lines

Policy information about cell lines and Sex and Gender in Research

Cell line source(s)

Neuroblastoma cell lines SK-N-BE(2)c (male origin) and SK-N-AS (female origin) were obtained from Prof. Michael Claus V. Jensen (Seattle Children's Hospital). HEK293T cells (female origin, ATCC CRL-3216) and Human Umbilical Vein Endothelial Cells (HUVECs) (female origin, ATCC CRL-1730) were obtained from the American Type Culture Collection (ATCC). HUVECs were cultured in Endothelial Cell Growth Medium 2 (PromoCell, Cat# C-22111/39211) and all other cell lines in DMEM high-glucose medium (Gibco) with 10% FCS and 1% penicillin/streptomycin at 37 °C, 5% CO_2. Primary T cells and monocytes were freshly isolated from anonymized healthy donor blood (sex not recorded) under ethics-approved protocols at Charité – Universitätsmedizin Berlin.No cell lines were newly established in this study. Cell line identities were confirmed by short tandem repeat (STR) profiling before use and all cultures were routinely tested for mycoplasma contamination (negative).

Authentication

Cell line identity was verified by short tandem repeat (STR) profiling before experimentation. All lines were morphologically and functionally consistent with published descriptions.

Mycoplasma contamination

All cell lines tested negative for mycoplasma contamination using the HEK-Blue™ Mycoplasma Detection Kit (InvivoGen) according to the manufacturer's instructions.

Commonly misidentified lines
(See ICLAC register)

No commonly misidentified or cross-contaminated cell lines listed in the ICLAC register were used in this study.

Animals and other research organisms

Policy information about studies involving animals; ARRIVE guidelines recommended for reporting animal research, and Sex and Gender in Research

Laboratory animals

All animal experiments were conducted in female NOD.Cg-Prkdc^{scid} Il2rγ^{tm1Sug}/JicTac (CIEA NOG; Taconic Biosciences, Inc.) mice aged 6–8 weeks at the start of the experiments. A total of 24 mice were used for humanization and 48 mice for non-humanized xenograft CAR T-cell experiments. Mice were housed under specific pathogen-free (SPF) conditions in individually ventilated cages with a 12 h light/dark cycle, at an ambient temperature of 23 ± 1 °C and relative humidity of 45–65 %, with ad libitum access to food and water.

Wild animals

The study did not involve wild animals.

Reporting on sex

Only female mice were used to minimize variability in tumour growth kinetics and immune reconstitution related to sex hormones; therefore, sex was not included as an experimental variable in the analyses.

Field-collected samples

The study did not involve field-collected samples.

Ethics oversight

All animal experiments were approved by the Landesamt für Gesundheit und Soziales (LaGeSo), Berlin, Germany under approval number Reg E0023-23 and were conducted in compliance with the German Law on Animal Welfare and institutional biosafety guidelines.

Note that full information on the approval of the study protocol must also be provided in the manuscript.

Plants

Seed stocks	*Report on the source of all seed stocks or other plant material used. If applicable, state the seed stock centre and catalogue number. If plant specimens were collected from the field, describe the collection location, date and sampling procedures.*
Novel plant genotypes	*Describe the methods by which all novel plant genotypes were produced. This includes those generated by transgenic approaches, gene editing, chemical/radiation-based mutagenesis and hybridization. For transgenic lines, describe the transformation method, the number of independent lines analyzed and the generation upon which experiments were performed. For gene-edited lines, describe the editor used, the endogenous sequence targeted for editing, the targeting guide RNA sequence (if applicable) and how the editor was applied.*
Authentication	*Describe any authentication procedures for each seed stock used or novel genotype generated. Describe any experiments used to assess the effect of a mutation and, where applicable, how potential secondary effects (e.g. second site T-DNA insertions, mosiacism, off-target gene editing) were examined.*

Flow Cytometry

Plots

Confirm that:

☒ The axis labels state the marker and fluorochrome used (e.g. CD4-FITC).

☒ The axis scales are clearly visible. Include numbers along axes only for bottom left plot of group (a 'group' is an analysis of identical markers).

☒ All plots are contour plots with outliers or pseudocolor plots.

☐ A numerical value for number of cells or percentage (with statistics) is provided.

Methodology

Sample preparation	Cells were harvested, washed with PBS containing 2% BSA, and stained with fluorochrome-conjugated antibodies for 20–30 min at 4 °C in the dark. After staining, cells were washed twice and resuspended in FACS buffer (PBS + 2% BSA + 2 mM EDTA). Dead cells were excluded using viability dyes (Zombie Aqua or 7-AAD) where indicated.
Instrument	Flow cytometry was performed on BD FACSCanto II and BD LSRFortessa X-20 instruments (BD Biosciences). Cell sorting was conducted using a BD FACSAria Fusion (BD Biosciences).
Software	Data were acquired using BD FACSDiva software (version 9.0) and analyzed with FlowJo v10.9.0 (BD Biosciences).
Cell population abundance	The abundance and purity of sorted populations were assessed by re-analysis of post-sort fractions. Sorted populations consistently showed > 95% purity based on re-acquisition using the same gating strategy.
Gating strategy	Gating was based on forward and side scatter to exclude debris and doublets, followed by live/dead discrimination using viability dyes. Positive and negative populations were defined using unstained and isotype controls. Detailed gating strategies are shown in Supplementary Figures S10, 19, S21, S26, S28, S29 and S31.

☒ Tick this box to confirm that a figure exemplifying the gating strategy is provided in the Supplementary Information.
